# ﻿*Pseudobaeosporoideae*, a new subfamily within the *Tricholomataceae* for the genus *Pseudobaeospora* (*Agaricales*, *Tricholomatineae*) based on morphological and molecular inference

**DOI:** 10.3897/imafungus.16.144994

**Published:** 2025-03-13

**Authors:** Alfredo Vizzini, Giovanni Consiglio, Katarína Adamčíková, Ledo Setti, Slavomír Adamčík

**Affiliations:** 1 Via S. Pietro D’Ollesia 13b, 10053 Bussoleno (Torino), Italy; 2 Department of Life Sciences and Systems Biology, University of Torino, Viale PA Mattioli I-10125, Torino, Italy; 3 Via Ronzani 61, I-40033 Casalecchio di Reno (Bologna), Italy; 4 Department of Plant Pathology and Mycology, Institute of Forest Ecology, Slovak Academy of Sciences Zvolen, Akademická 2, Nitra 94901, Slovakia; 5 Via C. Pavese 1, I-46029 Suzzara (Mantova), Italy; 6 Laboratory of Molecular Ecology and Mycology, Institute of Botany, Plant Science and Biodiversity Center, Slovak Academy of Sciences, Dúbravská cesta 9, Bratislava 84523, Slovakia; 7 Department of Botany, Faculty of Natural Sciences, Comenius University in Bratislava, Révová 39, Bratislava 81102, Slovakia

**Keywords:** *Agaricomycetes*, *Basidiomycota*, *Pseudobaeospora*, taxonomy, *Tricholomataceae*, *Tricholomatoid* clade

## Abstract

Based on molecular and morphological evidence the new subfamily *Pseudobaeosporoideae* of the *Tricholomataceae* is established within the *Tricholomatineae* for accommodating the unique features of *Pseudobaeospora* such as gymnocarpic mycenoid/collybioid habit, small-sized spores with thick and dextrinoid wall, and presence of crassobasidia. Twenty-six *Pseudobaeospora* collections corresponding to eleven species (five types) were newly sequenced. Collections morphologically attributable to *P.oligophylla* (type of the genus) or to *P.pillodii* are here sequenced for the first time: accordingly, *P.oligophylla* is considered as a posterior synonym of *P.pillodii*. Quélet’s original plate is selected as a lectotype for *Collybiapillodii* and a French collection as its epitype collection. *Pseudobaeosporadeceptiva* is described as a new species from Italy very close to *P.pillodii* from which it differs mainly by bigger spores and SSU and LSU rDNA sequences. The presence of *P.pyrifera* in Italy is documented for the first time and *P.mutabilis* is reduced to its later synonym. A neotype is established for *P.jamonii* which is here proved to be an independent species. Finally, a critical review of the characters used for interspecific distinctions in *Pseudobaeospora* was provided.

## ﻿Introduction

*Pseudobaeospora* Singer (Singer 1942), is a genus first established to encompass only a single species, *Baeosporaoligophylla* Singer (Singer 1938), a small violet agaric from Central Asia (Altai Mountains in Russian Federation). *Pseudobaeospora* was differentiated from the amyloid-spored genus *Baeospora* Singer mainly by its small, thick-walled and dextrinoid mature spores (Singer 1942, 1951). Afterwards, several new species have been described or placed in the genus *Pseudobaeospora* all around the world (e.g., Singer 1963, 1969; Horak 1964; Wasser 1980; Rawla and Arya 1991; Aberdeen 1992; Bas 2002, 2003; Arnolds et al. 2004; Desjardin 2004; Vellinga 2009; Schwarz 2012; Desjardin et al. 2014; Wu et al. 2017; Voto and Soop 2018; Craig et al. 2023). Many of these species have not yet been studied molecularly to support their actual placement as members of the genus. One of the most obvious cases of species misclassification is that of *P.lamingtonensis* Aberdeen from Australia (Aberdeen 1992) which, as pointed out by Voto (2009) and Craig et al. (2023), due to the presence of an annulus is a lepiotoid fungus and belongs in *Agaricaceae* Chevall.

For several decades until 1995, only two taxa were known from Europe (Bas 1995), viz. *P.oligophylla* (Singer) Singer (1951) and *P.pillodii* (Quél.) Wasser (1980), which were later synonymized on morphological basis (Horak 1964, 1968; Redhead 1982; Ronikier and Moreau 2007; Voto 2021). The concept of the genus was later emended by Bas (2003) to accommodate not only species with a pileipellis of a cutis type but also those with a pluristratous hymeniderm/ cellulodermic/epithelioid or trichodermic types and species with basidiome surfaces changing colour in KOH. Then, over a few years, many new European species have been described (Bas 1996, 1998, 2002, 2003; Bas et al. 1997, 2002; Bas and Krieglsteiner 1998; Adamčík and Bas 2002; Arnolds et al. 2003; Adamčík and Ripková 2004a; Clémençon and Ayer 2007; Voto 2009, 2018; Adamčík and Jančovičová 2011; Arauzo 2011a, b), mostly on a morphological basis. *Pseudobaeospora* currently includes around 36 species from temperate, subtropical, and tropical regions in Europe, North and South America, central and southern Asia, Africa (see a collection named *Tricholoma* sp. from Cameroon KR819126, sister to *P.lilacina*, in Craig et al. 2023) and Oceania (Australia, Papua New Guinea) (Bas 2002; Voto 2021, https://www.ameronlus.it/chiavi_micologia.php; Craig et al. 2023). These species occur in habitats from sea level to the subalpine (or even alpine) zone, most of these are very rare and only known from very few collections and areas (Bas 2003; Voto 2021).

Thus far, intrageneric classifications and species circumscriptions in *Pseudobaeospora* have relied on morphological characters, mainly on colours of the basidiomes, the differences in the structure of the pileipellis, reactions of the pileus surface in KOH or ammonia, the presence or absence of clamp-connections and hymenial cystidia (cheilocystidia), and spore sizes and shapes (Bas 2002, 2003; Adamčík et al. 2007; Voto 2009, 2018, 2021).

The taxonomic position of *Pseudobaeospora* has long been uncertain. Singer (1942) initially placed the genus in *Tricholomataceae* R. Heim ex Pouzar s.l. (subfamily ’*Marasmioideae*’ Kauffman), but soon afterwards transferred it to *Agaricaceae* Chevall. (followed by Locquin 1952, Horak 1964, and Wasser 2002), at first in tribus *Lepioteae* Fayod close to *Lepiota* (Pers.) Gray sectionSericellae Kühner (Singer 1951, 1963) and later in tribus *Cystodermateae* Singer (Singer 1986). However, Kühner (1980) retained the genus in *Tricholomataceae*, a placement supported and/or accepted by subsequent researchers on a morphological basis (e.g., Bas 1995, 2003; Bon 1999; Adamčík et al. 2007; Vellinga 2009; Voto 2009; Arauzo 2011a, b; Morozova and Popov 2013), although this placement is questioned by Bon (1999), who hypothesized affinities also with the *Mycenaceae* Overeem. In the first pioneering works of the molecular era, the genus has not been included in the analysis of the *Agaricales* Underw. by Moncalvo et al. (2000, 2002) and Matheny et al. (2006). Vellinga (2003, 2004) showed that *P.pyrifera* is not part of the *Agaricaceae*. In subsequent molecular phylogenetic analyses, based on a poor taxon sampling of *Pseudobaeospora* species/collections and not including the type species, the genus showed affinity with *Callistosporium* Singer (Wu et al. 2017), more specifically *Callistosporiaceae* Vizzini, Consiglio, M. Marchetti & P. Alvarado (Vizzini et al. 2020a) or *Tricholomataceae* s.s. (Desjardin et al. 2014; Sánchez-García and Matheny 2017; He et al. 2019; Sánchez-García et al. 2020, 2021; He and Yang 2022) as delimited by Sánchez-García et al. (2014). Both these families belong to the suborder *Tricholomatineae* Aime, Dentinger & Gaya of the *Agaricales* (Dentinger et al. 2015; He and Yang 2022; Vizzini et al. 2024).

The aim of the present study is: I) to specify a phylogenetic placement of the genus *Pseudobaeospora* by a multigene analysis of the suborder *Tricholomatineae* (= Tricholomatoid clade in the sense of Matheny et al. 2006) using a larger taxon sampling for *Pseudobaeospora* than previous studies and including for the first time its type species (*P.oligophylla* = *P.pillodii*); II) to use this multi-loci sampling for the reconstruction of phylogenetic relationships within *Pseudobaeospora* and the circumscription of the genus; III) to confirm identity of recent Italian collections with morphological similarities to *P.pyrifera* (a species so far known only from Germany, The Netherlands, Spain, Russia, France and Slovakia; Bas and Krieglsteiner 1998; Bas 2003; Chaillet et al. 2007; Arauzo 2011a, b; Morozova and Popov 2013; Caillet et al. 2018; Caboň et al. 2021) providing a morphological and molecular phylogenetic circumscription; and IV) to define taxonomic position and circumscription of some *Pseudobaeospora* species based on new sequence and morphological data obtained from authentic material and supplemented by recent collections.

## ﻿Material and methods

### ﻿Morphological analysis

Macromorphological features of *P.deceptiva* and *P.pyrifera* were described from fresh specimens. Colour terms in capital letters (e.g., Deep Vinaceous, Plate XXVII) are those of Ridgway (1912). L = number of lamellae reaching the stipe, l = number of lamellulae between each pair of lamellae. The microscopic structures in both fresh and dried materials were examined, in different mountants: water, L4 [7.2 g KOH, 160 mL glycerine, 840 mL dH_2_O, 7.6 g NaCl and 5 mL Invadin (Ciba-Geigy), Clémençon 1972], Melzer’s reagent, ammoniacal Congo red, and Cotton blue (Singer 1986; Largent and Baroni 1988). Dried fragments of basidiomes were rehydrated in water and observed in L4. All microscopic measurements were carried out under oil immersion at ×1000 with a Zeiss Axioscope 40 compound microscope.

Spore measurements were made by photographing all the spores (taken from the hymenophore of mature specimens) occurring in the visual field of the microscope using Mycomètre software (Fannechère 2011). Spore length, width, and length/width ratio (Q) were measured with exclusion of the hilar appendix and are given as: (minimum–) average minus standard deviation – average – average plus standard deviation (–maximum). The approximate spore volume (V in μm^3^) was calculated as that of an ellipsoid (Gross 1972; Meerts 1999) and provided with the same statistics as the other spore parameters. The notation ‘n/m/p’ provided after spore measurements in descriptions indicates that measurements were made on ‘n’ randomly selected spores from ‘m’ basidiomes of ‘p’ collections. The width of the basidia was measured at the widest part, and the length was measured from the apex (sterigmata excluded) to the basal septum.

Microscopic pictures were taken on a Canon PowerShot A640 digital camera connected to a Zeiss Axioscope 40 compound microscope with both interferential contrast and phase-contrast optics. Herbarium (Fungarium) acronyms follow Thiers (2025). Author citations are from Index Fungorum (http://www.indexfungorum.org/authorsoffungalnames.htm).

### ﻿DNA extraction, amplification, and sequencing

Total DNA was extracted from thirty dry specimens (Suppl. material 1) employing a modified protocol based on Murray and Thompson (1980) or using the E.Z.N.A. Fungal DNA Mini Kit (Omega Bio-Tek, Inc., Norcross, GA, USA) following the manufacturer’s instructions. The following loci were targeted: (i) the internal transcribed spacer regions of nuclear ribosomal DNA (nrITS), (ii) nuclear ribosomal large subunit (nrLSU), (iii) the most variable region between domains 6 and 7 of the nuclear gene encoding the second largest subunit of RNA polymerase II (*RPB2*), (iv) translation elongation factor EF-1 alpha (*TEF1*) and (v) 18S ribosomal RNA (SSU). Primers ITS1F and ITS4 (White et al. 1990; Gardes and Bruns 1993) were employed for the ITS rDNA region, NS19b and NS41 or NS1 and NS4 for SSU (White et al. 1990; Gardes and Bruns 1993; Hibbett 1996), while LR0R and LR5 (Vilgalys and Hester 1990; Cubeta et al. 1991) were used to amplify the LSU/28S rDNA region, EF1-728F, EF1-983F, EF1-1567R and EF1-2218R (Carbone and Kohn 1999; Rehner and Buckley 2005) for the *TEF1* gene, and bRPB2-6F2 (reverse of bRPB2-6R2), bRPB2-7.1R2, bRPB2-7R2, bRPB2-6F and bRPB2-7.1R for the *RPB2* gene (Matheny et al. 2007). Amplification of DNA was performed using a PCR mix consisting of approximately 2 ng/μl of template DNA, forward and reverse primers (10 pmol/μl), 5× HOT FIREPol^®^ Blend Master Mix (Solis BioDyne, Tartu, Estonia) and molecular grade water added up to 20 μl. PCR products were checked in 1% agarose gels, and positive reactions were sequenced with one or both PCR primers. Chromatograms were checked searching for putative reading errors, and these were corrected.

### ﻿Phylogenetic analysis

BLAST (Altschul et al. 1990) was used to select the most closely related sequences from public databases (INSDC/GenBank https://www.ncbi.nlm.nih.gov/genbank/, UNITEhttps://unite.ut.ee/, and BOLD http://www.boldsystems.org/). Three different alignments were built. (1) First, a combined alignment including SSU rDNA, LSU rDNA, *TEF1* (introns excluded) and *RPB2* (introns excluded) sequences from representative species of the major lineages in the *Tricholomatineae* found in previous phylogenetic studies (Matheny et al. 2006; Sánchez-García et al. 2014, 2016, 2021; Bellanger et al. 2015; Alvarado et al. 2015, 2018a, b; Sánchez-García and Matheny 2017; Raj et al. 2019; Vizzini et al. 2020a, 2024; He and Yang 2022; He et al. 2023). (2) Second, a combined alignment of nrITS, SSU rDNA, LSU rDNA, and *RPB2* (introns excluded) included data of *Tricholomataceae* s.s. and *Pseudobaeospora* following Sánchez-García et al. 2014, 2016, 2021; Sánchez-García and Matheny (2017), Corriol and Jargeat (2019), He and Yang (2022) and He et al. (2023). 3) Third, an alignment of all the studied *Pseudobaeospora* collections was supported by ITS sequences retrieved from the public databases (also including the environmental sequences) following Wu et al. (2017), Gisotti et al. (2021) and Craig et al. (2023). *Suilluspictus* (Peck) Kuntze, *Ampulloclitocybeclavipes* (Pers.) Redhead, Lutzoni, Moncalvo & Vilgalys, *Pseudoarmillariellaectypoides* (Peck) Singer, and *Tricholomainamoenum* (Fr.) Gillet were used as outgroup taxa for the first and third alignments, respectively. Rooting was done in FigTree v.1.4.0. (http://tree.bio.ed.ac.uk/software/figtree/). The *Tricholomataceae* s.s. phylogeny was midpoint-rooted.

Sequences (Suppl. material 1) were first aligned in MEGA 6.0 (Tamura et al. 2013) software with its MUSCLE application (Edgar 2004) and then edited manually. Some ambiguously aligned regions, which were characterised by uncertain positions and the presence of introns, were excluded from subsequent analyses (Criscuolo and Gribaldo 2010).

**Figure 1.**
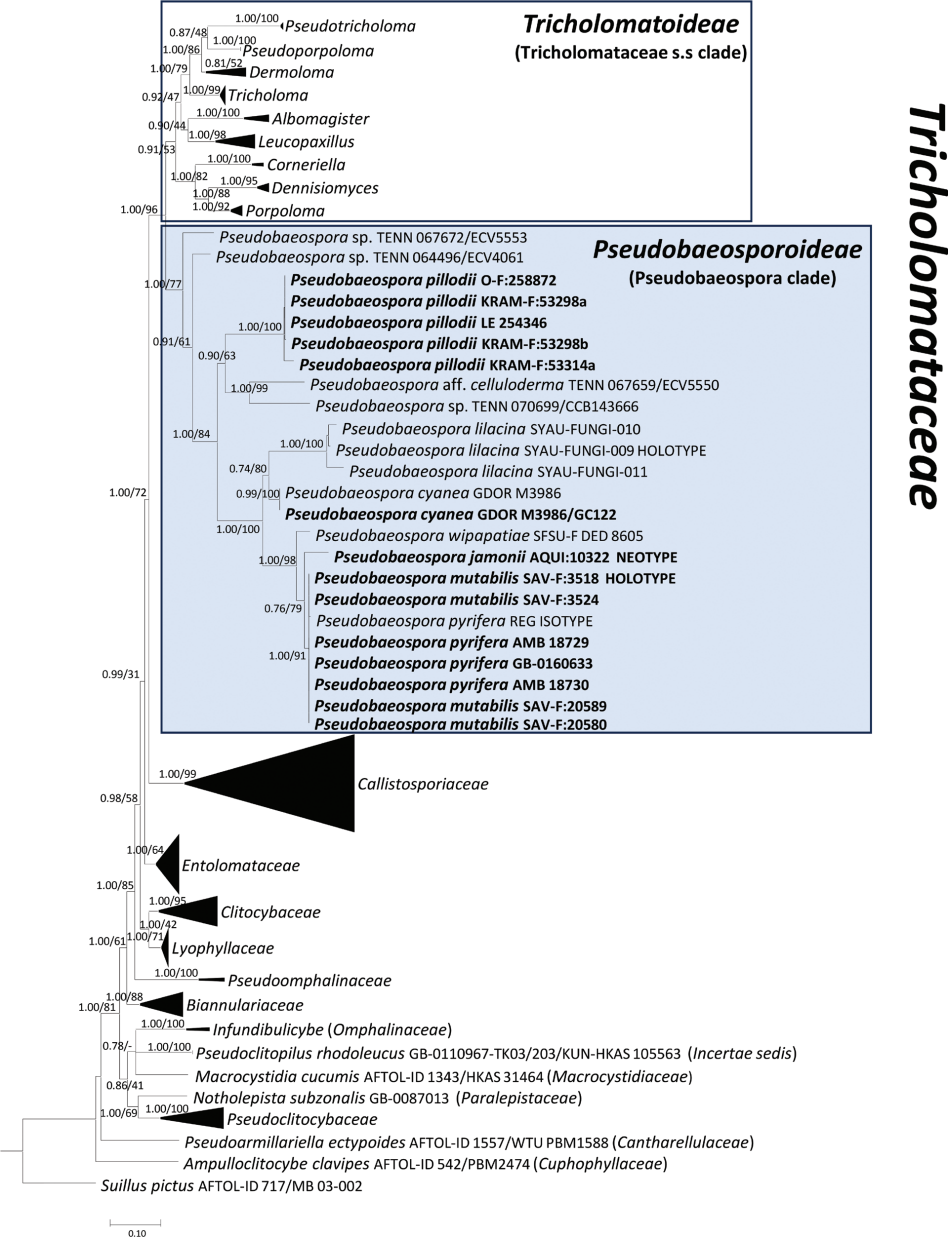
Bayesian inference phylogram built with nucleotide sequence data of four loci (nrLSU, nrSSU, *RPB2*-exons and *TEF1*-exons) of the main lineages inside the suborder *Tricholomatineae* of the order *Agaricales*, rooted with *Suilluspictus* (*Boletales*), *Ampulloclitocybeclavipes* and *Pseudoarmillariellaectypoides* (*Hygrophorineae*, *Agaricales*) as outgroups. Nodes were annotated with Bayesian PP (left) and ML BP (right) values, with the significance threshold considered as Bayesian PP ≥ 0.95 and/or ML BP ≥ 70%. Subsignificant support values were annotated in parentheses. All family-level clades, except for *Tricholomataceae*, were collapsed. Boldface names represent samples sequenced for this study.

**Figure 2.**
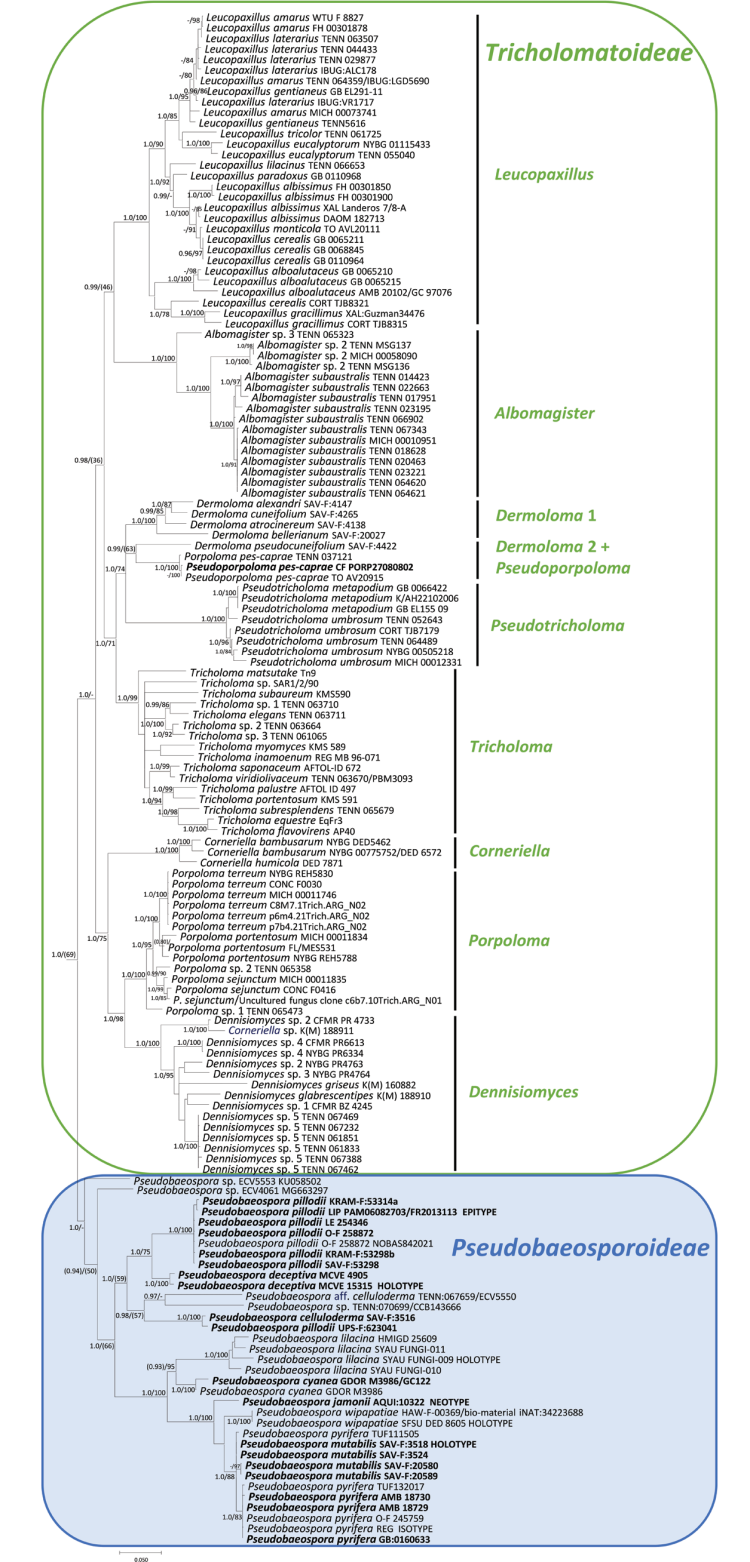
Mid-point rooted Bayesian phylogeny (nrITS, nrLSU, nrSSU, *RPB2*-exons) of the *Tricholomataceae* s.s. Nodes were annotated with Bayesian PP (left) and ML BP (right) values, with the significance threshold considered as Bayesian PP ≥ 0.95 and/or ML BP ≥ 70%. Subsignificant support values were annotated in parentheses. Boldface names represent samples sequenced for this study.

**Figure 3.**
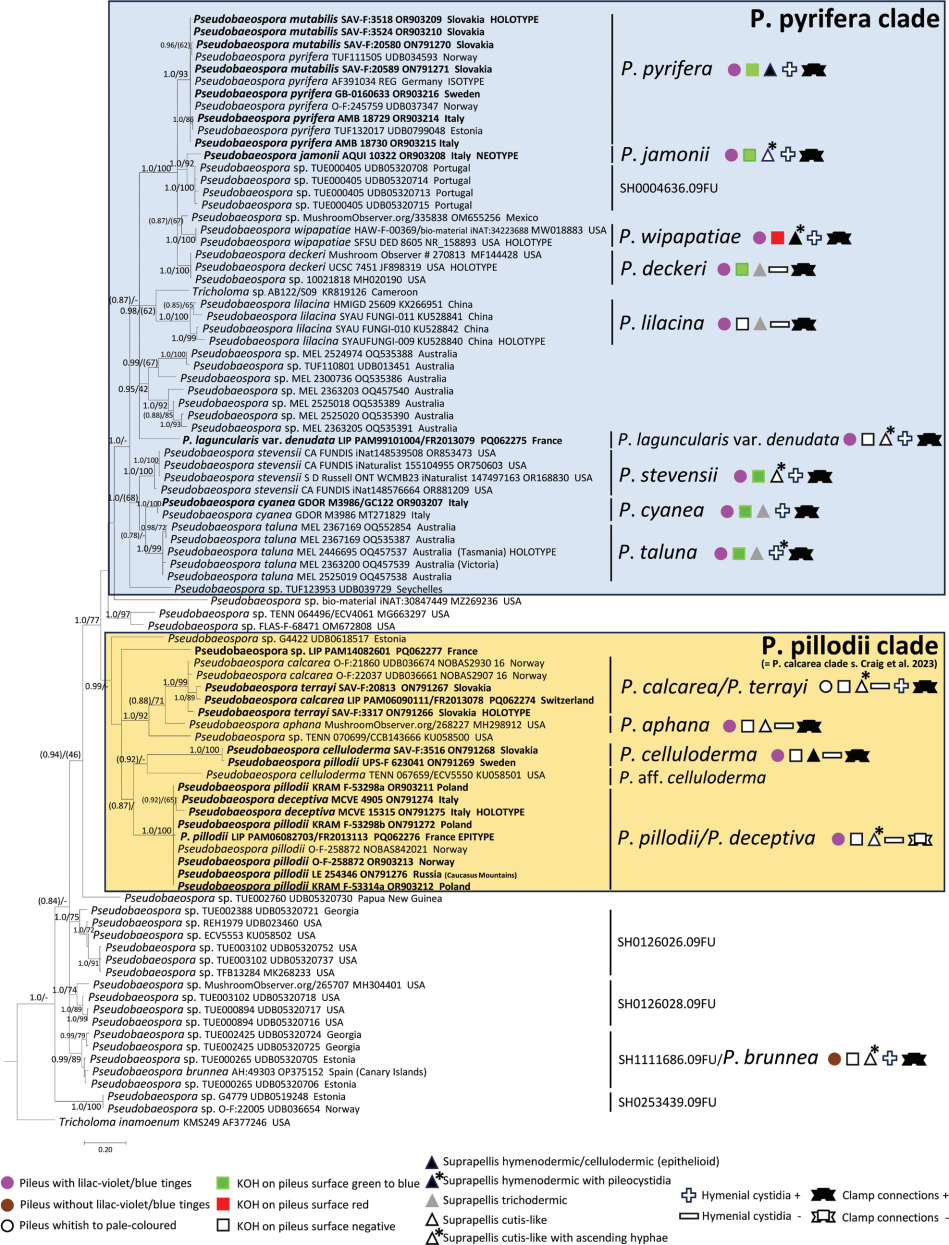
Bayesian inference phylogram built with ITS nucleotide sequence of *Pseudobaeospora*, rooted with *Tricholomainamoenum* (*Tricholomataceae*) as outgroup. Nodes were annotated with Bayesian PP (left) and ML BP (right) values, with the significance threshold considered as Bayesian PP ≥ 0.95 and/or ML BP ≥ 70%. Subsignificant support values were annotated in parentheses. Boldface names represent samples sequenced for this study. Clades are named following Craig et al. (2023).

The Bayesian analyses (BI) were performed through the CIPRES Science Gateway platform (Miller et al. 2010) by using the MrBayes v. 3.2.7 algorithm with ITS, LSU and SSU rDNA, *TEF1*, *RPB2* data partitioned, two simultaneous runs, four chains, temperature fixed at 0.2 and sampling every 1000 generations until reaching the convergence parameters (standard deviation less than 0.01). The first 25% trees were discarded as burn-in. Bayesian analyses reached convergence after 4.530 M (*Tricholomatineae*), 4.860 M (*Tricholomataceae* s.s.), and 1.250 M (*Pseudobaeospora*) generations. Finally, a full search for the best-scoring Maximum likelihood tree was performed in RAxML v.8.2.10 (Stamatakis 2014) using the standard search algorithm (data partitioned as in Bayesian analysis, GTRGAMMA model, 1000 bootstrap replications). As both Bayesian and Maximum likelihood analyses produced similar topologies, only the Bayesian trees with both PP (posterior probability) and ML BP (bootstrap proportions) values were shown (Figs 1–3). Significance threshold was set to PP ≥ 0.95 and ML BP ≥ 70%.

### ﻿Abbreviations

**BI**: Bayesian inference

**CHEGD fungi**: *Clavariaceae*, *Hygrophoraceae*, *Entolomataceae*, *Geoglossaceae* and *Dermoloma*

**DNA**: deoxyribonucleic acid

**INSDC**: International Nucleotide Sequence Database Collaboration

**nrITS**: nuclear ribosomal internal transcribed spacer

**nrLSU**: nuclear ribosomal large subunit ribosomal DNA

**nrSSU**: nuclear ribosomal small subunit ribosomal DNA

**ML**: Maximum likelihood

**ML BP**: Maximum likelihood bootstrap proportion

**PCR**: Polymerase Chain Reaction

**PP**: posterior probability

***RPB2***: DNA-directed RNA polymerase II subunit 2 gene

**SEM**: scanning electron microscope

***TEF1-α***: translation elongation factor 1-α gene

**UNITE**: User-friendly Nordic ITS Ectomycorrhizal Database.

## ﻿Results

### ﻿Molecular phylogeny

A total of 26 *Pseudobaeospora* collections (5 types included) corresponding to 11 species, 3 *Lepista* collections [*L.caespitosa* (Bres.) Singer, *L.densifolia* (J. Favre) Singer & Clémençon, *L.glaucocana* (Bres.) Singer] and *Pseudoporpolomapes-caprae* (Fr.) Vizzini & Consiglio collection were sequenced (Suppl. material 1). The final multigenic alignment of the *Tricholomatineae* is composed of 236 OTUs (collections) and contained 2988 total nucleotide positions: 872 positions from LSU/28S rDNA (236 sequences), 847 positions from SSU/18S rDNA (119 sequences), 514 positions from *TEF1* (77 sequences) and 755 positions from *RPB2* (165 sequences). The final multigenic alignment of the *Tricholomataceae* s.s. is composed of 137 OTUs and contained 3912 total nucleotide positions: 564 positions from ITS rDNA (137 sequences), 872 positions from LSU/28S rDNA (92 sequences), 1718 positions from SSU/18S rDNA (44 sequences) and 758 positions from *RPB2* (51 sequences). The final alignment of *Pseudobaeospora* is composed of 90 OTUs and contained 517 from ITS rDNA total nucleotide positions. Data of all collections used in the phylogenetic analyses are listed in the Suppl. material 1.

In the molecular phylogeny of *Tricholomatineae* (Fig. 1), the *Tricholomataceae* were recovered as monophyletic with strong support (PP 1.00, ML BP 96%). *Tricholomataceae* were sister to *Callistosporiaceae* (PP 1.00, ML BP 72%) as previously pointed out by He et al. (2023) and Vizzini et al. (2024). The Pseudobaeospora clade was strongly supported (PP 1.00, ML BP 77%). This clade is sister (PP 1.00, ML BP 96%) to the clade formed by the remaining taxa of the *Tricholomataceae* (= core of the *Tricholomataceae*, PP 0.91, ML BP 53%) which encompasses another nine monophyletic genus-rank clades corresponding to *Albomagister* Sánchez-García, Birkebak & Matheny, *Corneriella* Sánchez-García, *Dennisiomyces* Singer, *Dermoloma* J.E. Lange ex Herink, *Leucopaxillus* Boursier, *Porpoloma* Singer s.s., *Pseudoporpoloma* Vizzini & Consiglio, *Pseudotricholoma* (Singer) Sánchez-García & Matheny and *Tricholoma* (Fr.) Staude. The non-collapsed phylogenetic tree is available as Suppl. material 2. The same topology was recovered in the phylogeny of *Tricholomataceae* s.s. although the *Dermoloma* and *Pseudoporpoloma* relationships remain unresolved (Fig. 2).

In the phylogenetic analysis of the *Pseudobaeospora* ITS alignment (Fig. 3), two major sister clades within the studied genus were recognized, viz. the *P.pyrifera* clade and the *P.pillodii* clade (= *P.calcarea* clade sensu Craig et al. 2023), as previously unveiled by Craig et al. (2023). *Pseudobaeosporabrunnea* and several environmental sequences are outside the two clades. Six species clades are identified by a position of sequence from type material within the *P.pyrifera* clade, i.e., *P.pyrifera* Bas & L.G. Krieglst., *P.jamonii* Bas, Lalli & Lonati, *P.wipapatiae* Desjardin, Hemmes & B.A. Perry, *P.deckeri* C.F. Schwarz, *P.lilacina* X.D. Yu, Ming Zhang & S.Y. Wu and *P.taluna* S. Craig, L.J. Vaughan & T.W. May. Type of *P.mutabilis* Bas & Adamčík and additional authentic material of the species clustered within a single species-rank clade with sequences of *P.pyrifera* and this is also supported by multigene analysis (Fig. 2), therefore we propose *P.mutabilis* to be its later synonym. All of these species clades with more than one sample were strongly supported. In addition, sequences identified as *P.stevensii* Desjardin (PP 1.00, ML BP 100%) and *P.cyanea* Arnolds, Tabarés & Rocabruna (PP 1.00, ML BP 100%) are placed in two highly supported species-rank clades. A well-defined species is also probably represented by a clade of Portuguese environmental sequences sister to *P.jamonii* (PP 1.00, ML BP 92%) which corresponds to UNITE species hypothesis SH0004636. A topotypical collection of P.laguncularisvar.denudata Bas (LIP PAM99101004 FR2013079, France) occupied an uncertain position within the *P.pyrifera* clade. There was a large cluster of sequences from Australian collections (PP 0.95, ML BP 42%) which probably represents at least two undescribed species. In addition, four singletons with very distant geographical origins (Cameroon, Mexico, Seychelles, USA) probably represent additional undescribed species.

Within the *P.pillodii* clade, samples sequenced in this study are placed in three species clades and one singleton. In addition, we retrieved four independent singletons which may represent distinct species from public databases. Holotype and authentic material of *P.terrayi* Adamčík & Jančovič. are clustered with sequences identified as *P.calcarea* Clémençon & Ayer (Fig. 3), but conspecificity of these names can only be confirmed when *P.calcarea* holotype is also sequenced. European collections of *P.celluloderma* Bas in the *P.celluloderma* clade are sister to a sequence from a USA collection (KU058501, TENN 067659), suggesting that the latter probably represent a different species (P.aff.celluloderma). A pair of two sequences from Italian collections was nested within *P.pillodii* sequences in the ITS tree (Fig. 3) but is clearly supported as distinct species in the multigene analysis where also their SSU and LSU sequences are present (Fig. 2). This clade was described as *P.deceptiva* sp. nov. here.

In summary, we estimated that our ITS dataset represents ca. 36 species globally, of which 13 are European. Fifteen species in our analyses had assigned names, of which nine are European. More than 46% of potential species included in our ITS analyses are represented by a single sample and approximately 58% of species are probably representing new undescribed species.

### ﻿Taxonomy

#### ﻿ Tricholomataceae section Pseudobaeosporoideae

Taxon classificationFungiAgaricalesTricholomataceae

Vizzini, Consiglio & Setti subfam. nov.

94C7169C-5407-56B3-AEFA-231531EFAEE7

857522

##### Diagnosis.

Basidiomes agaricoid (pileostipitate), gymnocarpic (no veils), mostly mycenoid or rarely collybioid, homogeneous (context of stipe and pileus continuous), hymenophore lamellate, lamellae adnexed, decurrent with a tooth to almost free or free, spore deposit white to whitish, basidiospores usually smooth, when mature usually thick-walled, non-amyloid, often weakly to strongly dextrinoid, basidia non-siderophilous, sometimes thick-walled and sclerified (wall 1–3 µm thick, crassobasidia or sclerobasidia) and dextrinoid, hymenophoral trama regular to subregular, hymenial cystidia absent or present as cheilocystidia, rarely as pleurocystidia, caulocystidia usually present, pileipellis a cutis to trichoderm or pluristratous hymeniderm/epithelium/celluloderm, pileocystidia-like elements rare, clamp-connections present or absent, hyphal system monomitic. Terrestrial, trophic mode unknown, presumably saprotrophic or forming an unspecified symbiotic interaction with vascular plants.

##### Type of the subfamily.

*Pseudobaeospora* Singer, Lloydia 5: 129 (1942).

##### Type of the genus.

*Baeosporaoligophylla* Singer, Revue Mycol., Paris 3(4–5): 194 (1938) = *Collybiapillodii* Quél. [as ‘pillodi’], C. r. Assoc. Franç. Avancem. Sci. 18(2): 509 (1890).

##### Representative genus.

*Pseudobaeospora*.

##### Notes.

The subfamily is currently monogeneric and is sister to the core of the *Tricholomataceae* [*Tricholomataceae* subfam. *Tricholomatoideae* (Singer) Bon] (Figs 1, 2) within the *Tricholomatineae*. *Pseudobaeospora* is circumscribed by small mycenoid (e.g., *P.celluloderma*) to collybioid white-spored basidiomes [indicatively, pileus 1.5–30 mm in diam., stipe 10–55(–70) × 0.5–3.0 mm]; pileus frequently with pale to dark lilac, violaceous, purple, blue tinges, hemispherical, obtusely conical or paraboloid to plano-convex or plano-conical (campanulate); lamellae adnexed, emarginate, or decurrent with a tooth to free, usually more or less concolorous with pileus; stipe pruinose to fibrillose, often rooting, at base mostly with white to rarely yellow tomentum and rhizomorphs; basidiospores small (from 2.5 µm to rarely more than 6.5 µm long), subglobose to broadly ellipsoid, colorless, smooth (minutely rugulose under SEM in *P.wipapatiae* Desjardin, Hemmes & B.A. Perry, Desjardin et al. 2014), pore-less, with very distinct, abrupt hilar appendage, at first thin-walled and non-amyloid, then becoming thick-walled and weakly to strongly dextrinoid, congophilous, cyanophilous, and rather frequently more or less metachromatic in Cresyl blue (e.g., *P.paulochroma* Bas, *P.bavariae* Bas); basidia 4-spored to 2-spored, or 4- and 2-spored in the same basidiome, often with a basal clamp-connection, without inner siderophilous granulations, scattered sclerified (thick-walled) dextrinoid basidia (sclerobasidia, crassobasidia following Singer and Clémençon 1972, Watling and Chandra 1983, and Clémençon 2004) often present; pleurocystidia usually lacking (rarely present, e.g., *P.aciculifera* Voto & Soop, *P.cyanea*, *P.taluna*) and cheilocystidia present in some species, in one case with amyloid contents (*P.wipapatiae*); hymenophoral trama regular to somewhat irregular, with elements in central part (mediostratum) often inflated; pileipellis varying from a simple cutis or cutis-trichoderm to a trichoderm in some species to an irregular pluristratous hymeniderm/epithelium/ /celluloderm in others; some terminal elements could be differentiated as pileocystidia; hyphae thin-walled, not or slightly gelatinized; pigments predominantly parietal (sometimes also hyphae with minute extracellular incrustations) but also intracellular (vacuolar or cytoplasmatic); in 5% KOH pileipellis fragments usually changing colour or becoming violet, green, yellow or brownish with such tinges, rarely first red then yellow-green; in several species pseudotissues more or less dextrinoid; caulocystidia usually present at least at stipe apex, thin-walled, scattered to clustered; clamp-connections usually present in several or all tissues, rare in *P.calcarea*, in one species restricted to basidia and subhymenium (*P.frieslandica* Bas) or absent (e.g., *P.pillodii*).

The species show a terrestrial habit, usually on needle carpets of conifers, forest litter, wooden debris, humus, deeply hidden among/on mosses and grasses but also sometimes on bare soil (Bas 2002, 2003). Their trophic status remains unknown, presumably saprotrophic, non-ectomycorrhizal (Bas 2002, 2003; not reported in Rinaldi et al. 2008, Tedersoo et al. 2010, and Tedersoo and Smith 2013). As noted by Ronikier and Moreau (2007), most species appear to prefer calcareous and/or nutrient-rich soils, but some are found on peaty soils.

The unique combination of small-sized mycenoid to collybioid basidiomes often with lilac violet tinges, pileipellis usually positively reacting with KOH, presence of scattered dextrinoid thick-walled basidia and small-sized spores becoming secondarily thick-walled and dextrinoid makes this genus easily identifiable and delimitable. Thickening spore walls becoming dextrinoid typically occurs also in the genus *Rhodocollybia* Singer (*Omphalotaceae* Bresinsky, *Marasmiineae* Aime, Dentinger & Gaya) (Antonín and Noordeloos 1997, 2010) and is considered a generic character. *Rhodocollybia* however differs in larger spores, larger basidiomes with a simple ixocutis, absence of crassobasidia, a pinkish yellow to pinkish brown, never white spore deposit, and a different (contradictory) trophic habit, viz. putatively EcM (Pera and Alvarez 1995; Mleczko 2004; Schirkonyer et al. 2013) to facultatively biotrophic saprobe (secondary colonizers of senescent EcM root tips, Tedersoo et al. 2010; Tedersoo and Smith 2013).

Crassobasidia (non-dextrinoid) are occasionally present in different suborders of *Agaricales*: *Armillaria* (Fr.) Staude and *Xerula* Maire/*Hymenopellis* R.H. Petersen (*Physalacriaceae* Corner) within *Marasmiineae* (Singer and Clémençon 1972; Watling and Chandra 1983; Watling 1992; Clémençon 2004; Antonín and Dvořák 2010; Petersen and Hughes 2010); *Amanita* Pers. (*Amanitaceae* E.-J. Gilbert) within *Pluteineae* Aime, Dentinger & Gaya (Kotilová-Kubičková and Pouzar 1988; Tulloss and Halling 1997); *Clavariastellifera* J. Geesink & Bas, *Camarophyllopsis* Herink s.l. and *Ramariopsis* (Donk) Corner (*Clavariaceae* Chevall.) within *Clavariineae* Olariaga, Huhtinen, Læssøe, J.H. Petersen & K. Hansen (Singer 1986; Geesink and Bas 1992; Halama et al. 2017); *Crepidotus* (Fr.) Staude (*Crepidotaceae* (S. Imai) Singer) and *Inocybe* (Fr.) Fr. s.l. (*Inocybaceae* Jülich) within *Agaricineae* Fr. (Kuyper 1986; Senn-Irlet 1995); *Calocybella* Vizzini, Consiglio & Setti (*Lyophyllaceae* Jülich), *Fayodia* Kühner (*Fayodiaceae* Jülich), *Dermoloma* J.E. Lange ex Herink (*Tricholomataceae*) and *Entoloma* (Fr.) P. Kumm. (*Entolomataceae* Kotl. & Pouzar), within *Tricholomatineae* (Singer 1986; Arnolds 1993; Horak and Desjardin 1993; Manimohan et al. 1995; Latha et al. 2020). Their presence is a generic character only for *Armillaria* and *Camarophyllopsis* s.l. (Singer 1986).

The microchemical reaction, 5% KOH pileipellis fragments which commonly become blue green is reminiscent of that exhibited by some *Gymnopus* (Pers.) Gray species allied with *G.alkalivirens* (Singer) Halling (Halling 1979, 1981, 1990; Antonín and Noordeloos 1997, 2010) (*Omphalotaceae*, *Marasmiineae*), *Xerophorus* (Bon) Vizzini, Consiglio & M. Marchetti (Vizzini et al. 2020a) (*Callistosporiaceae* Vizzini, Consiglio, M. Marchetti & P. Alvarado, *Tricholomatineae*) and *Leucoagaricus* Locq. ex Singer/*Leucocoprinus* Pat. species (*Agaricaceae*, *Leucocoprineae* Singer, Bon 1993; Vellinga et al. 2010; Asif et al. 2024; Kooij et al. 2024; Yang et al. 2024).

With the exclusion of *Pseudobaeospora* (*Tricholomataceae* subfam. *Pseudobaeosporoideae*) from the family core *Tricholomataceae* (*Tricholomataceae* subfam. *Tricholomatoideae*) the latter subfamily is thus restricted to species characterized by a mostly tricholomatoid or rarely tricholomatoid-collybioid habit (*Dennisiomyces*, *Dermoloma*), with smooth or verrucose (*Leucopaxillus*) non-dextrinoid and thin-walled basidiospores, whose walls usually react in grey or blue to Melzer’s reagent (immediately amyloid, *Albomagister* partim, Dermolomasubg.Amylospora Adamčík, *Corneriella*, *Dennisiomyces*, *Leucopaxillus*, *Porpoloma*, *Pseudoporpoloma* and *Pseudotricholoma*; latently amyloid, *Tricholoma*; see Moreau et al. 2015; Vizzini et al. 2016, 2020b, 2024; Corriol and Jargeat 2018; Sánchez-García et al. 2021; Matheny et al. 2024).

#### ﻿ Pseudobaeospora pillodii

Taxon classificationFungiAgaricalesTricholomataceae

(Quél.) Wasser, Flora Gribov Ukrainy. Agarikoyve Griby (Kiev): 220 (1980)

63233F0E-DC7C-565C-AFDB-F8ECB11180BC

Figs 4d, g , 5 , 6

Pseudobaeospora pillodii (Quél.) E. Horak, Revue Mycol., Paris 29(1–2): 73 (1964), Nom. inval., Art. 41.3 (Shenzhen Code).
Collybia pillodii Quél. [as ‘pillodi’] Basionym, C. r. Assoc. Franç. Avancem. Sci. 18(2): 509 (1890) [1889].
= Pseudobaeosporaoligophylla (Singer) Singer, Lilloa 22: 438 (1951) [1949].
Baeospora oligophylla Singer, Basionym, Revue Mycol., Paris 3(4–5): 194 (1938).
= ? Agaricus (Tricholoma) microsporus Ellis *sensu*Desjardin (2004) non *sensu*Singer (1942).

##### Lectotype of *Collybiapillodii*.

**Figure 4.**
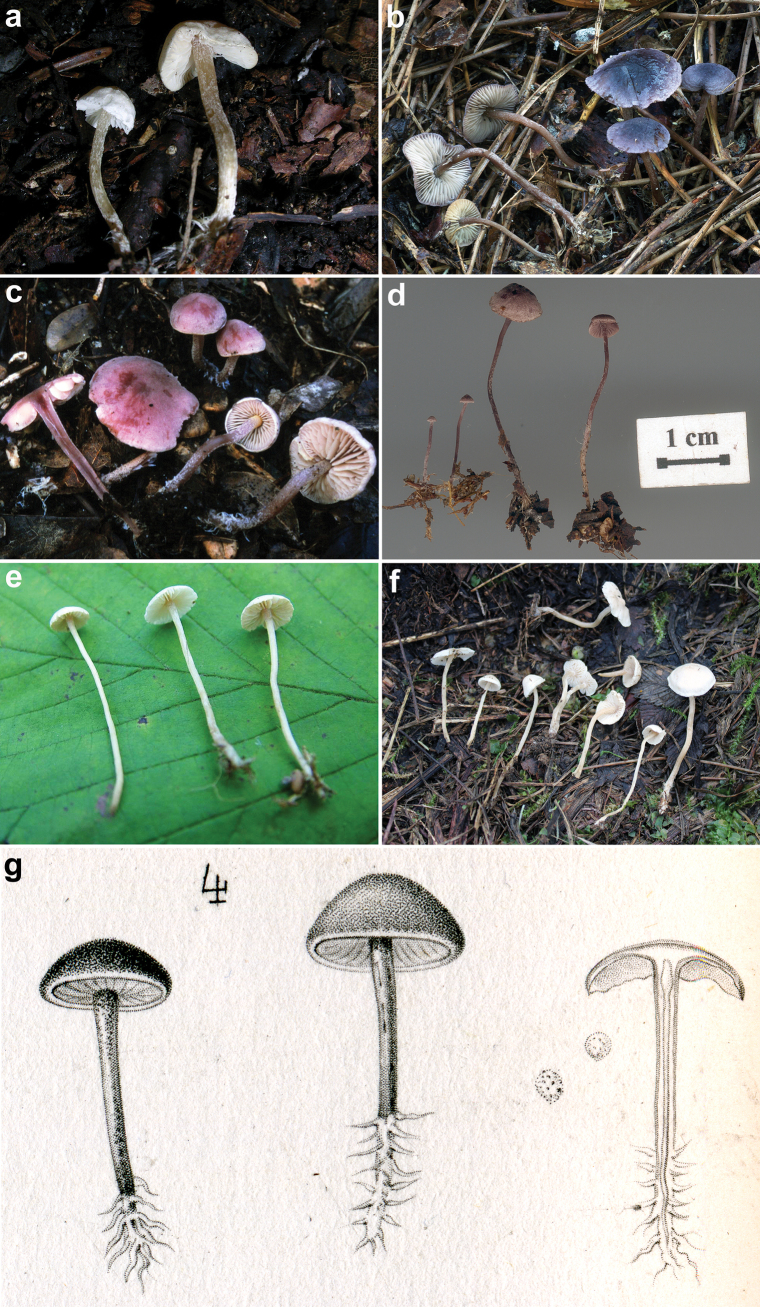
Basidiomes of some sequenced *Pseudobaeospora* collections. **a***P.calcarea* (LIP PAM06090111-FR2013078); **b***P.cyanea* (GDOR M3986); **c**P.laguncularisvar.denudata (LIP PAM99101004-FR2013079); **d***P.pillodii* (KRAM F-53314); **e***P.terrayi* (SAV-F:3317, holotype); **f***P.terrayi* (SAV-F:20813). Lectotype of. *C.pillodii*; **g** Quélet’s original plate, pl. XV, fig. 4. Photos: **a, c** by P.A. Moreau; **b** by D. Gisotti; **d** by A. Ronikier; **e, f** by S. Jančovičová.

(selected here, MBT10024681): Quélet’s original plate, 1890, C.R. Ass. franç. Av. Sci. 18: pl. XV, fig. 4. Fig. 4g

##### Epitype of *Collybiapillodii*.

(designated here, MBT10024682): FRANCE • Savoie, Bourg-Saint-Maurice, Arc 1800, under *Alnusalnobetula* (Ehrh.) K. Koch, 27 August 2006, leg. P-A Moreau (LIP PAM06082703).

##### Selected iconography.

Ludwig (2000: 152, 70.1); Ronikier and Moreau (2007: 1b, c); Morozova and Popov (2013: pl. I-1); Christan and Rexer (2020: 41).

##### Selected descriptions.

Kühner and Romagnesi (1954: 92, as *Collybiapillodii*); Horak (1968: 511–513, as *P.oligophylla*); Redhead (1982: 217, as *P.pillodii*, no data on presence/absence of clamp-connections); Bas (2003: 192–193, as *P.pillodii*, 194–195 as *P.oligophylla*); Morozova and Popov (2013: 129–130, as *P.pillodii*, in Russian).

##### Description.

**Figure 5.**
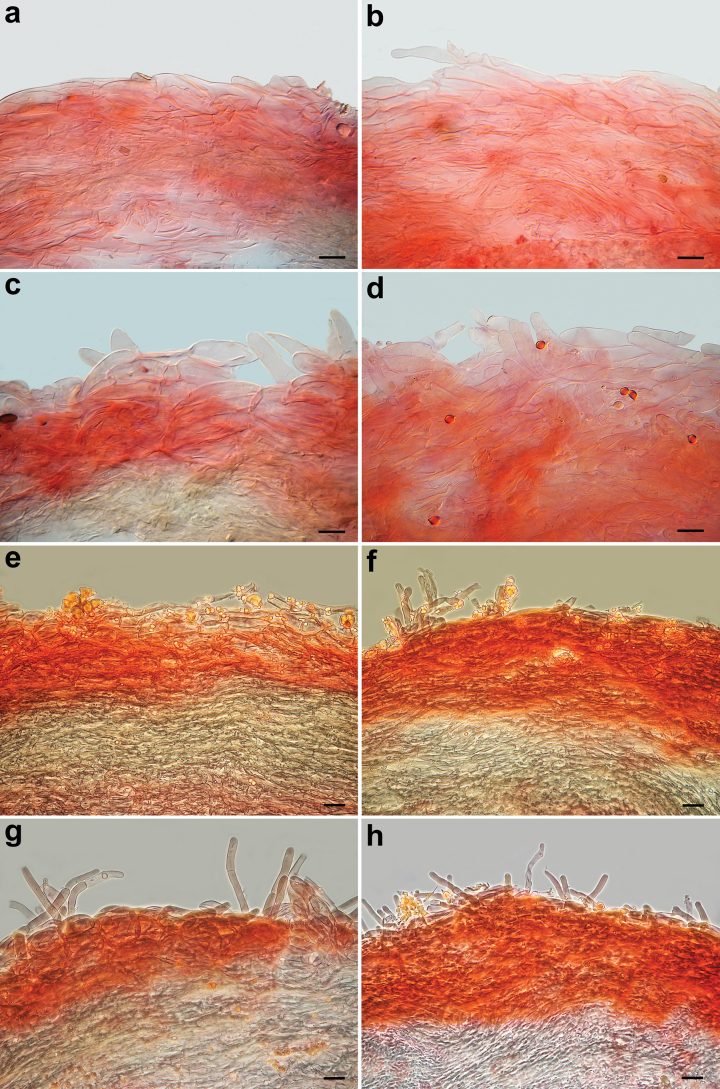
*Pseudobaeosporapillodii*, microscopic features. Pileipellis. **a–c** (KRAM F-53298); **d** (KRAM F-53314); **e, f** (LE 254346); **g, h** (O-F:258872); **a–h** in ammoniacal Congo red. Scale bars: 10 µm (**a–c**); 20 µm (**e–h**). Photos by L. Setti.

**Figure 6.**
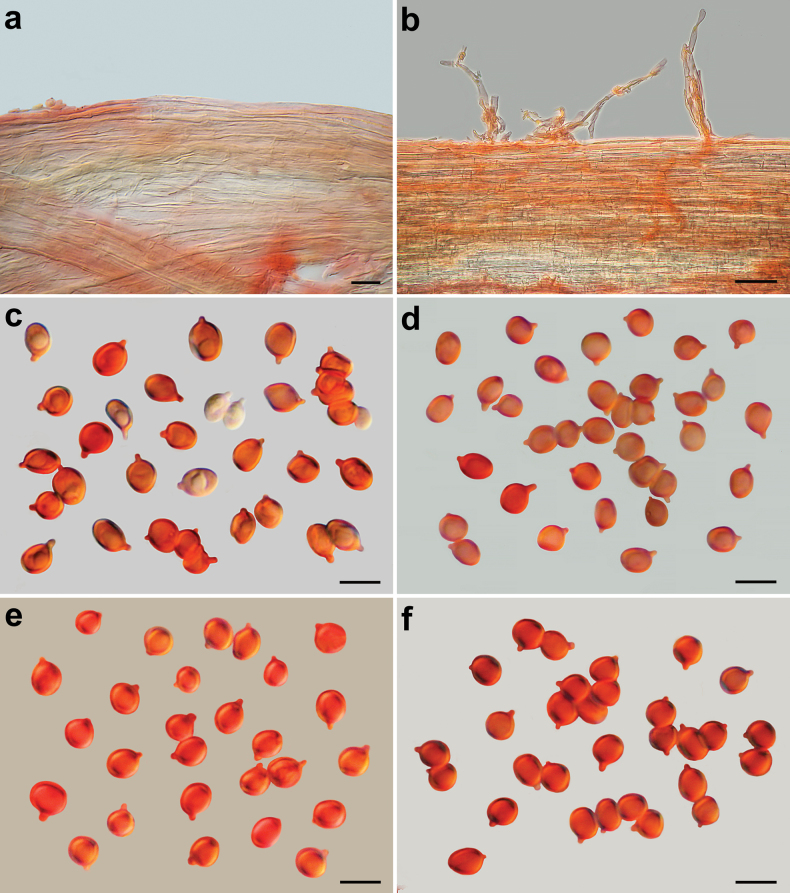
*Pseudobaeosporapillodii*, microscopic features. Stipitipellis. **a** (KRAM F-53298); **b** (KRAM F-53314). Spores; **c** (KRAM F-53298); **d** (KRAM F-53314); **e** (LE 254346); **f** (O-F:258872); **a–f** in ammoniacal Congo red. Scale bars: 10 µm (**a**); 20 µm (**b**); 5 µm (**c–f**). Photos by L. Setti.

***Spores*** (2.8–)3.3–3.7–4.2(–5.6) × (2.5–)2.9–3.1–3.4(–4.2) µm (288/5/5), Q = (0.96–)1.07–1.19–1.32(–1.69), V = (10.2–)14.6–19.8–25.0(–46.1) μm^3^, globose to subglobose or broadly ellipsoid in frontal and side view, wall up to 0.2–0.3 µm thick, smooth, colorless in L4; hilar appendix prominent, 0.5–0.8 µm long (Fig. 6c–f). ***Basidia*** 16–17.5 × 5–6 µm, mostly tetraspored but also bispored, clavate, sterigmata up to 5 µm long. ***Hymenophoral trama*** regular to subregular, consisting of up to 8 µm wide hyphae, colorless in L4. **Hymenial cystidia** absent. ***Pileipellis*** suprapellis as a quite compact (dense) cutis of broadly ellipsoid up to 16 µm wide hyphae mixed with cylindrical, up to 8 µm wide hyphae, with rounded apex, slightly gelatinized, smooth, sometimes ascendant and forming small trichodermic patches; subpellis formed by broadly ellipsoid, densely septate hyphae up to 10 µm wide (Fig. 5a–h). Pigments brownish, intracellular. ***Stipitipellis*** of cylindrical, up to 6 µm wide hyphae (Fig. 6a). ***Stipititrama*** of up to 16 µm wide hyphae. ***Caulocystidia*** usually present, clustered, versiform, thin-walled, colorless, up to 6 µm wide (Fig. 6b). ***Clamp-connections*** absent everywhere.

##### Specimen examined.

FRANCE • Savoie, Bourg-Saint-Maurice, Arc 1800, under *Alnusalnobetula* (Ehrh.) K. Koch, 27 August 2006, leg. P-A Moreau (LIP PAM06082703, epitype of *C.pillodii*). NORWAY • Innlandet, Lesja, Joris delta, Flommarkskog med gråor, 12 August 2021, leg. T.E. Brandrud, S. Khalsa & P.G. Larsen (O-F:258872). POLAND • Western Tatra Mts., Sarnia Skala massif, northern slope, at the top, alt. 1375 m, *Pinetummugicarpaticum*, on litter, 22 August 2001, leg. A. Ronikier (KRAM-F:53298); ibidem, 8 September 2001, leg. A. Ronikier (KRAM-F:53314). RUSSIA • Republic of Karachay-Cherkessia, Teberda State Nature Reserve, Dzhemagat Gorge, 1881 m, on the soil on the border of floodplain forest and meadow, 13 August 2009, leg. E.S. Popov, det. O.V. Morozova (LE 254346).

##### Notes.

*Collybiapillodii* was described and illustrated by the French mycologist Lucien Quélet (1890) from Jura hills as a very small entirely violet species with a campanulate to convex pruinose pileus, whitish pileus margin, adnate and distant lamellae paler than the pileus, a fistulous and slender rooting stipe bristling at the base with white and radiant rhizoids, very thin violaceous context, and minute subglobose to ovoid spores. He reported its spores as minutely aculeate (“finement aculeolée”), but his observation was probably incorrect because it was not confirmed by all the subsequent authors who observed always smooth spores. The species was later described in detail by Kühner and Romagnesi (1954), who suggested its possibly placement in TricholomasectionLiposperma. Favre (1960) believed that the species was better placed within the tribe Orcellées (see below) in sense of Kühner (see Kühner and Romagnesi 1953, Kühner 1969 = *Entolomataceae*).

When Singer (1938) described *Baeosporaoligophylla* he did not compare it to *Collybiapillodii* described 48 years earlier by Quélet (1890). He merely noted that the latter species probably also belongs to the genus *Baeospora* Singer. Then, when Singer (1942) established the genus *Pseudobaeospora* for the species of *Baeospora* with dextrinoid spores, he included only *B.oligophylla*. Both in the first and in second edition of “The *Agaricales* in modern taxonomy” (Singer 1951, 1962), he placed the genus *Pseudobaeospora* as closely related to *Lepiota* (following the suggestions by Locquin 1952), and still monotypic with a single species, *P.oligophylla*; while *Collybiapillodii* was classified as a probable member of CollybiasectionIocephalae Singer.

The two species were placed together in one genus for the first time by Horak (1964), who made an invalid combination *Pseudobaeosporapillodii*, validated later by Wasser (1980). In the third and fourth editions of “The *Agaricales* in modern taxonomy”, Singer (1975, 1986) also followed Horak’s opinion including the two names in one genus.

Since type collections of Quélet’s *Collybiapillodii* and of *Baeosporaoligophylla* (presumably kept in LE) do not exist (Olga Morozova, pers. comm.) and their very concise original descriptions are difficult to interpret there has been some speculation about the relation between *P.pillodii* and *P.oligophylla* and depending on the authors these taxa were treated as two independent species (Singer 1986; Bas 2002, 2003) or just one (Horak 1964, 1968, 2005; Redhead 1982; Ronikier and Moreau 2007; Morozova and Popov 2013; Desjardin et al. 2014; Voto 2021).

Bas (2003) was the only author who provided a comparison of *P.pillodii* and *P.oligophylla*. He mainly relied on his own collections, the original description of *P.oligophylla* (Singer 1938), the exhaustive descriptions of *Collybiapillodii* by Kühner (in Kühner and Romagnesi 1954) and of the Swiss specimens of *P.pillodii* by Horak (1968). He distinguished *P.oligophylla* by a cutis type of pileipellis with cystidioid, repent to ascending, terminal elements compared to a simple undifferentiated cutis of *P.pillodii*. These conclusions were later questioned by Ronikier and Moreau (2007) who, after finding and studying specimens at various stages of development, proposed to consider both taxa as synonyms, giving the priority to *P.pillodii* as an older name. They concluded that the cutis-like pileipellis observed by Bas as characteristic of *P.pillodii* is only the tardive stage of the subtrichodermic pileipellis found in young specimens named as *P.oligophylla*.

Our analyses, which did not take into account the developmental stage of the basidiomes as they were carried out on only herbarium (fungarium) material, show that regardless of the presence or absence of ascending pileocystidioid terminal elements (which in our opinion depends more on the point of the pileus where the sampling is taken) all the collections are molecularly conspecific (Figs 2, 3), and thus supporting the conclusions of Horak (1964, 1968), Redhead (1982), and Ronikier and Moreau (2007). Accordingly, Quélet’s illustration of *Collybiapillodii* (Quélet 1890) is selected above as lectotype and a sequenced French collection (LIP PAM06082703), also studied in Ronikier and Moreau (2007), is established as epitype of *C.pillodii*.

According to the results of our study, *P.pillodii* is characterized by very small to small, very slender (e.g., pileus 1–15 mm wide, stipe 10–55 (70) × 0.2–2.0 mm), brownish lilac, entirely purplish coloured basidiome except its whitish pileus margin, spaced lamellae (L = 12–19, l = 0–3), a non-striate pileus, a stipe with basal rhizoids, subglobose to broadly ellipsoid spores (on average not exceeding 4 µm in length), basidia prevailingly tetrasporic (few specimens have been found with 2-spored basidia only, e.g., Kühner and Romagnesi 1953) as well with 2- and 4- spored basidia sometimes even on one lamella (e.g., Ronikier and Moreau 2007, our observations), cheilocystidia absent (but reported as basidioliform by Horak 1964 or filiform by Singer 1938), suprapellis as a cutis with (subtrichodermoid) or without ascending terminal (pileocystidioid) elements, that is negative to pallid or turns very pale grayish-greenish in KOH, clamp-connections absent. Kühner (in Kühner and Romagnesi 1954) pointed out that all the specimens he examined were haploparthenogenic (apogamic, with uninucleate hyphae).

*Pseudobaeosporapillodii* was originally described from a deciduous mountain forest (Quélet 1890) and is often reported from *Alnus* Mill. litter (Kühner in Kühner and Romagnesi 1954; Favre 1960; Bas 2003; Ronikier and Moreau 2007; von Bonsdorff et al. 2012 etc.) as well as from subalpine coniferous litter (Singer 1938; Kühner in Kühner and Romagnesi 1954; Horak 1964, 1968; Bresinsky and Schmid-Heckel 1982; Redhead 1982; Ronikier and Moreau 2007; Læssøe 2008, 2012; Morozova and Popov 2013; Christan and Rexer 2020) or other subalpine plant communities, such as *Salix* L. shrubs, the Athyrio-Sorbetum association (Bujakiewicz 2004) or *Rhododendron* L. shrubs (Wasser 1980). As suggested by Ronikier and Moreau (2007), it might be a nitrophilic saprotrophic species, to be sought in other nitrogen-rich organic substrates. It is reported from Asia (Siberia), Europe (France, Germany, Norway, Poland, Switzerland, Russia), and North America (Canada) (Singer 1938; Horak 1964, 1968; Redhead 1982; Bas 2003; Ronikier and Moreau 2007; Læssøe 2008, 2012; Morozova and Popov 2013; Christan and Rexer 2020; Voto 2021). Jamoni (1997) described a *Pseudobaeospora* collection (on only one basidiome in very poor condition) from subalpine Piedmont (Italy) near *Alnusincana* which may represent *P.pillodii* due to its tetrasporic basidia, clampless hyphae and absence of cheilocystidia, but unfortunately the specimen can no longer be found in any fungarium.

There are several interesting observations on *P.oligophylla* or *P.pillodii* which were not confirmed by other authors and require further investigation. Favre (1960) and Kühner (1980) reported a lilac-purplish or pinkish spore deposit for *P.pillodii*. Bon (in Jamoni and Bon 1996) cited some French collections of *P.pillodii* consisting of only albinotic basidiomes, but these may represent white *Pseudobaeospora* species which were described later (e.g., Bas 2002, 2003).

For a single collection named *P.oligophylla* in sense of Bas, the collector, N. Dam, noted that some rhizoids were connected to small ochraceous tubers (presumably sclerotia) in the soil, as in *Collybiatuberosa* (Bull.) P. Kumm. (cited in Bas 2003; *Clitocybaceae* Vizzini, Consiglio & M. Marchetti, *Tricholomatineae*).

The presence of bisporic and/or tetrasporic basidia in basidiomes and/or collections of the same species found in *P.pillodii* should not be surprising as, apart from *P.wipapatiae* and *Agaricusfuscolilacinus* Peck (that according to Desjardin 2004 belongs to *Pseudobaeospora*) for which only bisporic basidiomes are known (Desjardin 2004; Desjardin et al. 2014), some other species, e.g., *P.brunnea*, *P.cyanea*, *P.lilacina*, show a mixture of (1)2- and 4-spored basidia (Bas 2003; Arauzo 2011a; Wu et al. 2017; Voto 2021), *P.pyrifera* collections with 2–4 spored basidia and others only 4-spored (see below), and for *P.taluna* three collections from Tasmania are 4-spored, and one from Victoria, 2-spored (Craig et al. 2023).

*Pseudobaeosporapillodii* was the only clamp-less species reported from Europe so far before this publication (see below). *Pseudobaeospora* sp. described in Adamčík and Ripková (2004b) based on a single clamp-less basidiome collected among *Molinia* sp. under *Alnusglutinosa* (L.) Gaertn., from Czech Republic, is distinguished mainly by a very minute basidiome (pileus 4.5 mm wide and stipe 20 × 0.6 mm), pileus with 1–1.5 mm long marginal striation, very sparse lamellae (L = 11, l = 0–1), well-developed versiform to irregular cheilocystidia 21–33 × 2.5–6.0 µm, strictly bisporic basidia, a pseudoparenchymatic subpellis of 9–24 µm wide hyphae, and spores longer than 4 µm on average.

Agaricus (Tricholoma) microsporus Ellis (Nom. illegit., Art. 53.1, Shenzhen Code) is, based on the data provided by Desjardin (2004) who examined its holotype collection (“*this species forms violet basidiomes with a thin cutis-type pileipellis that overlays a subcellular hypodermium*, *has dextrinoid basidiospores 4–5 × 3.5–4.5 µm, lacks cheilocystidia, lacks clamp connections, and does not discolor in KOH*”), and the original description (Ellis 1874, stipe with “*long, spreading, pale-yellowish hairs at base*”), a possible older synonym of *P.pillodii*.

#### ﻿ Pseudobaeospora deceptiva

Taxon classificationFungiAgaricalesTricholomataceae

Vizzini, Consiglio, Setti & Adamčík sp. nov.

94F027D8-CBAE-5003-A10A-8772CEEBE5D5

857523

Fig. 7

##### Diagnosis.

*Pseudobaeosporadeceptiva* differs from *P.pillodii* by larger basidiospores, tetrasporic basidia and lack of rhizoids and from the other members of the genus by its unique phylogenetic position.

##### Etymology.

the species epithet derives from the Latin word *deceptivus* (= misleading) and refers to its strong resemblance to *P.pillodii*.

##### Holotype (here designated).

ITALY • Abruzzo, Ponte di Platano (CH), on the ground, on rotting leaves of *Alnusalnobetula* [= *A.viridis* (Chaix) DC.], 28 August 2000, leg. et det. G. Robich, as *P.pillodii* (MCVE:15315).

##### Description.

**Figure 7.**
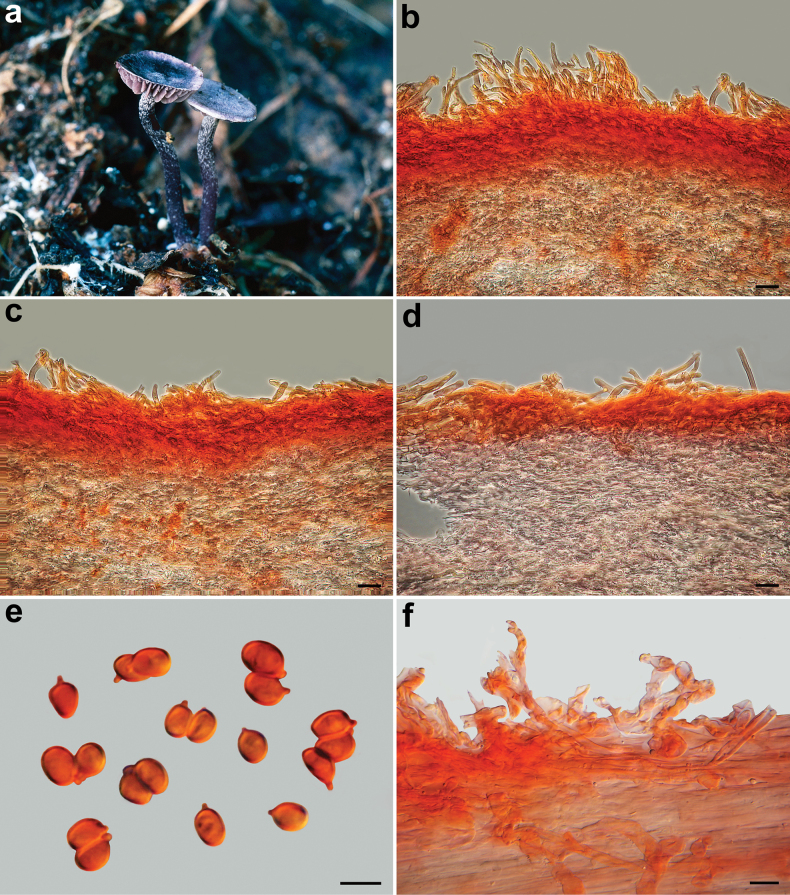
*Pseudobaeosporadeceptiva* (MCVE:15315, holotype). **a** Basidiomes; **b–d** pileipellis; **e** spores; **f** caulocystidia; **b–f** in ammoniacal Congo red. Scale bars: 20 µm (**b–d**); 5 µm (**e**); 10 µm (**f**). Photos by L. Setti.

***Habit*** collybioid. ***Pileus*** 5–20 mm broad, conical campanulate to plano-convex, expanding plane with an obtuse umbo, margin at first slightly crenulated-undulate, not striate, flattened to revolute in mature specimens, surface dry, pruinose to minutely felted, not or only slightly hygrophanous, dark violaceous (Dark Bluish Violet, Blackish Violet, Plate X; Navy Blue, Plate XXI; Dusky Violet-Blue 1, Plate XXIII), with a whitish paler margin. ***Lamellae*** deeply emarginate with slightly decurrent tooth to almost free, spaced, L = 18–20, 1 = (1–)3–5(–7), rather thick, narrow to ventricose, 1.5–2 mm broad, purple-lilaceous (Pale Amparo Purple, Light Amparo Purple, Plate XI; Light Mallow Purple, Mallow Purple, Plate XII; Pale Vinaceous, Plate XXVII), with a concolorous, entire to slightly irregular/eroded edge. ***Stipe*** 40–60 × 1.5–2.5 mm, cylindrical, flexuous, solid to slightly hollow at maturity, not filiform, concolorous with the pileus, at first sparsely but entirely covered by minute silky whitish fibrils and flocks, then evidently fibrillose only at apex (Fig. 7a), base radially strigose. ***Context*** violaceous. ***Pileus surface*** showing a negative reaction with a drop of 5% KOH. ***Smell*** indistinct. ***Taste*** mild. ***Spore-print*** white.

***Spores*** (3.9–)4.2–4.6–5.0(–5.4) × (2.6–)3.1–3.4–3.8(–4.1) µm (64/2/2), Q = (1.18–)1.25–1.36–1.46(–1.63), V = (15.4–)20.9–28.9–36.8(–46.5) μm^3^, broadly ellipsoid to ellipsoid, colorless, smooth, in L4; contents granulose or with one or two oil-droplets (guttules), at first thin-walled and inamyloid, but maturing after liberation and becoming thick-walled up to 0.8 µm, dextrinoid, congophilous and cyanophilous; hilar appendix prominent, 0.8–1 µm long (Fig. 7e). ***Basidia*** 15–19 × 6–7 µm, clavate, tetrasporic, with sterigmata up to 3 µm long. ***Hymenophoral trama*** regular to subregular, consisting of hyphae up to 8 µm wide, colorless in L4. **Hymenial cystidia** not observed. ***Pileipellis***: slightly gelatinized, a cutis composed of loose, 2.5–4 µm wide hyphae; hyphal terminations towards the pileus margin often ascending and arranged in subtrichodermic patches, subcylindrical, smooth, apically rounded, up to 8 µm wide; subpellis consisting of up to 8 µm wide cylindrical hyphae (Fig. 7b–d). Pigment brownish, intracellular. ***Stipitipellis*** consisting of 3–7 µm wide, non-dextrinoid parallel-oriented cylindrical hyphae. ***Stipititrama*** similar to stipitipellis structure but hyphae up to 10 wide. ***Caulocystidia*** present (observed at stipe apex), 10–40 × 5–10 µm, usually in tufts, thin-walled, colorless, often irregularly shaped, clavate, lageniform, sinuous, lobed, sometimes catenulated, with rounded apex (Fig. 7f). ***Clamp-connections*** absent in all parts.

##### Habitat and distribution.

Terrestrial, so far known only from Italy.

##### Additional specimen examined.

ITALY • Piemonte, Val d’Otro, Alagna Valsesia (VC), 10 September 1994, leg. et det. P.G. Jamoni, as *P.pillodii* (MCVE:4905).

##### Notes.

*Pseudobaeosporadeceptiva* is a species difficult to distinguish from *P.pillodii* without careful observation of some morphological characters: it shows larger basidiomes (pileus 5–20 mm and stipe up to 2.5 mm wide), a stipe without basal rhizoids, spores on average longer than 4.5 µm, and frequent subtrichodermic structures near the pileus margin. The two collections studied here were previously identified as *P.pillodii*. The difference between the spore dimensions of the two species is even more worthy of attention if we consider that while the collections of *P.pillodii* show a variable percentage of bisporic basidia, those of *P.deceptiva* are consistently tetrasporic. The ITS sequences do not unambiguously separate the two species (Fig. 3) but they are clearly different in the multigene analysis where also their SSU and LSU sequences are present (Fig. 2).

*Pseudobaeospora* sp. described in Adamčík and Ripková (2004b) shares with *P.deceptiva* the colours of the basidiomes, the clamp-less hyphae, spores longer than 4 µm in average and a suprapellis containing numerous ascending to erect terminal elements (pileocystidia according to Bas 2003) but is distinguished by a very minute basidiome (pileus 4.5 mm wide and stipe 20 × 0.6 mm), pileus with 1–1.5 mm long marginal striation, very sparse lamellae (L = 11, l = 0–1), well-developed versiform to irregular cheilocystidia, different length/width spore ratio (Q = 1.08–1.29), bisporic basidia, and a pseudoparenchymatic subpellis of 9–24 µm wide hyphae.

Among the extra European clamp-less species, *P.defibulata* Singer described from Argentina on rotten leaves of dicotyledonous trees differs by a smaller pileus (3–7 mm wide), a thinner stipe (0.2–0.6 mm thick), a pale livid to partly almost white pileus, sparse lamellae (L = 13, l = 1), smaller spores, 4.0–4.2 × 3.0–3.2 µm, and a suprapellis of only horizontal hyphae (Singer 1963). *Pseudobaeosporacitrina* Rawla from India, is distinguished by small basidiomes (pileus 5–10 mm, stipe 10–15 × 1 mm) greenish yellow to citrine, pileipellis a trichoderm of repent up to 7 µm wide hyphae, with fasciculate, slightly thick-walled, 28–140 × 3–5 µm hairs (pileocystidia) (Rawla and Arya 1991; Bas 2003).

#### ﻿ Pseudobaeospora pyrifera

Taxon classificationFungiAgaricalesTricholomataceae

Bas & L.G. Krieglst., Z. Mykol. 64(2): 204 (1998)

724F2A22-0243-5033-861F-CC8CDD1BC9E5

Figs 8 , 9 , 10

= Pseudobaeosporamutabilis Bas & Adamčík, in Adamčík and Bas, Mycotaxon 84: 272 (2002)

##### Holotype.

GERMANY • Bavaria, Lower Franconia, Kitzingen, ‘Klosterforst’, 10 September 1995, leg. L.G. Krieglsteiner s.n. (holotype L; isotype REG).

##### Selected iconography.

Krieglsteiner (1999: 37, photo G. Wölfel); Chaillet et al. (2007: 7, photo G. Moyne); Krieglsteiner (2010: 74); Arauzo (2011a: p. 34–36); Morozova and Popov (2013: pl. I-2); Caillet et al. (2018: 71).

##### Selected descriptions.

Bas and Krieglsteiner (1998: 204–205); Adamčík and Bas (2002: 272–274, as *P.mutabilis*); Bas (2003: 175–177); Chaillet et al. (2007: 5–7); Arauzo (2011a: 34–35); Morozova and Popov (2013: 131–132, in Russian).

##### Description.

**Figure 8.**
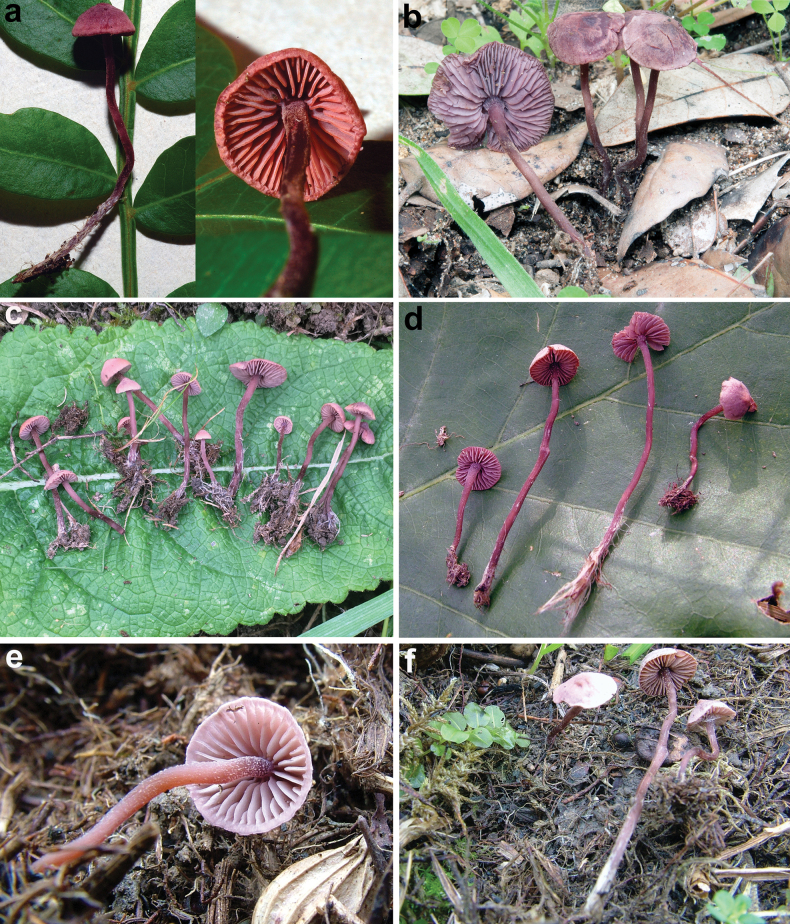
*Pseudobaeosporapyrifera*. Basidiomes. **a** AMB 18729; **b** AMB 18730; **c** SAV-F:3518 (holotype of *P.mutabilis*); **d–f** SAV-F:3524. Photos: **a, b** by G. Consiglio; **c–f** by S. Jančovičová.

***Habit*** collybioid. ***Pileus*** 8–26 mm broad, conical campanulate to plano-convex, finally flattened with an obtuse umbo, margin at first slightly crenulated-undulate, not striate, flattened to revolute in mature specimens, surface dry, pruinose to minutely felted, not or only slightly hygrophanous, purplish brown (Pinkish Vinaceous, Deep Vinaceous, Plate XXVII), dark vinaceous brown to pinkish brown at centre (Dull Magenta Purple, Schoenfeld’s Purple, Indian Lake, Plate XXVI) with a paler margin (brownish pink, Pale Vinaceous, Plate XXVII). ***Lamellae*** deeply emarginate with slightly decurrent tooth to almost free, moderately crowded, L = 18–23, 1 = (1–)2–5, rather thick, narrow to ventricose, 2–3 mm broad, reddish violaceous to violaceous pink, becoming lilacinous ochraceous (Lilac, Plate XXV; Pale Rose-Purple, Rosolane Pink, Plate XXVI) to greyish ochraceous, with a concolorous, entire to slightly irregular edge. ***Stipe*** 25–50 × 1.2–3.0 mm, cylindrical, solid to slightly hollow at maturity, concolorous with lamellae, dark vinaceous red-brown, purplish brown, at first sparsely but entirely covered by minute silky whitish fibrils and flocks, then evidently fibrillose only at apex, with long whitish strigose felt hair at the base (Fig. 8a, c, d). ***Context*** violaceous red, darkening when bruised. Pileus surface showing a bluish-green reaction with a drop of 5% KOH. ***Smell*** indistinct. ***Taste*** mild. ***Spore-print*** white.

**Figure 9.**
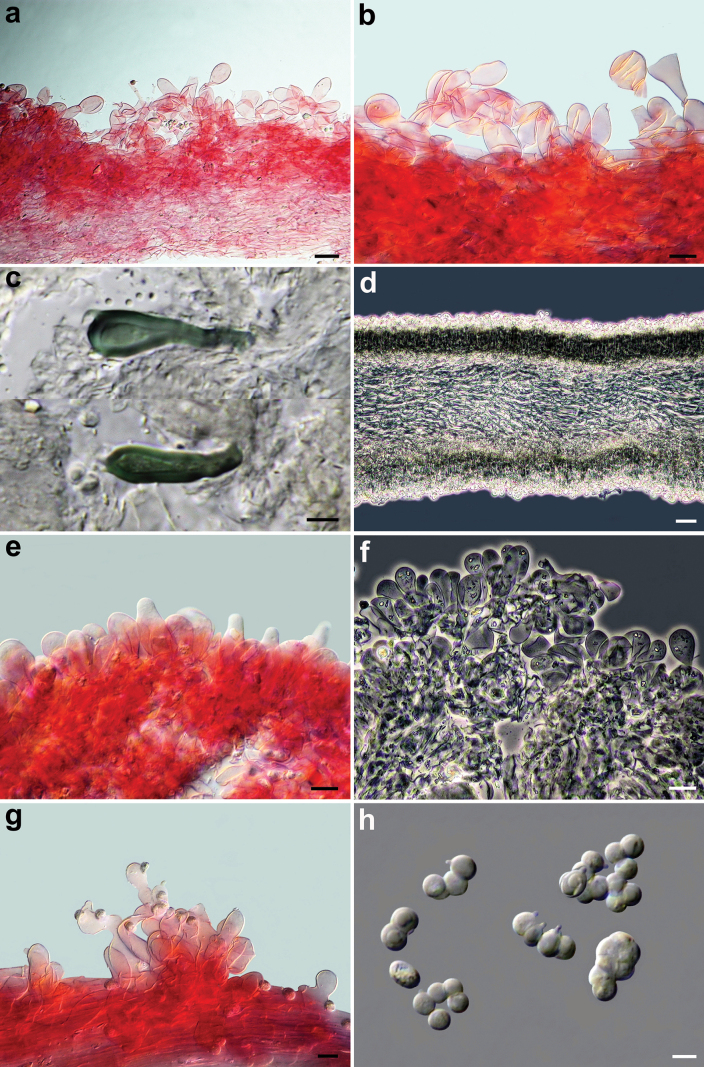
*Pseudobaeosporapyrifera*, microscopic features (AMB 18730). **a, b** Pileipellis; **c** crassobasidia; **d** hymenophoral trama; **e, f** cheilocystidia; **g** caulocystidia; **h** spores; **a, b, e, g** in ammoniacal Congo red; **c, d, f, h** in L4. Scale bars: 20 µm (**a, d**); 5 µm (**b, c, e–h**). Photos by L. Setti.

**Figure 10.**
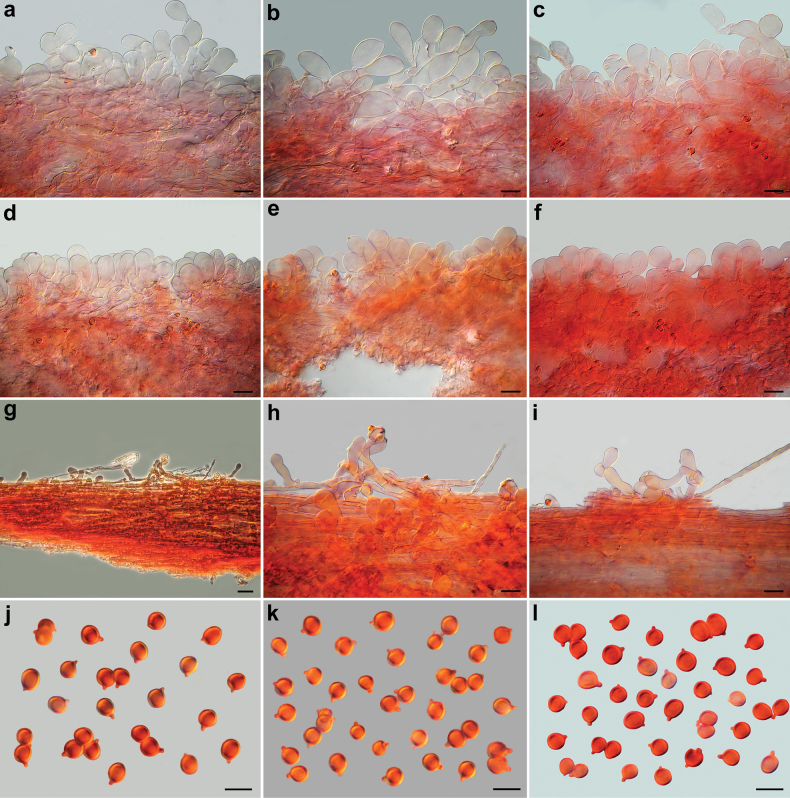
*Pseudobaeosporapyrifera*, microscopic features (collections named as *P.mutabilis*). Pileipellis. **a** SAV-F:20580; **b** SAV-F:20589; **c** SAV-F:3524. Cheilocystidia; **d** SAV-F:20580; **e** SAV-F:20589; **f** SAV-F:3524. Caulocystidia; **g** SAV-F:20580; **h** SAV-F:20589; **i** SAV-F:3524. Spores; **j** SAV-F:20580; **k** SAV-F:20589. **l** SAV-F:3524; **a–l** in ammoniacal Congo red. Scale bars: 10 µm (**a–f, h–i**); 20 µm (**g**). **j–l**. Photos by L. Setti.

***Spores*** (Italian collections, mono-, bi- and tetrasporic) (3.5–)3.8–4.3–4.7(–5.8) × (2.5–)3.0–3.5–4.0(–5.0) μm (64/2/2), Q = (1.00–)1.10–1.22–1.35(–1.63), V = (13.5–)18.3–28.6–38.9(–73.8) μm^3^, (Slovak collections, tetrasporic) (2.9–)3.2–3.5–3.8(–4.7) × (2.4–)2.9–3.1–3.3(–3.9) μm (160/3/3), Q = (0.96–)1.04–1.12–1.20(–1.36), V = (10.0–)14.2–17.8–21.3(–31.6) μm^3^, globose, subglobose to subelliptical, sometimes larmiform (drop-like), colorless, smooth, with the tendency to agglomerate in tetrads or in greater numbers (clusters) (Figs 9h, 10j–l), contents granulose or with one or two oil-droplets (guttules), at first thin-walled and inamyloid, but maturing after liberation and becoming thick-walled (0.7–1.3 µm thick), dextrinoid, congophilous and cyanophilous; majority of spores (80–90%) turns green-blue sea in L4 (Fig. 9h). ***Basidia*** 21–25 × 5.2–7.2 µm, clavate, sometimes constricted in the middle, in some collections mostly tetrasporic, but also 1–2 sporic, in others strictly tetrasporic, sterigmata up to 4 µm long. Crassobasidia (sclerobasidia) scattered, with thick dextrinoid, strongly congophilous walls (0.8–1 µm thick) turning green-blue sea in L4 (Fig. 9c). ***Hymenophoral trama*** subregular to irregular, slightly intertwined (intricate), consisting of hyphae up to 14 µm wide (Fig. 9d). ***Cheilocystidia*** 15–25 × 5.8–9.7 × 2.4–5.0 µm, abundant and densely packed, mostly broadly clavate to sphaeropedunculate, sometimes lageniform, subutriform, thin-walled, colorless (Figs 9e–f, 10d–f). ***Pleurocystidia*** absent. ***Pileipellis***: suprapellis consisting of loosely arranged chains of inflated pyriform to broadly clavate elements (transition between trichoderm and epithelium) up to 15 µm wide (Figs 9a, b, 10a–c), overlying on a subpellis made up of radially to irregularly arranged chains of largely ellipsoid to cylindrical hyphae, 7–10 µm wide, with minutely yellow-brown encrusting extracellular pigments and intracellular pigments which turn pale greenish blue in KOH (dried material). ***Pileitrama*** made up of non-dextrinoid cylindrical hyphae up to 10 µm wide. ***Stipitipellis*** consisting of 2–7 µm wide, non-dextrinoid cylindrical hyphae. ***Stipititrama*** similar to stipitipellis but hyphae up to 11 wide. ***Caulocystidia*** present (observed at stipe apex), 10–30 × 6.7–11 µm, usually clustered in tufts, thin-walled, colorless, narrowly pyriform, clavate, lageniform, sometimes irregularly shaped, often catenulated, apically rounded (Figs 9g, 10g–i). ***Clamp-connections*** present.

##### Habitat and distribution.

Terrestrial, single or in small groups (gregarious), rarely subfasciculate. Known from France, Germany, Italy (the present study), The Netherlands, Spain, Norway, Estonia, and Sweden (see collections in Fig. 3) and Slovakia (as *P.mutabilis*).

##### Specimen examined.

ITALY • 11 December 2009, Punta Ala (Castiglione della Pescaia, GR), in a mixed forest of *Quercusilex* L. and Juniperusoxycedrusssp.macrocarpa (Sm.) Neilr., leg. L. Setti (AMB 18729); 23 November 2016, Castelporziano (Ostia, RM), in a mixed forest of broad-leaved trees, leg. G. Consiglio & A. Gennari (AMB 18730). SWEDEN • Dalarna, 4 September 2018, leg. E. Larsson (GB:0160633) (as *P.pillodii*). SLOVAKIA • Záhorská níñina lowland, Abrod National Nature Reserve, Závod village, among the tall vegetation of *Moliniacaerulea* (L.) Moench, near solitary *Betula* and *Frangulaalnus* Mill., 12 August 1998, leg. S. Adamčík, V. Stanová & A. Viceníková (SAV-F:3518, holotype of *P.mutabilis*); • Biele Karpaty Mts., 1.5 km E of Nová Bošáca, Blažejová Nature Monument, on soil among the grass, 30 July 2005, leg. S. Adamčík (SAV-F:3525, as *P.mutabilis*); • ibidem, 27 September 2005, leg. V. Kučera, (SAV-F:3524, as *P.mutabilis*); • Biele Karpaty Mts., Blažejová Nature Monument, Nová Bošáca village, the settlement of Španie, 29 June 2020, leg. S. Adamčík (SAV-F:20580, as *P.mutabilis*); • Biele Karpaty Mts., Krivoklátske lúky, 20 July 2020, leg. S. Adamčík (SAV-F:20589, as *P.mutabilis*).

##### Notes.

*Pseudobaeosporapyrifera* was originally described from Southern Germany (Bavaria, Lower Franconia) and The Netherlands (Bas and Krieglsteiner 1998). Krieglsteiner (1999) reported five additional collections from the type locality (Lower Franconia) and seven collections from three other localities. At the type locality he found it sometimes to be the most abundant fungal species. It has been found in moist woods (*Pruno*-*Fraxinetum*), grasslands (*Cirsiotuberosi*-*Molinietum*) and a *Juniperus* stand, often together with *Hygrocybe*, *Entoloma*, *Geoglossum* and *Ramariopsis* species. The species has been later reported also from Spain, Iurreta (Bizkaia), locality of the Basque Country (N Spain), in the litter of *Chamaecyparislawsoniana* (A. Murray) Parl. plantations (Arauzo 2011a), from France (Haute-Saône and Doubs) in dry lawns (Chaillet et al. 2007; Caillet et al. 2018) and North-western Russia (Novgorod oblast, Batetsky district) in a meadow (Morozova and Popov 2013). *Pseudobaeosporapyrifera* was included in the CHEGD fungi (the acronym of the constituent taxa: *Clavariaceae*, *Hygrophoraceae* Lotsy, *Entolomataceae*, *Geoglossaceae* Corda and *Dermoloma*) by Caboň et al. (2021). CHEGD fungi are a particular group of macrofungi which is characteristic of traditionally managed and undisturbed European grasslands, and which are often the dominant soil fungi in these habitats.

*Pseudobaeosporamutabilis* Bas & Adamčík from Slovakia was said to have many characters in common with *P.pyrifera* Bas & L.G. Krieglst. (Adamčík and Bas 2002; Bas 2003; Adamčík et al. 2007) but distinguished by a pileipellis structure as a transition between hymeniderm and epithelium. Moreover, its basidiomes seem to be somewhat sturdier (pileus 7–13 mm in diameter, stipe 20–30 × 1–2 mm) and the lamellae less crowded (L = 18, 1 = 1–3), KOH reaction very variable (*inde nomen*), on fresh material grey, on dry specimens pale green but sometimes at first violaceous. All the here sequenced collections of *P.mutabilis* (holotype included, SAV-F:3518) form a highly supported clade together with those of *P.pyrifera* [isotype included, 10-IX-1995, L. Krieglsteiner (REG)] both in the ITS (Fig. 3) and multigene analyses (Fig. 2). Accordingly, *P.mutabilis* is here considered as a younger synonym of *P.pyrifera*. Bas (2003) had even inserted the two species, which we now consider synonymous, into two different groups of his intrageneric sectioning: *P.mutabilis* in the Celluloderma group (basidiome coloured, clamp-connections present, cheilocystidia absent or present, pileipellis hymenidermoid) together with *P.celluloderma*; *P.pyrifera* in the Pyrifera group (basidiome coloured, clamp-connections present, cheilocystidia present and conspicuous, pileipellis not hymenidermoid) together with *P.jamonii* and *P.laguncularis*. Voto (2021), in first editions of his online keys to *Pseudobaeospora*, included *P.mutabilis* in his sect.Anistoderma (pileipellis hymeniform to ephitelioid) and *P.pyrifera* in sect. Pseudobaeosporasubsect.Pseudobaeospora (pileipellis of principally short and inflated to broad hyphae). This must make us reflect on the fact that in the past too much importance has been given to the structure of the pileipellis both as a character to delimit intrageneric taxa and to distinguish species. The discrepancies in observations of the pileipellis structure might be sometimes a result of analysis of the basidiomes in different stages of development or are due to observations from different parts of the pileus (Adamčík et al. 2007; Ronikier and Moreau 2007).

*Pseudobaeosporapyrifera* is easily identifiable due to a unique combination of characters: violaceous pink tinges present all over the basidiome, the pale green to greenish blue reaction of pileus surface in KOH, mainly clavate cheilocystidia, pileipellis consisting of repent chains of inflated elements, and very small subglobose spores (Bas and Krieglsteiner 1998; Bas 2003; Arauzo 2011a). It contains peculiar metabolites named pyriferines A–C, which have an unusual eight-membered N/O-acetal ring, derived from L-glutamic acid (Quang et al. 2008). Adamčík et al. (2007) noted in some habitats the occurrence of collections with reddish brown basidiomes (see also our Fig. 8a, collection AMB 18729), instead of purplish violaceous ones, and they demonstrated that the colour of basidiomes was not affected by soil pH. Colour change to bluish grey on surfaces of basidiomes reported in original description of *P.mutabilis* (Adamčík and Bas 2002) was later not confirmed as a consistent character and might be due to local context pigment degradation rather than oxidation (Adamčík et al. 2007).

Macro- and micromorphology of the Italian collections are fully consistent with the original description (Bas and Krieglsteiner 1998; Bas 2003) and French (Chaillet et al. 2007), Spanish (Arauzo 2011a) and Russian (Morozova and Popov 2013) collections, excepted for the spores that are slightly longer [3.8–4.7 × 3.0–4.0 µm (on average 4.3 × 3.5 µm) vs 2.8–3.7(–4.2) × 2.6–3.5(–3.8) µm, vs 3.5–4.0 × 4.0–4.5 µm, vs 3.1–4 × (2.8–) 3.1–3.6 μm (on average 3.58 × 3.25 µm), and vs 2.9–3.7 × 2.6–3.2 µm, respectively] and subglobose to broadly ellipsoid instead of globose to subglobose (Qm = 1.22 vs 1.05–1.10, and 1.1, respectively). These sporal size discrepancies can be explained by the possible different percentage of (mono-)bisporic basidia on tetrasporic ones between the different collections. In fact, we report below the spore measurements summing the Slovak collections (“*P.mutabilis*”, which are exclusively tetrasporic) and Italian collections (which are mono-bi-tetrasporic): *P.pyrifera+P.mutabilis* – ***spores*** (2.9–)3.2–3.7–4.2(–5.5) × (2.4–)2.9–3.2–3.6(–4.7) μm (224/5/5), Q = (0.96–)1.05–1.15–1.26(–1.63), V = (10.0–)12.9–20.9–28.8(–65.2) μm^3^.

In this way the spore values ​​are very close to those reported in the literature. It is also worth to mention, that part of the discrepancies in published microscopic descriptions are due to an underestimation of the importance of the number of spores produced on the basidia.

In Europe *P.jamonii* Bas, Lalli & Lonati from Italy (Piedmont, Monte Rosa 1300 m, and Abruzzo) (Jamoni and Bon 1996; Bas et al. 2002; Bas 2003) seems to be the macromorphologically closest relative of the present species. It differs, however, by the more elongate clavate to (sub)lageniform, cylindrical or irregularly shaped cheilocystidia (15–43 × 4–10 μm), the presence of a distinct suprapellis of comparatively narrow hyphae, slightly larger and slightly more ellipsoid spores (3.2–4.0 × 2.8–3.5 μm, average Q = 1.10–1.15), and a different KOH reaction of the context of the stipe (green).

*Pseudobaeosporabasii* Adamčík & Ripková from Slovakia has a very similar microscopic structure but its basidiomes lack any purple or lilac tinges (Adamčík and Ripková 2004a). Unfortunately, attempts to sequence its holotype collection (SAV-F:3506) did not lead to obtaining a good ITS sequence to compare to.

*Pseudobaeosporadichroa* Bas (reported from England and Spain) has a pileus surface turning reddish purplish in KOH; pileipellis of often erect, catenulate hyphae near the centre, sometimes with a thin suprapellis, and towards margin of broad repent hyphae. L = 16–30, l = 3–5; pileus and lamellae with shades of red when dried; spores 3.0–4.0 (–4.3) × 2.7–3.5 µm, average Q 1.10–1.20 (1.25); cheilocystidia 10–45 × 3.5–10 (–17) µm, (often irregularly) clavate to lageniform, sometimes filiform, abundant to sparse or absent, sometimes with violaceous content (Bas 2003; Arauzo 2011a).

*Pseudobaeosporalaguncularis* Bas (reported from England, France, Germany, Spain) is very well characterized by the abundant, very slender cheilocystidia, at present unique in the genus. So far it is also the only species with small, scattered, refractive bodies turning red or red brown in KOH on caulocystidia and cheilocystidia, and sometimes also on the pileipellis, and with distinct, albeit sometimes sparse pileocystidia (Bas 2003; Arauzo 2011a; Clesse 2012). Pseudobaeosporalaguncularisvar.denudata Bas differs from the type by lacking the suprapellis of narrow hyphae. The collection of P.laguncularisvar.denudata here sequenced, coming from the same locality of the type (topotype) [LIP PAM99101004, France, Savoie, Billième, forêt de Lierre, sous *Buxussempervirens* L. et *Quercuspubescens* Willd. sur calcaire, alt. 400 m 10 octobre 1999, leg. Maurice Durand & Pierre-Arthur Moreau, 45.708663, 5.810281, Fig. 4c] is molecularly distinct from all the other sequenced species (Figs 2, 3).

Among the non-European species, *P.chilensis* E. Horak from Chile differs by tiny, very slender basidiomes, inconspicuous, narrow cheilocystidia, and narrower pileipellis elements (Horak 1964).

*Pseudobaeosporawipapatiae* from Hawaii, differs in forming deep ruby-colored basidiomes, with a pileus rugulo-striatulate nearly to disk, two-spored basidia, irregularly cylindrical to clavate or irregular in outline, sometimes mucronate, amyloid cheilocystidia, an irregular hymeniderm pileipellis with abundant erect pileocystidia and tissues that initially turn deep ruby then change to lilac grey in 3% KOH (Desjardin et al. 2014).

*Agaricusfuscolilacinus* Peck from Adirondack Mts. of New York (USA), based on the analysis on type material made by Desjardin (2004), who considers the species to belong to *Pseudobaeospora*, is distinguished by brownish lamellae, ellipsoid to lacrymoid bigger spores, 4.2–6.8 × 3.2–4.0 µm (on average 5.2 ± 0.6 × 3.5 ± 0.2), Q = 1.2–2.0 (Qm = 1.5 ± 0.2), two-spored basidia, and lack of cheilocystidia.

#### ﻿ Pseudobaeospora jamonii

Taxon classificationFungiAgaricalesTricholomataceae

Bas, Lalli & Lonati, Micol. Veg. Medit. 17(1): 32 (2002)

BE7011F5-D9CC-5843-AB95-6F6FC3E3ABBA

Fig. 11

– P.pillodii (forma) auct. non (Quél.) Wasser: Jamoni & Bon, Bull, trimest. Feder. mycol. Dauphine-Savoie 143: 12 (1996).

##### Holotype.

(missing): ITALY • Piemonte, Monte Rosa, Alagna, bassa valle dell’Orto, about 1300 m, 3 September 1994, leg. P.G. Jamoni (Fungarium Jamoni).

##### Neotype.

(designated here, MBT10024683): Italy, Abruzzo, San Pietro, Isola del Gran Sasso (TE), 13 September 1995, leg. G. Lalli & G. Lonati (AQUI:10322).

##### Selected iconography.

Jamoni and Bon (1996: photo on cover, as form of *P.pillodii*), Bas et al. (2002: Fig. 1).

##### Selected descriptions.

Jamoni and Bon (1996: 12–13), Bas (2003: 177–179), Bas et al. (2002: 32–35).

##### Description.

***Spores*** (2.9–)3.2–3.5–3.8(–4.5) × (2.6–)2.9–3.1–3.3(–3.6) μm (64/1/1), Q = (0.97–)1.04–1.15–1.25(–1.52), V = (11.3–)14.4–17.8–21.2(–28.2) μm^3^, globose to subglobose, wall up to a 0.8 µm thick, smooth, colorless in L4: hilar appendix prominent, 0.8–1 µm long. ***Basidia*** 16–18 × 4.0–5.0 µm, tetrasporic, subclavate to cylindrical, sterigmata up to 4 µm long; crassobasidia very rare. ***Hymenophoral trama*** regular to subregular, consisting of up to 8 µm wide hyphae, colorless in L4. ***Cheilocystidia*** mainly clavate, hardly differentiated from the basidia, but also elongate clavate to (sub)lageniform, cylindrical or irregularly shaped, 25–32 × 8.3–10.3 µm. ***Pleurocistidia*** absent. ***Pileipellis***: turning greenish brownish to pale green in KOH; suprapellis formed by broadly ellipsoid to broadly cylindrical elements, often emerging and then pileocystidia-like, sometimes slightly swollen, with rounded apex, smooth, up to 9 µm wide; subpellis slightly aeriferous, consisting of slightly gelatinized, smooth, subglobose to broadly cylindrical, up to 16 µm wide hyphae; pigments light brown, mainly intracellular. ***Stipitipellis*** composed of cylindrical, densely septate, up to 3 µm wide, longitudinal and parallel hyphae. ***Stipititrama*** of up to 10–12 µm wide hyphae, greenish in KOH. ***Caulocystidia*** at stipe apex scattered or clustered, 10–50 × 4–10 pm, filiform to narrowly clavate, subcylindrical or slender and somewhat irregular. ***Clamp-connections*** present mainly on the suprapellis hyphae and at the basidia and cheilocystidia bases.

##### Material examined.

ITALY • Abruzzo, San Pietro, Isola del Gran Sasso (TE), 13 Sept. 1995, G. Lalli & G. Lonati (AQUI:10322, neotype).

##### Notes.

Both the holotype (private fungarium, Jamoni) and the isotype collections (L) are missing (Pier Giovanni Jamoni, pers. comm. and Nicolien Sol, Leiden, pers. comm., respectively). The Italian collection studied here (AQUI:10322) was included as part of studied material (paratype) in the protologue (Bas et al. 2002) and is selected as the neotype here.

This taxon was first time provisionally described as a peculiar form of *P.pillodii* with clamp-connections, cheilocystidia and tetrasporic basidia, from Alpine areas in Piedmont (northwestern Italy) in mixed forests (*Alnusincana*, *Acer*, *Fraxinus*, *Fagus*, *Corylus*, and *Piceaabies*) often near *Buxus* (Jamoni and Bon 1996). It was then formally described as a new species (Bas et al. 2002) based on the previously cited Piedmont collections and a new collection from Abruzzi (Central Italy) and included by Bas (2003) in his European monographic work on the genus.

Both in the multigene (Fig. 2) and ITS analyses (Fig. 3), the neotype collection of *P.jamonii* is recovered as an independent species. In the multigene analysis, it is sister to a clade formed by *P.wipapatiae* and *P.pyrifera* (including *P.mutabilis*); in the ITS analysis, it is sister to a clade consisting of four environmental sequences of an undescribed probably new species from Portugal. Similarities and differences between this species and *P.pyrifera* are discussed above.

**Figure 11.**
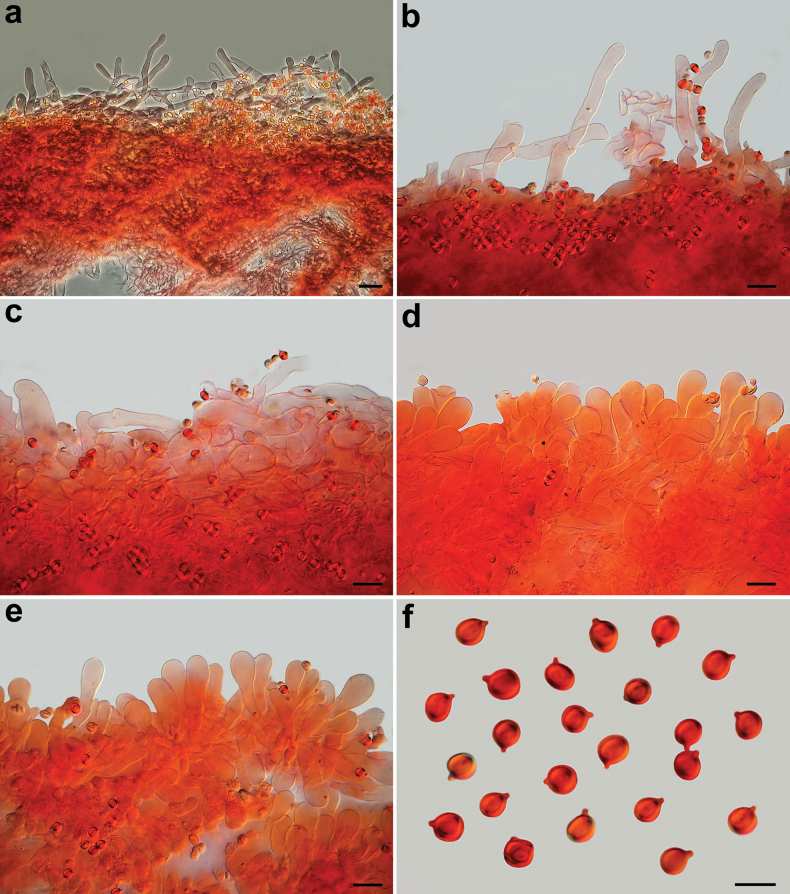
*Pseudobaeosporajamonii*, microscopic features (neotype, AQUI:10322). **a–c** Pileipellis; **d, e** cheilocystidia; **f** spores; **a–f** in ammoniacal Congo red. Scale bars: 20 µm (**a**); 10 µm (**b–e**); 5 µm (**f**). Photos by L. Setti.

#### ﻿ Pseudobaeospora celluloderma

Taxon classificationFungiAgaricalesTricholomataceae

Bas, Persoonia 18(1): 119 (2002)

4EADA132-71FD-513D-81C8-C0E91B0891C2

Fig. 12

##### Holotype.

ENGLAND • Surrey, Mickleham Downs, 19 June 1991, leg. A. Henrici (K(M) 17188).

##### Selected iconography.

Karasch (2004: 34 Abb. 6).

##### Selected descriptions.

Bas (2003: 173–174), Adamčík et al. (2007: 370–372).

##### Description.

***Spores*** (3.3–)3.7–4.0–4.4(–5.0) × (2.7–)2.9–3.2–3.5(–4.4) μm (64/2/2), Q = (1.00–)1.16–1.28–1.41(–1.61), V = (14.1–)15.9–21.5–27.2(–50.1) μm^3^, globose to subglobose or broadly ellipsoid, walls up to 0.8 µm thick, dextrinoid at maturity, smooth, colorless in L4; hilar appendix prominent, 0.8–1 µm long. ***Basidia*** 16 × 4 µm, tetrasporic, clavate, sterigmata up to 2.5 µm long. ***Hymenophoral trama*** regular to subregular, composed of globose to subglobose, broadly cylindrical, up to 22 µm wide hyphae, colorless in L4. ***Cheilocystidia*** 23–25 × 5–7 µm, thin-walled, poorly differentiated and similar to basidioles. ***Pleurocystidia*** absent. ***Pileipellis***: suprapellis cellulodermic/epithelioid, composed of slightly gelatinized, smooth, globose to subglobose, up to 24 × 22 µm elements; subpellis like suprapellis structure but with 21 × 13 µm elements, with rare cylindraceous hyphae; pigments greenish, intracellular. ***Stipitipellis*** consisting of up to 4 µm wide, multiseptate, closely packed, longitudinal hyphae. ***Stipititrama*** like stipitipellis structure but with up to 12 µm wide hyphae. ***Caulocystidia*** 12–22 × 2–7 µm, numerous at stipe apex, single or in small clusters, scattered towards the stipe base, often multiseptate with terminal elements cylindrical to broadly clavate. ***Clamp-connections*** rare, scattered.

Specimen examined: SLOVAKIA, Vihorlatské vrchy Mts., ca. 1.5 km SW of the church, old pastures, Strihovce village, terrestrial, on flysh, 18 April 2003, leg. V. Kučera (SAV-F:3516). SWEDEN, Medelpad, Borgsjö, öster om Östby, Örtrik granskog (Medelpad, Borgsjö, east of Östby, Örtrik spruce forest), 3 September 1991, leg. L. Andersson (UPS-F: 623041) (as *Pseudobaeosporapillodii*).

##### Notes.

*Pseudobaeosporacelluloderma* is a well characterized species circumscribed by its minute and slender mycenoid basidiomes which are brightly purple to reddish violet when moist (reminding *Laccariaamethystina* Cooke), sometimes greyish vinaceous coloured; strongly translucently striate pileus when moist; lamellae concolorous with pileus; pileus surface in KOH pale brownish to pale pinkish-greyish, clamp-connections present (sometimes as pseudoclamps, Bas 2003); cheilocystidia absent or basidiole-like, and an irregular hymenidermic pileipellis (Bas 2002, 2003; Adamčík et al. 2007; Kalinina et al. 2020). It was reported in Europe from Austria, Denmark, England, Finland, France, Germany, Slovakia, Sweden, and Russia (Bas 2002, 2003; Karasch 2004; Courtecuisse and Lécuru 2006; Adamčík et al. 2007; Læssøe 2008, 2012; Hausknecht et al. 2012; Kalinina et al. 2020).

The sequences from two North American collections named *P.celluloderma* (KU058501, USA North Carolina, ECV5550/TENN 067659) and *Pseudobaeospora* sp. (KU058500, USA, Tennessee TENN 070699/CCB143666) (Figs 2, 3) are weakly supported as sisters related to the two European collections we have sequenced and studied, and probably represent one or two distinct undescribed taxa.

**Figure 12.**
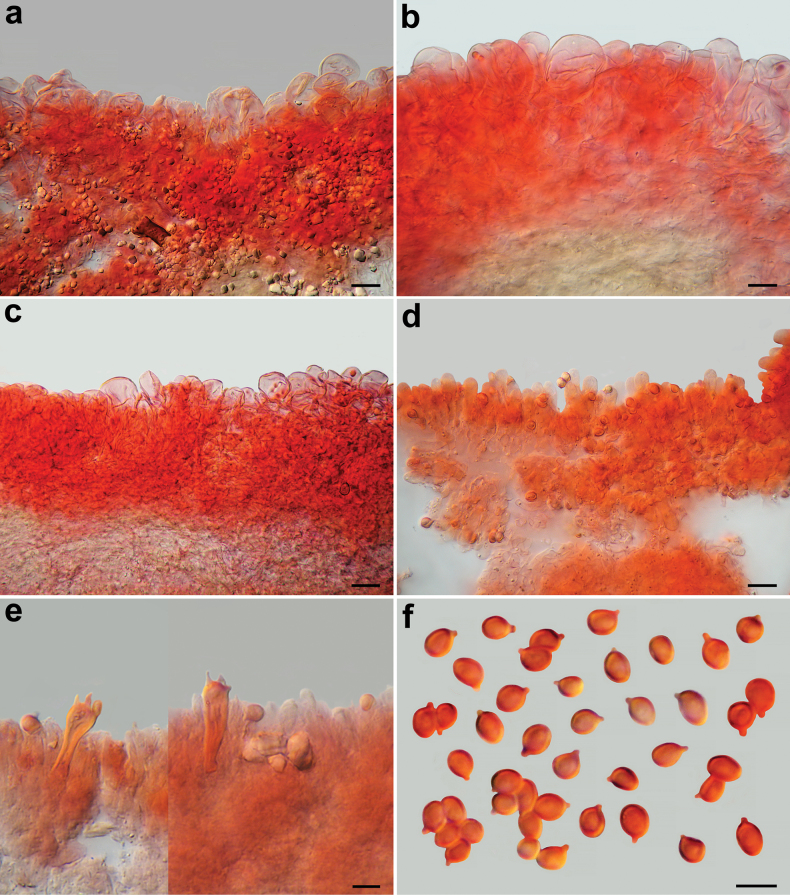
*Pseudobaeosporacelluloderma*, microscopic features (UPS-F 623041). **a–c** Pileipellis; **d** cheilocystidia; **e** crassobasidia; **f** spores; **a–f** in ammoniacal Congo red. Scale bars: 10 µm (**a, b, d**); 15 µm (**c**); 5 µm (**e, f**). Photos by L. Setti.

#### ﻿ Pseudobaeospora cyanea

Taxon classificationFungiAgaricalesTricholomataceae

Arnolds, Tabarés & Rocabruna, Revta Catal. Micol. 25: 66 (2003)

2177C112-6A44-5BC3-BCEE-F07B8830817C

Figs 4b , 13

##### Holotype.

SPAIN • Catalonia, Girona, La Selva, surroundings of Mas de Llagostera (UTM 31 T 0480 4622), 200 m, on acidic, humus-rich soil above granite, in forest with *Pinuspinaster*, *Quercussuber*, *Arbutusunedo* and *Ericaarborea*, probably saprobic, 6 November 2002, leg. J. Carreras & M. Tabarés, (BCN SCM B-4742).

##### Selected iconography.

Arnolds et al. (2003: 69), Arauzo (2011a: 24, 26), Gisotti et al. (2021: fig. 1).

##### Selected descriptions.

Arnolds et al. (2003: 66–68), Arauzo (2011a: 23, 25), Gisotti et al. (2021: 123–125).

##### Description.

***Spores*** (4.0–)4.5–4.8–5.2(–5.4) × (2.9–)3.5–3.7–4.0(–4.2) μm (64/1/1), Q = (1.10–)1.21–1.31–1.40(–1.51), V = (19.7–)29.2–35.2–41.3(–48.0) μm^3^, broadly ellipsoid to ellipsoid, slightly amygdaliform in side view, walls up to 0.8 µm thick and dextrinoid at maturity, smooth, colorless in L4, hilar appendix prominent, 0.8–1 µm long. ***Basidia*** 21–25 × 6.5–8.0 µm, some with up to 1 μm thick wall (crassobasidia), tetrasporic, rarely bisporic to monosporic, clavate, sterigmata up to 4.5 µm long. ***Hymenophoral trama*** regular to subregular, composed of up to 12 µm wide cylindrical to inflated hyphae, colorless in L4. ***Cheilocystidia*** 15–30(–34) × 3–7(–11) µm, rare, scattered, basidiola-like to irregularly cylindrical, lageniform, sometimes lobed to furcate. ***Pleurocystidia*** present, very rare, similar to cheilocystidia but bigger, up to 56 × 12 µm. ***Pileipellis***: suprapellis (at the pileus centre) a transition between trichoderm and epithelium consisting of loosely entangled ascending and erect pluriseptate hyphae, terminal elements broadly cylindrical, clavate to subglobose, up to 18 µm wide; towards the pileus margin it tends to become a cutis with repent hyphae; pigment abundant, brownish grey, bluish, intracellular and encrusting (extracellular), green in KOH; pileitrama consisting of up to 8 µm wide cylindrical hyphae. ***Stipitipellis*** consisting of 2–4 µm wide, multiseptate, parallel, cylindrical hyphae. ***Stipititrama*** similar to the stipitipellis structure but with 3–6 µm wide hyphae. ***Caulocystidia*** 10–70(–80) × 3.0–7 µm, filiform, sinuous, often multiseptate, sometimes irregularly lobed and furcate. ***Clamp-connections*** present everywhere.

##### Specimen examined.

ITALY • Liguria, Pegli (GE), 95 m, in an area of shrub-like Mediterranean vegetation with *Pinuspinaster* Aiton, *Arbutusunedo* L., *Ericaarborea* L., *Cistussalvifolius* L., and *Quercusilex* L., on poor acidic soil with serpentine bedrock, in the needle litter of *P.pinaster*, 6 December 2016, leg. D. Gisotti & F. Boccardo (GDOR:M3986).

##### Notes.

The species was originally described from Spain (Arnolds et al. 2003; Arauzo 2011a) and then recently found in Liguria, Italy (Gisotti et al. 2021). *Pseudobaeosporacyanea* is clearly distinguished from the other species by a unique combination of features such as a bright bluish-purple pileus, pale lamellae and stipe, a trichodermic structure of the pileipellis (with inflated elements), green reaction of the pileipellis to KOH and well-developed cheilocystidia (Arnolds et al. 2003). Our microscopic analysis of the collection studied by Gisotti et al. (2021) (GDOR:M3986) revealed the presence of rare pleurocystidia which were not recorded by previous authors. Pleurocystidia were so far only reported for *P.aciculifera* Voto & Soop (Voto and Soop 2018), and *P.taluna* (Craig et al. 2023), two species of the southern hemisphere. Both Spanish and Italian collections were made in Mediterranean areas in winter, viz. Mediterranean hills with *Pinuspinaster*, *Arbutusunedo* and *Ericaarborea* (Arnolds et al. 2003), and an area of shrub-like Mediterranean vegetation with *P.pinaster*, *A.unedo*, *E.arborea*, *C.salvifolius*, and *Q.ilex* (Gisotti et al. 2021), respectively. The morphology of the Spanish and Italian collections compared was completely consistent.

In the multigene analysis (Fig. 2) the most closely allied species is *P.lilacina* X.D. Yu, Ming Zhang & S.Y. Wu from China (Wu et al. 2017), which is distinguished by a pileus surface pale mauve, colonial buff when old, smaller spores 2.5–3.5(–4.5) × 3–5(–6.5) μm, cheilocystidia absent, and pileipellis hyphae not changing color in 5% KOH.

*Pseudobaeosporapallidifolia* Bas, A. Gennari & Robich from mossy coniferous forest in Tuscany (Italy) so far known only from the type locality, is similar to *P.cyanea* by the dark pileus with paler margin strongly contrasting with whitish to pinkish cream lamellae and the pale stipe, but differs by lack of cheilocystidia, and comparatively large and more elongated ellipsoid spores 4.4–6.4 × 3.3–4.4 μm, Q = 1.30–1.40 (Bas et al. 1997; Bas 2003).

**Figure 13.**
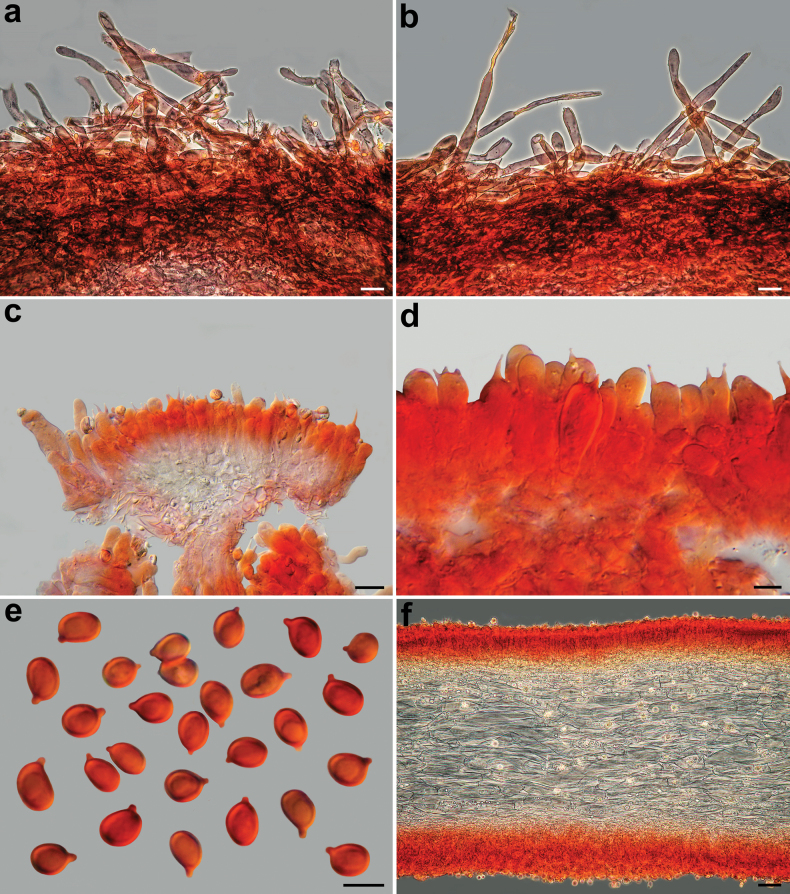
*Pseudobaeosporacyanea*, microscopic features (GDOR M3986). **a, b** Pileipellis; **c** hymenium (basidia and cheilocystidia); **d** basidia; **e** spores; **f** hymenophoral trama; **a–f** in ammoniacal Congo red. Scale bars: 20 µm (**a, b, f**); 10 µm (**c**); 5 µm (**d, e**). Photos by L. Setti.

#### ﻿ Pseudobaeospora calcarea

Taxon classificationFungiAgaricalesTricholomataceae

/P. terrayi complex

6BE76E59-1F17-542B-A956-022AAE7639CA

Figs 4a, e, f

##### Notes.

*Pseudobaeosporacalcarea* was described from Switzerland occurring among and on mosses [mainly *Hypnumcupressiforme* Hedw., *Dicranumscoparium* Hedw., *Hylocomiumsplendens* (Hedw.) W.P. Schimp., and *Pleuroziumschreberi* (Brid.) Mitt.] in coniferous woods (*Pinussylvestris* with *Quercuspubescens*). It was placed into the informal Albidula group as circumscribed by Bas (2003), encompassing species with white to pale buff basidiomes, clamp-connections, absent cheilocystidia and pileipellis without inflated elements. This species differs from all others of this group by a very acrid taste, a negative reaction with KOH (no yellow discoloration) and the absence of clamp-connections in most of the trama and pileipellis hyphae (Clémençon and Ayer 2007). Adamčík and Jančovičová (2011) described *P.terrayi*, a minute, not moss-associated species from Slovakia, which was included in the Albidula group due to the white basidiome colour, despite the presence of cheilocystidia. The species was distinguished from *P.calcarea* by very small basidiomes (pileus 5–8 mm wide versus 7–20 mm), more distant lamellae (L = 11–18 versus 16–28), a mild taste, an occasional presence of an unpleasant fishy smell, a yellowish-greenish discoloration in 5% KOH, presence of cheilocystidia, which are somewhat basidia-like, but often broader and sometimes with distinctly thickened walls and/or incrusted surface, and clamp-connections present in all tissues.

In our ITS analysis (Fig. 3) three collections of *P.calcarea*, including a specimen from Switzerland (LIP PAM06090111), and two collections of *P.terrayi* (holotype included, SAV-F:3317) were found intermixed with each other in a strongly supported clade (PP 1.0, ML BP 92%), suggesting a possible conspecificity of *P.calcarea* and *P.terrayi*. Unfortunately, the holotype and isotype collections of *P.calcarea* (kept at LAU) have not been declared available for molecular analysis (Patrice Descombes, personal comm.). Multigene analysis is essential also to confirm that variability within this clade in the ITS region does not correspond to more than one species as documented in *P.pillodii* and *P.deceptiva* complex.

##### Specimen sequenced.

*P.calcarea*: SUISSE, Grisons, Alvaneu-Bad, ripisylve à *Alnusincana*, 1 September 2006, leg. Pierre-Arthur Moreau. 46.66591, 9.64329, (LIP PAM06090111). *P.terrayi*: SLOVAKIA, Laborecká vrchovina Mts., ca. 1 km NE of Svetlice village, the riverside meadow extensively used as pasture, on ground among ca. 15–30 cm tall herbal vegetation composed of *Achilleamillefolium* L., *Agrimoniaeupatoria* L., *Agrostiscapillaris* L., *Dactylisglomerata* L., *Daucuscarota* L., *Festucapratensis* Huds., *F.rubra* L., *Jaceapratensis* Lam., *Leontodonhispidus* L., *Lotuscorniculatus* L., *Pimpinellasaxifraga* L., *Plantagolanceolata* L., *Poapratensis* L., *Thymuspulegioides* L., *Tithymaluscyparissias* L., *Trifoliumrepens* L., *Veronicachamaedris* L. and *Violahirta* L., 458 m, coord. 49°11'12.9"N, 22°02'55.8"E, 23 Oct 2007, leg. J. Terray (SAV-F:3317 holotype); Kremnické vrchy Mts., Tajov, pasture W of the village centre, 24 October 2020, leg. S. Adamčík (SAV-F:20813).

## ﻿Discussion

### ﻿Phylogenetic position of *Pseudobaeospora*

Taxonomic position of the genus *Pseudobaeospora* has long been debated, swinging from *Agaricaceae* Chevall. tribus *Lepioteae*/or tribus *Cystodermateae* (Singer 1951, 1962, 1963, 1975, 1986; Locquin 1952; Horak 1964, 2005; Aberdeen 1992; Wasser 2002) to *Tricholomataceae* s.l. (Singer 1942; Heinemann 1978; Kühner 1980; Bas 1995, 2003; Bon 1999; Kirk et al. 2008; Vellinga 2009; Læssøe 2008, 2012; Kříž 2018). The dextrinoid nature of the thickened mature spore wall of *Pseudobaeospora* was overemphasized by the first authors as an important argument for placing it close to the lepiotoid fungi.

Morphological arguments stressed for placing *Pseudobaeospora* in the *Tricholomataceae* are (I) the shape of the basidiomes, which is more collybioid than lepiotoid; II) the continuous context at the connection of the stipe and the pileus; (III) the attachment of the lamellae, which is only exceptionally free, but mostly adnate-emarginate, and sometimes even adnate; and (IV) the absence of any sign of a veil (but the ontogeny of the basidiome has not yet been studied). However, *Pseudobaeospora* shows a combination of features such as small-sized mycenoid to collybioid basidiomes, surfaces reacting with KOH, small thick-walled dextrinoid spores which is unique or aberrant in this family.

*Pseudobaeosporapyrifera* was the first species of the genus included in a molecular phylogenetic analysis based on 28S (LSU) rDNA and ITS data, highlighting that it does not belong to the *Agaricaceae* and it showed affinities with *Tricholoma* (*T.focale* (Fr.) Ricken), *Entoloma* (*Entolomataceae* Kotl. & Pouzar), *Thaxterogaster* Singer (*Cortinariaceae* Singer), and *Cystoderma* Fayod (*Squamanitaceae* Jülich) (Vellinga 2003, 2004). Ribosomal DNA sequence analysis by Desjardin et al. (2014), placed *P.pyrifera* and *P.wipapatiae* as sister (but without statistical support) to a clade consisting of *Leucopaxillusalbissimus* (Peck) Singer and some *Tricholoma* species. In both the nrITS and nrLSU separate sequence analyses by Wu et al. (2017), four *Pseudobaeospora* species (*P.lilacina*, *P.pyrifera*, *P.deckeri* and *P.wipapatiae*) were placed sister (PP = 1) to *Callistosporiumgraminicolor* Lennox (*Callistosporiaceae*, *Tricholomatineae*). In multigene phylogenetic analyses (Sánchez-García and Matheny 2017; He et al. 2019), however always based on a small number of *Pseudobaeospora* taxa and not including the type species, *Pseudobaeospora* clustered in *Tricholomataceae* s.s, as delimited by Sánchez-García et al. (2014), in the suborder *Tricholomatineae* (Dentinger et al. 2015). This latter placement was also supported in the analyses by Sánchez-García et al. (2020, 2021) and He and Yang (2022).

As highlighted by the present molecular analyses (Figs 1, 2), all *Pseudobaeospora* species group into a monophyletic clade which is sister to a clade corresponding to *Tricholomataceae* sensu Sánchez-García et al. (2014) and Vizzini et al. (2016). The segregation of *Pseudobaeospora* into its own subfamily leads the core of *Tricholomataceae* (*Tricholomataceae* subfam, *Tricholomatoideae*) becoming more homogeneous.

### ﻿Intrageneric taxa

Only two proposals for the intrageneric classification of *Pseudobaeospora* species were provided in the literature, both based on morphological characters (Bas 2003; Voto 2009, 2015, 2021). Bas (2003) divided the genus into five (morpho)groups (Albidula, Celluloderma, Pyrifera, Frieslandica, and Pillodii) based primarily on basidiome colour and the presence or absence of clamp-connections, presence or absence of cheilocystidia and type of pileipellis (cutis, hymenodermoid, not hymenodermoid). Voto (2009, 2021) recognized in *Pseudobaeospora* two sections, P.sect.Anistoderma (species with hymeniform pileipellis) and P.sect.Pseudobaeospora (species with non-hymeniform pileipellis further divided into the two subsections *Pseudobaeospora* for the species with a trichoderm and *Holocutis* for those with a cutis) only (exclusively) relied on the differences in the structure of the pileipellis. These intrageneric taxa are deemed to be probably artificial by Bas and Voto themselves.

Recent preliminary evidence (see Gisotti et al. 2021; Craig et al. 2023) show that morphology-based sections and subsections as circumscribed by Voto (2009, 2015, 2021) are not consistent with phylogeny, in particular the separation of species with a hymeniform pileipellis in P.sectionAnistoderma from those with a trichoderm or cutis in P.sectionPseudobaeospora. In particular, Craig et al. (2023) recovered, from their phylogeny (but based only on ITS and a poor taxon sampling), two highly supported main clades which they named as the *P.calcarea* clade (= *P.pillodii* clade in the present study), consisting of *P.aphana* Vellinga, *P.calcarea* and *P.celluloderma* (collections from USA), and the *P.pyrifera* clade, consisting of *P.cyanea*, *P.deckeri*, *P.lilacina*, *P.pyrifera*, *P.taluna* and *P.wipapatiae*. Our phylogenetic analysis based on a larger taxon sampling (including also the type species) led to the similar conclusions (Fig. 3). The same two major clades (the core of *Pseudobaeospora*) were recognized which included the same species as in Craig et al. (2023) and, in addition, the *P.pyrifera* clade also contained *P.jamonii*, P.laguncularisvar.denudata and *P.stevensii*; the *P.pillodii* clade also *P.pillodii*, *P.deceptiva*, *P.terrayi*, and *P.celluloderma* (European collections). *Pseudobaeosporabrunnea*, originally described from Spain (Arauzo 2011a, b; Rubio Casa and Palazòn 2019; Bañares Baudet and Moreno 2022) then found also in France (Moyne and Moingeon 2018) but occurring also in Estonia and Georgia based on some environmental sequences (corresponding to UNITE species hypothesis SH1111686) (Fig. 3), is placed outside the core *Pseudobaeospora* species.

Both *P.wipapatiae* (of the *P.pyrifera* clade) and *P.celluloderma* (of the *P.pillodii* clade), which were placed by Voto (2021) in P.sectionAnistoderma, do not form a separate clade but are each intermingled with species of P.sectionPseudobaeospora. The six species of the *P.pillodii* clade all lack cheilocystidia (except *P.terrayi* where they are basidiola-like), whereas among the species of the *P.pyrifera* clade, cheilocystidia are usually present, albeit often not well-differentiated. Secondly, in the *P.pillodii* clade, there is not a distinct reaction to KOH (or at most a pale brownish or greenish reaction in *P.celluloderma* and *P.terrayi*), while in the *P.pyrifera* clade, seven out of the nine species have a strong reaction in KOH, becoming either blue-green, green, or ruby red (*P.wipapatiae*).

A highly supported clade (PP 1.0, ML BP 92%) within the *P.pillodii* clade, consisted of *P.calcarea*/*P.terrayi*, *P.aphana* and *Pseudobaeospora* sp. (TENN 070699/CCB143666, collected by C.C. Braaten) which are all taxa characterized by whitish basidiomes (Clémençon and Ayer 2007; Vellinga 2009; Adamčík and Jančovičová 2011; P.B. Matheny, pers. comm.).

In the highly supported clade (PP 1.0, ML BP 100%) within the *P.pyrifera* clade, formed by *P.pyrifera* (including *P.mutabilis*), *P.jamonii*, *P.wipapatiae* and *P.deckeri*, all the species share violet tinges on pileus, a strong reaction in KOH and presence of clamp-connections, presence of cheilocystidia (except for *P.deckeri*); but the pileipellis structure is very different from simply hymenodermic (*P.pyrifera*/*P.mutabilis*), hymenodermic with pileocystidia (*P.wipapatiae*) or trichodermic (*P.deckeri*), to a cutis with a few ascending hyphae (*P.jamonii*).

Two major sister (PP 1.0, ML BP 66%) clades corresponding to the *P.pillodii* clade and the *P.pyrifera* clade were also obtained in the multigene tree (Fig. 2): the first (PP 1.0, ML BP 59%) formed by *P.pillodii*, *P.celluloderma* and P.aff.celluloderma; the second (PP 1.0, ML BP 100%) by *P.lilacina*, *P.cyanea*, *P.jamonii*, *P.wipapatiae* and *P.pyrifera*.

Whereas the KOH reactions, the presence of cheilocystidia (when well-developed) can be useful systematic markers at an interspecific and/or supraspecific level, and the pileipellis structure only at interspecific level, the presence of mono-bisporic versus tetrasporic basidia is not a species discriminating character. For example, as already pointed out by Craig et al. (2023), an entirely bisporic collection of *P.taluna* from continental Australia (Victoria, MEL 2363200 OQ457539) is conspecific with the tetrasporic collections from Tasmania (Fig. 3), and bisporic/tetrasporic collections of *P.pillodii* are conspecific with the entirely tetrasporic ones. Some species are known to possess bisporic and tetrasporic basidia even either on the same lamella or in separate collections (e.g., *P.pillodii*, *P.taluna*…). Contrary to that, the presence of clamp-connections was consistent within collections of each species and only *P.pillodii* and *P.deceptiva* lack clamp-connections.

## ﻿Conclusions

This study is the first effort to link morphology-based classification of *Pseudobaeospora* with phylogenetic data. Twenty-six *Pseudobaeospora* collections corresponding to eleven species (five types) were newly sequenced. *Pseudobaeospora* occupied a unique position within *Tricholomataceae* and deserved to be placed in a subfamily of its own. Multigenic analyses conducted on a larger number of species will be needed for better understanding of phylogenetic relationships within the genus and for testing the support for clades established so far mainly based on ITS sequences. Multiloci data and larger taxon sampling are also essential to understand phylogenetic history and origin of the genus *Pseudobaeospora*. Our ITS analysis demonstrated that the genus is almost globally distributed (also an African collection representative ITS sequence is present) and with two Australian clades mixed with other members of *P.pyrifera* clade from Northern Hemisphere. It suggests a Pangean origin with multiple migration events.

In general, a lot of synonymy and disagreement in recognition of *Pseudobaeospora* species is due to overemphasizing spore dimensions that should always be interpreted together with the number of sterigmata on basidia. Furthermore, pileipellis structure that is influenced by the basidiome development and/or placement on the pileus, has been interpreted in different ways by various authors and the terminology used to describe it is often not accurate.

## Supplementary Material

XML Treatment for Tricholomataceae section Pseudobaeosporoideae

XML Treatment for Pseudobaeospora pillodii

XML Treatment for Pseudobaeospora deceptiva

XML Treatment for Pseudobaeospora pyrifera

XML Treatment for Pseudobaeospora jamonii

XML Treatment for Pseudobaeospora celluloderma

XML Treatment for Pseudobaeospora cyanea

XML Treatment for Pseudobaeospora calcarea

## ﻿Additional information

### Author ORCIDs

Alfredo Vizzini https://orcid.org/0000-0001-8390-6446

Giovanni Consiglio https://orcid.org/0000-0001-8788-5195

Katarína Adamčíková https://orcid.org/0000-0001-5407-5233

Slavomír Adamčík https://orcid.org/0000-0003-2156-5767

### Data availability

The ITS and combined alignments used for the phylogenetic analyses are deposited in Figshare (https://doi.org/10.6084/m9.figshare.28351739). The newly generated sequences are deposited in GenBank as reported in the main text.

## Supplementary materials

Supplementary material 1 List of specimens Data type docx Explanation note List of specimens used in the molecular analyses. Newly generated sequences are in bold. https://binary.pensoft.net/file/1279441This dataset is made available under the Open Database License (http://opendatacommons.org/licenses/odbl/1.0/). The Open Database License (ODbL) is a license agreement intended to allow users to freely share, modify, and use this Dataset while maintaining this same freedom for others, provided that the original source and author(s) are credited.Alfredo Vizzini, Giovanni Consiglio, Katarína Adamčíková, Ledo Setti, Slavomír Adamčík

Supplementary material 2 Bayesian inference phylogram Data type pdf Explanation note Bayesian inference phylogram built with nucleotide sequence data of four loci (nrLSU, nrSSU, *RPB2*-exons and *TEF1*-exons) of the main lineages inside the suborder *Tricholomatineae* of the order *Agaricales*, rooted with *Suilluspictus* (*Boletales*), *Ampulloclitocybeclavipes* and *Pseudoarmillariellaectypoides* (*Hygrophorineae*, *Agaricales*) as outgroups. All family-level clades were not collapsed. Nodes were annotated with Bayesian PP (left) and ML BP (right) values, with the significance threshold considered as Bayesian PP ≥ 0.95 and/or ML BP ≥ 70%. Subsignificant support values were annotated in parentheses. Boldface names represent samples sequenced for this study. https://binary.pensoft.net/file/1279438This dataset is made available under the Open Database License (http://opendatacommons.org/licenses/odbl/1.0/). The Open Database License (ODbL) is a license agreement intended to allow users to freely share, modify, and use this Dataset while maintaining this same freedom for others, provided that the original source and author(s) are credited.Alfredo Vizzini, Giovanni Consiglio, Katarína Adamčíková, Ledo Setti, Slavomír Adamčík
