# Supplementary material for: ﻿Pseudobaeosporoideae, a new subfamily within the Tricholomataceae for the genus Pseudobaeospora (Agaricales, Tricholomatineae) based on morphological and molecular inference
Source: IMA Fungus. 2025 Mar 13;16:e144994. doi: 10.3897/imafungus.16.144994 (PMC11926610; doi:10.3897/imafungus.16.144994)
Supplement: Supplementary material 1 — List of specimens [file imafungus-16-e144994-s001.docx]

Supplementary Table 1. List of specimens used in the molecular analyses. Newly generated sequences are in bold.

| Taxa | Voucher/strain | INSDC (GenBank)/UNITE/BOLD accession number | | | | | Locality | Reference (see the main text) |
| --- | --- | --- | --- | --- | --- | --- | --- | --- |
|  |  | nrITS | nrLSU | nrSSU | *RPB2* | *TEF1* |  |  |
| *Alboleptonia stylophora* | AST84 TB8475 | – | GU384610 | – | GU384633 | – | not indicated | Baroni et al. (2011) |
| *Albomagister* sp. 2 | TENN MSG136 | KJ417248 | – | – | KJ424364 | – | USA | Sánchez-García et al. (2014) |
| *Albomagister* sp. 2 | TENN MSG137 | KJ417247 | KJ417178 | – | KJ424363 | – | USA | Sánchez-García et al. (2014) |
| *Albomagister* sp. 2 | MICH:00058090/MSG-2014/AHS14872 | KJ417249 | – | – | – | – | USA | Sánchez-García et al. (2014) |
| *Albomagister* sp. 3 | TENN 065323 | KJ417250 | KJ417179 | – | KJ424365 | – | USA | Sánchez-García et al. (2014) |
| *Albomagister subaustralis* | MICH 010951 | KJ417262 | – | – | – | – | USA | Sánchez-García et al. (2014) |
| *Albomagister subaustralis* | TENN 014423 | KJ417254 | – | – | – | – | USA | Sánchez-García et al. (2014) |
| *Albomagister subaustralis* | TENN 017951 | KJ417261 | – | – | – | – | USA | Sánchez-García et al. (2014) |
| *Albomagister subaustralis* | TENN 020463 | KJ417257 | – | – | – | – | USA | Sánchez-García et al. (2014) |
| *Albomagister subaustralis* | TENN 022663 | KJ417259 | – | – | – | – | USA | Sánchez-García et al. (2014) |
| *Albomagister subaustralis* | TENN 023195 | KJ417260 | – | – | – | – | USA | Sánchez-García et al. (2014) |
| *Albomagister subaustralis* | TENN 023221 | KJ417255 | – | – | – | – | USA | Sánchez-García et al. (2014) |
| *Albomagister subaustralis* | TENN 064620 | KJ417252 | KJ417181 | KJ417155 | KJ424367 | – | USA | Sánchez-García et al. (2014) |
| *Albomagister subaustralis* | TENN 064621 | KJ417251 | KJ417180 | – | KJ424366 | – | USA | Sánchez-García et al. (2014) |
| *Albomagister subaustralis* | TENN 066902 | KJ417253 | – | – | – | – | USA | Sánchez-García et al. (2014) |
| *Albomagister subaustralis* | TENN 067343 | KJ417263 | – | – | – | – | USA | Sánchez-García et al. (2014) |
| *Albomagister subaustralis* | TENN 8018628 | KJ417258 | – | – | – | – | USA | Sánchez-García et al. (2014) |
| *Ampulloclitocybe clavipes* | AFTOL-ID 542/PBM2474 |  | AY639881 | AY771612 | AY780937 | AY881022 | USA | Matheny PB, Hughes KW, Petersen RH, Hibbett DS, AFTOL, direct submission |
| *Anupama indica* | AMH 10031 | MH989588 | MH989584 | – | MH992116 | – | India | Raj et al. (2019) |
| *Anupama indica* | AMH 10032 | MH989589 | MH989585 | – |  | – | India | Raj et al. (2019) |
| *Anupama indica* | AMH 10033 | MH989590 | MH989586 | – | MH992117 | – | India | Raj et al. (2019) |
| *Anupama indica* | CAL 1725 | MH989587 | MH989583 | – | – | – | India | Raj et al. (2019) |
| *Asterophora parasitica* | CBS 683.82 | AF357038 | AF223191 | – | EF420988 | EF421054 | not indicated | Hofstetter et al. (2002); Moncalvo et al. (2002) |
| *Atractosporocybe inornata* | TO AV261012d | KJ680993 | KJ681046 | KJ681075 | KJ681067 | KJ681090 | Italy | Alvarado et al. (2015) |
| *Atractosporocybe inornata* | TO AV261012h | KJ680994 | KJ681045 | – | KJ681066 | KJ681089 | Italy | Alvarado et al. (2015) |
| *Bonomyces afrosinopicus* | LIP LYK13040015 | MG696613 | MG696624 | MG696621 | MG702593 | MG702590 | Algeria | Alvarado et al. (2018b) |
| *Bonomyces arnoldii* | TO AV051217 | MG696616 | MG696625 | MG696622 | MG702594 | MG702591 | Italy | Alvarado et al. (2018b) |
| *Bonomyces arnoldii* | TO AV190511 | MG696618 | MG696626 | – | – | – | Italy | Alvarado et al. (2018b) |
| *Bonomyces sinopicus* | KATO-Fungi 3689 | MG696619 | MG696627 | MG696623 | MG702595 | MG702592 | Turkey | Alvarado et al. (2018b) |
| *Callistosporium brunnescens* | DAOM 34832 | – | AF261407 | – | – | – | not indicated | Moncalvo et al. (2002) |
| *Callistosporium elaeodes* | ZT Myc 2322 | MN017504 | MN017445 | MN017561 | – | – | France | Vizzini et al. (2020a) |
| *Callistosporium elaeodes* | ARAN Fungi 3008411 | MN017506 | MN017447 | MN017563 | – | – | Spain | Vizzini et al. (2020a) |
| *Callistosporium elaeodes* | ZT Myc 58268 | MN017505 | MN017446 | MN017562 | MN018838 | MN026900 | France | Vizzini et al. (2020a) |
| *Callistosporium elegans* | CORT 013277 | MN017507 | MN017448 | MN017564 | – | – | Puerto Rico | Vizzini et al. (2020a) |
| *Callistosporium elegans* | CORT 013382 | MN017508 | MN017449 | MN017565 | MN018839 | MN026901 | Puerto Rico | Vizzini et al. (2020a) |
| *Callistosporium elegans* | CORT 013860 | MN017509 | MN017450 | MN017566 | – | – | Dominican Republic | Vizzini et al. (2020a) |
| *Callistosporium elegans* | CFMR BZ-1772 | MN017512 | MN017453 | MN017568 | – | – | Belize | Vizzini et al. (2020a) |
| *Callistosporium graminicolor* | AFTOL-ID 978/WTU PBM2341 | DQ484065 | AY745702 | AY752974 | – | GU187761 | USA | Matheny et al. (2006); Binder et al. (2010);  Matheny PB and Hibbett DS, direct submission |
| *Callistosporium hesleri* | TENN 008084 | HQ179664 | HQ179664 | – | – | – | USA | Matheny PB and Wolfenbarger AD, unpublished |
| *Callistosporium imbricatum  "Pleurocollybia imbricata"* | SFSU DED 8232 | MN017515 | MN017456 | MN017570 | – | – | Republic of São Tomé and Príncipe | Vizzini et al. (2020a) |
| *Callistosporium imbricatum  "Pleurocollybia imbricata"* | BRH TJB9847 | HM105568 | HM105568 | HM105568 | HM105567 | – | Republic of São Tomé and Príncipe | Baroni et al. (2008) |
| *Callistosporium luteo-olivaceum* | AMB 18228 | MN017516 | MN017457 | MN017571 | MN018841 | – | Italy | Vizzini et al. (2020a) |
| *Callistosporium luteo-olivaceum* | IB 19770276 | AF325667 | AF261406 | – | – | – | Austria | Peintner et al. (2001); Moncalvo et al. (2002) |
| *Callistosporium luteo-olivaceum* | JM 99/124 and VHAs09/02 | AF325666 | AF261405 | – | DQ825406 | KP255477 | not indicated | Peintner et al. (2001); Moncalvo et al. (2002) |
| *Callistosporium pinicola* | WRSL 0512 | MN017520 | MN017461 | MN017573 | – | – | Poland | Vizzini et al. (2020a) |
| *Callistosporium pinicola* | BRNM 734327 | MN017521 | MN017462 | – | – | – | Czech Republic | Vizzini et al. (2020a) |
| *Callistosporium pinicola* | BRNM 793115 | MN017522 | MN017463 | MN017574 | MN018843 | – | Czech Republic | Vizzini et al. (2020a) |
| *Callistosporium praemultifolium "Pleurocollybia praemultifolia"* | SFSU-F DED 8238 | MN017524 | MN017464 | MN017575 | MN018844 | – | Republic of São Tomé e Príncipe | Vizzini et al. (2020a) |
| *Callistosporium pseudofelleum* (*"Clitocybe* aff. *fellea"*) | CUW PBM2825 | EF416919 | EF416918 | – | – | – | USA | Ammirati et al. (2007) |
| *Callistosporium pseudofelleum* (*"Clitocybe* aff. *fellea"*) | TENN 062782, PBM3028 | HQ728533 | HQ728534 | HQ728535 | HQ728536 | – | USA | Matheny et al. (2014) |
| *Calocybe ionides* | HC 77.133 | – | AF223179 | – | EF420991 | EF421057 | not indicated | Moncalvo et al. (2002) |
| *Catathelasma imperiale* | TUR 062105 | MN017529 | MN017469 | MN017577 |  |  | Finland | Vizzini et al. (2020a) |
| *Catathelasma imperiale* | TUR 111275 | MN017530 | MN017470 | MN017578 | MN018847 | MN026902 | Finland | Vizzini et al. (2020a) |
| *Catathelasma imperiale* | TUR 140116 | MN017531 | MN017471 | MN017579 | MN018848 | MN026903 | Finland | Vizzini et al. (2020a) |
| *Catathelasma imperiale* | TUR 186494 | MN017532 | MN017472 | MN017580 | – | – | Finland | Vizzini et al. (2020a) |
| *Catathelasma ventricosum* | TUR 10472 | MN017538 | MN017478 | – | – | – | Canada | Vizzini et al. (2020a) |
| *Catathelasma ventricosum* | DAOM 221514 | MN017536 | MN017476 | MN017584 | MN018850 | – | USA | Vizzini et al. (2020a) |
| *Catathelasma ventricosum* | DAOM 225247 | MN017537 | MN017477 | MN017585 | MN018851 | MN026906 | USA | Vizzini et al. (2020a) |
| *Cleistocybe carneogrisea* | TENN 063842 | HQ728526 | HQ728527 | HQ728528 | – | – | France | Matheny et al. (2014) |
| *Cleistocybe vernalis* | WTU ADP050506 | EF416917 | EF416916 | – | – | – | USA | Ammirati et al. (2007) |
| *Cleistocybe vernalis* | AFTOL-ID 721/WTU PBM1856 | DQ486692 | AY647208 | DQ092913 | – | – | USA | Matheny et al. (2006); Ammirati et al. (2007) |
| *Clitocella fallax "Rhodocybe fallax"* | CBS 129.63 | – | AF223166 | – | EF421018 | EF421089 | Switzerland | Moncalvo et al. (2002); Hofstetter et al. (2002) |
| *Clitocella mundula "Rhodocybe mundula"* | AFTOL-ID 521/CORT TJB7599 | DQ494694 | AY700182 | DQ089017 | DQ474128 | KC816863 | not indicated | Matheny et al. (2006); Matheny PB, Baroni TJ and Hibbett DS; Matheny PB, Curtis JM and Hibbett DS, AFTOL, direct submission |
| *Clitocella popinalis "Clitopilus popinalis"* | ME Noordeloos 9867 | – | GQ289213 | – | GQ289280 | – | not indicated | Co-David et al. (2009) |
| *Clitocybe dealbata* | IE-BSG HC 95.cp3 | – | AF223175 | DQ825431 | DQ825407 | EF421080 | not indicated | Moncalvo et al. (2002); Hofstetter et al. (2002) |
| *Clitocybe ditopa* | AMB 19311 | OR863426 | OR863496 | OR863563 | OR828253 | OR828314 | Italy | Vizzini et al. (2024) |
| *Clitocybe nebularis* | CBS 362.65 | – | AF223217 | – | EF421011 | EF421081 | Switzerland | Hofstetter et al. (2002) |
| *Clitocybe nebularis "Lepista nebularis"* | AFTOL ID 1495/WTU PBM2259 | – | DQ457658 | DQ437681 | DQ470833 | – | USA | Hofstetter et al. (2002); Matheny et al. (2006); Matheny PB, Curtis JM and Hibbett DS, AFTOL, direct submission |
| *Clitocybe odora* | ZRL 20152003 | – | KY418888 | KY418953 | KY419033 | KY419081 | China | Zhao et al. (2017) |
| *Clitocybe subditopoda* | AFTOL-ID 533/PBM 2489 | – | AY691889 | AY771608 | AY780942 | DQ408150 | not indicated | Hofstetter et al. (2002); Matheny et al. (2007); Matheny PB and Hibbett DS, AFTOL, direct submission |
| *Clitolyophyllum akcaabatense* | KATO Fungi 3184 | – | KT934394 | – | KT934395 | – | Turkey | Sesli et al. (2016) |
| *Clitopaxillus alexandri* | TO AV45634 | – | MG321393 | MG321329 | MG334546 | MG334537 | Italy | Alvarado et al. (2018a) |
| *Clitopaxillus fibulatus* | TO AV190916 | – | MG321394 | MG321330 | MG334547 | – | Italy | Alvarado et al. (2018a) |
| *Clitopilopsis hirneola* | CBS 577.87 | – | AF223163 | – | GU384645 | – | Switzerland | Moncalvo et al. (2002); Baroni et al. (2011) |
| *Clitopilus apalus* | M536 | – | AF261287 | – | KC816906 | KC816822 | not indicated | Moncalvo et al. (2002); Kluting et al. (2014) |
| *Clitopilus cystidiatus* | ME Noordeloos 200350 | – | GQ289147 | – | GQ289220 | – | not indicated | Co-David et al. (2009) |
| *Clitopilus orientalis* | CAL 1613 | – | MG321558 | – | MG321559 | – | India | Anil Raj and Manimohan (2018) |
| *Clitopilus prunulus* | TB8229 | – | GU384615 | – | GU384650 | – | not indicated | Baroni et al. (2011) |
| *Clitopilus prunulus* | VHAs07/02 | – | EF421092 | – | DQ825408 | EF421086 | not indicated | Hofstetter et al. (2002) |
| *Collybia tuberosa* | AFTOL-ID 557/TENN 53540 | AY854072 | AY639884 | AY771606 | AY787219 | AY881025 | not indicated | Matheny PB, Hughes KW, Petersen RH and Hibbett DS; Matheny PB, Hughes KW, Petersen RH, AFTOL, direct submission |
| *Corneriella bambusarum* | NYBG:DED5462 | KJ417264 | KJ417185 | – | KJ424370 | – | USA | Sánchez-García et al. (2014) |
| *Corneriella bambusarum* | NYBG:00775752/DED 6572 | KJ417265 | – | – | – | – | USA | Sánchez-García et al. (2014) |
| *Corneriella humicola* | NYBG:DED7871 | KF291051 | KF291052 | KF291053 | – | – | Thailand | Lodge et al. (2013) |
| *Corneriella* sp. | K(M) 188911 strain JPF943A | KJ417324 | KJ417234 | – | – | – | Martinique | Sánchez-García et al. (2014) |
| *Dennisiomyces glabrescentipes* | K(M) 188910 | KJ417326 | KJ417235 | – | – | – | Martinique | Sánchez-García et al. (2014) |
| *Dennisiomyces* *griseus* | K(M)160882 | KJ417325 | – | – | – | – | Trinidad and Tobago | Sánchez-García et al. (2014) |
| *Dennisiomyces* sp. 1 | CFMR BZ-4245 | KF291063 | KF291064 | KF291065 | KF291066 | – | Belize | Lodge et al. (2013) |
| *Dennisiomyces* sp. 2 | CFMR PR-4733 | KF306336 | KF306337 | – | KF306338 | – | Puerto Rico | Lodge et al. (2013) |
| *Dennisiomyces* sp. 2 | CFMR PR-4763 | KJ417269 | KJ417191 | – | – | – | Puerto Rico | Sánchez-García et al. (2014) |
| *Dennisiomyces* sp. 3 | CFMR PR-4764 | KJ417270 | – | – | – | – | Puerto Rico | Sánchez-García et al. (2014) |
| *Dennisiomyces* sp. 4 | CFMR PR-6334 | KJ417271 | – | – | – | – | Puerto Rico | Sánchez-García et al. (2014) |
| *Dennisiomyces* sp. 4 | CFMR PR-6613 | KJ417268 | KJ417190 | KJ417157 | – | – | Puerto Rico | Sánchez-García et al. (2014) |
| *Dennisiomyces* sp. 5 | TENN 061833 | KJ417327 | KJ417239 | – | – | – | USA | Sánchez-García et al. (2014) |
| *Dennisiomyces* sp. 5 | TENN 061851 | KJ417328 | – | – | – | – | USA | Sánchez-García et al. (2014) |
| *Dennisiomyces* sp. 5 | TENN 067232 | – | KJ417186 | – | KJ424371 | – | USA | Sánchez-García et al. (2014) |
| *Dennisiomyces* sp. 5 | TENN 067388/MSG-2014 strain BPL254 | KJ417266 | KJ417188 | – | – | – | USA | Sánchez-García et al. (2014) |
| *Dennisiomyces* sp. 5 | TENN 067462/MSG-2014 strain PBM3861 | KJ417267 | KJ417189 | KU058567 | KJ424374 | – | USA | Sánchez-García et al. (2014) |
| *Dennisiomyces* sp. 5 | TENN 067469 | – | KJ417187 | – | KJ424372 | – | USA | Sánchez-García et al. (2014) |
| *Dermoloma alexandri* | SAV F-4147 | MW193771 | MW208870 | – | MW080854 | – | Estonia | Sánchez-García et al. (2021) |
| *Dermoloma atrocinereum* | SAV F-4138 | MW193797 | MW208872 | – | MW080857 | – | Slovakia | Sánchez-García et al. (2021) |
| *Dermoloma bellerianum* | SAV F-20027 | MW193804 | MW208906 | – | MW080888 | – | Slovakia | Sánchez-García et al. (2021) |
| *Dermoloma cuneifolium* | SAV F-4265 | MW193838 | MW208887 | – | MW080865 | – | Romania | Sánchez-García et al. (2021) |
| *Dermoloma pseudocuneifolium* | SAV F-4422 | MW193855 | MW208901 | – | MW080894 | – | United Kingdom | Sánchez-García et al. (2021) |
| *Entocybe nitida* | TB7526 | – | GU384626 | – | GU384655 | – | USA | Baroni et al. (2011) |
| *Entocybe turbida* | TB6949 | – | GU384630 | – | GU384656 | – | USA | Baroni et al. (2011) |
| *Entoloma abortivum* | GDGM 27313/ H. den Bakker 92 | – | JQ320117 | – | GQ289222 | – | China/The Netherlands | He et al. (2013), Co-David et al. (2009) |
| *Entoloma cephalotrichum* | C. Ulje 1997-08-01 | – | GQ289157 | – | GQ289229 | – | The Netherlands | Co-David et al. (2009) |
| *Entoloma conferendum* | ME Noordeloos 200313 | – | GQ289160 | – | GQ289231 | – | Belgium | Co-David et al. (2009) |
| *Entoloma gelatinosum* | G. Gates E792 | – | GQ289165 | – | GQ289236 | – | Australia | Co-David et al. (2009) |
| *Entoloma griseolazulinum* | P. Manimohan 738 | – | GQ289166 | – | GQ289237 | – | India | Co-David et al. (2009) |
| *Entoloma prunuloides* | AFTOL-ID 523/TJB4765 | – | AY700180 | AY665784 | DQ385883 | DQ457633 | not indicated | Matheny et al. (2007); Matheny et al. (2006); Matheny PB, Baroni TJ and Hibbett DS, AFTOL, direct submission |
| *Entoloma sinuatum* | AFTOL-ID 524/TJB5349 | – | AY691891 | AY657007 | – | – | not indicated | Matheny PB, Baroni TJ and Hibbett DS, AFTOL, direct submission |
| *Entoloma tjallingiorum* | J. Vauras 14318F | – | GQ289197 | – | GQ289267 | – | Finland | Co-David et al. (2009) |
| *Entoloma undatum* | ME Noordeloos 200327 | – | GQ289202 | – | GQ289270 | – | Belgium | Co-David et al. (2009) |
| *Entoloma vinaceum* | TB8870 | – | GU384631 | – | GU384651 | – | USA | Baroni et al. (2011) |
| *Entoloma violaceovillosum* | P. Manimohan 645 | – | GQ289205 | – | GQ289273 | – | India | Co-David et al. (2009) |
| *Guyanagarika anomala* | MCA1519/TENN 070919 | KX092095 | KX092109 | KX092115 | KX092146 | – | Guyana | Sanchez-García et al. (2016) |
| *Guyanagarika anomala* | TH7419/TENN 070920 | KX092096 | KX092110 | – | KX092147 | – | Guyana | Sanchez-García et al. (2016) |
| *Guyanagarika aurantia* | MCA1741/TENN 070895 | KX092073 | KX092097 | – | KX092129 | – | Guyana | Sanchez-García et al. (2016) |
| *Guyanagarika aurantia* | TH10068/TENN 070896 | KX092081 | KX092101 | – | KX092135 | – | Guyana | Sanchez-García et al. (2016) |
| *Guyanagarika aurantia* | TH9693/TENN 070902 | KX092078 | KX092098 | KX092111 | KX092132 | – | Guyana | Sanchez-García et al. (2016) |
| *Guyanagarika aurantia* | TH9835/TENN 070903 | KX092079 | KX092099 | KX092112 | KX092133 | – | Guyana | Sanchez-García et al. (2016) |
| *Guyanagarika aurantia* | TH9836/TENN 070904 | KX092080 | KX092100 | KX092113 | KX092134 | – | Guyana | Sanchez-García et al. (2016) |
| *Guyanagarika pakaraimensis* | MCA4749/TENN 070906 | KX092083 | KX092102 | – | KX092137 | – | Guyana | Sanchez-García et al. (2016) |
| *Guyanagarika pakaraimensis* | MCA4775/TENN 070907 | KX092084 | KX092103 | – | KX092138 | – | Guyana | Sanchez-García et al. (2016) |
| *Guyanagarika pakaraimensis* | MCA4776/TENN 070908 | KX092085 | KX092104 | – | KX092139 | – | Guyana | Sanchez-García et al. (2016) |
| *Harmajaea guldeniae* | O-F:64624 | – | MG321397 | MG321332 | – | MG334539 | Norway | Alvarado et al. (2018a) |
| *Harmajaea harperi* | LIP 0401361 | – | MG321399 | MG321333 | MG334549 | MG334541 | USA | Alvarado et al. (2018a) |
| *Harmajaea wellsiae* | TUR 205933 | – | MG321401 | – | MG334550 | MG334542 | Finland | Alvarado et al. (2018a) |
| *Hypsizygus ulmarius* | DUKE JM/HW | – | AF042584 | – | EF420996 | EF421062 | USA | Moncalvo et al. (2000); Hofstetter et al. (2002) |
| *Infundibulicybe geotropa* | ALV4344 | – | KT122793 | – | – | – | Spain | Li et al. (2016) |
| *Infundibulicybe gibba* | AFTOL-ID 1508/CUW JCS0704B | DQ490635 | DQ457682 | DQ115780 | DQ472727 | GU187759 | USA | Matheny et al. (2006); Binder et al. (2010);  Curtis JM, Matheny PB and Hibbett DS, AFTOL, direct submission |
| ***Lepista caespitosa*** | **AMB 19616** | **OR903204** | **OR903218** | **OR889664** | **OR901917** | – | **Italy** | **This study** |
| ***Lepista densifolia*** | **AMB 19615** | **OR903205** | **OR903219** | **OR889665** | **–** | – | **Italy** | **This study** |
| ***Lepista glaucocana*** | **AMB 18862/GC12141** | **OR903206** | **OR903220** | **OR889666** | **–** | – | **Italy** | **This study** |
| *Lepista irina* | AFTOL-ID 815/WTU PBM2291 | – | DQ234538 | AY705948 | DQ385885 | DQ028591 | USA | Matheny et al. (2007) |
| *Lepista ricekii* | AMB 18864 | OR863453 | OR863521 | OR863580 | OR828270 | – | Italy | Vizzini et al. (2024) |
| *Lepista saeva* | TENN 066100 | – | KJ417193 | KJ417159 | KJ424376 | – | USA | Sánchez-García et al. (2014) |
| *Leptonia serrulata* | VHAs0102 | – | GU384624 | – | GU384634 | – | not indicated | Baroni et al. (2011) |
| *Leucocybe candicans "Clitocybe candicans"* | AFTOL-ID 541/PBM 2476 | – | AY645055 | AY771609 | DQ385881 | DQ408149 | USA | Matheny et al. (2007); Matheny PB and Hibbett DS, AFTOL, direct submission |
| *Leucocybe connata "Clitocybe connata" "Lyophyllum connatum"* | DUKE JM90c | – | AF042590 | – | EF420995 | EF421061 | USA | Moncalvo et al. (2000); Hofstetter et al. (2002) |
| *Leucopaxillus albissimus* | FH 00301900 | KJ417274 | – | – | – | – | USA | Sánchez-García et al. (2014) |
| *Leucopaxillus albissimus* | DAOM 182713 | – | AF261393 | – | – | – | not indicated | Moncalvo et al. (2002) |
| *Leucopaxillus albissimus* | FH 00301850 strain F9693 | KJ417273 | – | – | – | – | Canada | Sánchez-García et al. (2014) |
| *Leucopaxillus albissimus* | XAL Landeros 7/8-A | KJ417272 | KJ417194 | – | – | – | Mexico | Sánchez-García et al. (2014) |
| *Leucopaxillus alboalutaceus* | GB 0065210 strain LAS00/082 | KJ417275 | KJ417195 | KJ417161 | KJ424377 | – | Sweden | Sánchez-García et al. (2014) |
| *Leucopaxillus alboalutaceus* | GB 0065215 strain LAS88/79 | KJ417245 | – | – | – | – | Sweden | Sánchez-García et al. (2014) |
| *Leucopaxillus alboalutaceus* | AMB 20102/GC97076 | JQ639147 | – | – | – | – | Italy | Vizzini et al. (2012) |
| *Leucopaxillus amarus* | FH 00301878 | KJ417280 | – | – | – | – | USA | Sánchez-García et al. (2014) |
| *Leucopaxillus amarus* | MICH 0073741 | KJ417279 | – | – | – | – | USA | Sánchez-García et al. (2014) |
| *Leucopaxillus amarus* | WTU 8827 | KJ417278 | KJ417197 | – | – | – | USA | Sánchez-García et al. (2014) |
| *Leucopaxillus amarus* | TENN 064359/IBUG:LGD5690 | KJ417277 | – | – | – | – | Mexico | Sánchez-García et al. (2014) |
| *Leucopaxillus cerealis* | GB 0068845 | KJ417282 | KJ417198 | KJ417162 | KJ424379 |  | Sweden | Sánchez-García et al. (2014) |
| *Leucopaxillus cerealis* | GB 0110964 | KJ417281 | – | – | KJ424378 | – | Sweden | Sánchez-García et al. (2014) |
| *Leucopaxillus cerealis* | GB 065211 | KJ417283 | – | – | KJ424380 | – | Sweden | Sánchez-García et al. (2014) |
| *Leucopaxillus cerealis* | CORT TJB8321 | KJ417284 | – | – | – | – | USA | Sánchez-García et al. (2014) |
| *Leucopaxillus eucalyptorum* | NYBG 01115433 | KJ417285 | KJ417199 | – | – | – | Australia | Sánchez-García et al. (2014) |
| *Leucopaxillus eucalyptorum* | TENN 055040 | KJ417286 | KJ417200 | – | – | – | Argentina | Sánchez-García et al. (2014) |
| *Leucopaxillus gentianeus* | TENN 05616 | – | AF261394 | – | – | – | not indicated | Moncalvo et al. (2002) |
| *Leucopaxillus gentianeus* | GB EL291-11 | KJ417287 | KJ417201 | – | KJ424381 | – | Sweden | Sánchez-García et al. (2014) |
| *Leucopaxillus gracillimus* | XAL:Guzman34476 | KJ417288 | – | – | – | – | Mexico | Sánchez-García et al. (2014) |
| *Leucopaxillus gracillimus* | CORT TJB8315 | KJ417289 | – | – | – | – | USA | Sánchez-García et al. (2014) |
| *Leucopaxillus laterarius* | TENN 029877 | KJ417292 | KJ417204 | – | – | – | USA | Sánchez-García et al. (2014) |
| *Leucopaxillus laterarius* | TENN 044433 | KJ417291 | KJ417203 | – | – | – | USA | Sánchez-García et al. (2014) |
| *Leucopaxillus laterarius* | TENN 063507 | KJ417290 | KJ417202 | KJ417163 | – | – | USA | Sánchez-García et al. (2014) |
| *Leucopaxillus laterarius* | IBUG:ALC178 | KJ417293 | – | – | – | – | Mexico | Sánchez-García et al. (2014) |
| *Leucopaxillus laterarius* | IBUG:VRC1717 | KJ417294 | – | – | – | – | Mexico | Sánchez-García et al. (2014) |
| *Leucopaxillus lilacinus* | TENN 066653 | KJ417295 | KJ417205 | KJ417164 | KJ424382 | – | Australia | Sánchez-García et al. (2014) |
| *Leucopaxillus monticola* | TO AVL20111 | JQ639156 | – | – | – | – | France | Vizzini et al. (2012) |
| *Leucopaxillus paradoxus* | GB 0110968 | KJ417296 | KJ417206 | KJ417165 | KJ424383 | – | Hungary | Sánchez-García et al. (2014) |
| *Leucopaxillus tricolor* | TENN 061725 | KJ417323 | KJ417207 | KJ417166 | KJ424384 | – | USA | Sánchez-García et al. (2014) |
| *Lyophyllum leucophaeatum* | HAe 251.97 | – | AF223202 | DQ367420 | DQ367434 | DQ367427 | not indicated | Moncalvo et al. (2002); Hofstetter et al. (2002) |
| *Lyophyllum semitale* | IE-BSG-HC85/13 | – | AF042581 | – | EF421002 | EF421068 | not indicated | Moncalvo et al. (2000); Hofstetter et al. (2002) |
| *Macrocybe crassa* | SFSU-F 024256 | MN017540 | MN017480 | MN017587 | – | MN026907 | Thailand | Vizzini et al. (2020a) |
| *Macrocybe crassa* (*"Lyophyllum praslinense"*) | AMB 10295 | MN017539 | MN017479 | MN017586 | MN018852 | – | Seychelles | Vizzini et al. (2020a) |
| *Macrocybe crassa* (*"Lyophyllum praslinense"*) | IFO31860 | – | AF042591 | – | – | – | not indicated | Moncalvo et al. (2000) |
| *Macrocybe sardoa* (*"Macrocybe titans"*) | MCVE 29083a | MN017542 | MN017481 | MN017588 | **–** | – | Italy | Vizzini et al. (2020a) |
| *Macrocybe sardoa* (*"Macrocybe titans"*) | MCVE 29083b | MN017543 | MN017482 | – | **–** | – | Italy | Vizzini et al. (2020a) |
| *Macrocybe titans* | K 55023 | MN017544 | MN017483 | – | – | – | Puerto Rico | Vizzini et al. (2020a) |
| *Macrocybe titans* | FLAS-F-58974 | MN017545 | MN017484 | MN017589 | – | MN026908 | USA | Vizzini et al. (2020a) |
| *Macrocybe titans* | FLAS-F-59217 | MN017546 | MN017485 | MN017590 | MN018853 | MN026909 | USA | Vizzini et al. (2020a) |
| *Macrocystidia cucumis* | AFTOL-ID 1343/HKAS 31464 | – | DQ094787 | DQ089014 | – | – | not indicated | Ge ZW, Matheny PB, Yang ZL and Hibbett D S, AFTOL, direct submission |
| *Musumecia alpina* | Tang-1778 | – | KR909102 | – | – | KR909096 | China | Li et al. (2016) |
| *Musumecia bettlachensis* | TO HG2284 | – | JF926521 | KJ681069 | KJ681060 | KJ681082 | Switzerland | Vizzini et al. (2011) |
| *Musumecia vermicularis* | LUG 18975 | – | KJ681037 | KJ681070 | KJ681061 | KJ681083 | France | Alvarado et al. (2015) |
| *Myochromella inolens "Tephrocybe inolens"* | CBS 330.85 | – | AF223201 | – | EF421004 | EF421071 | Switzerland | Moncalvo et al. (2002); Hofstetter et al. (2002) |
| *Neohygrophorus angelesianus* | AFTOL-ID 1719/WTU PBM482 | – | DQ470814 | DQ457698 | – | – | USA | Matheny et al. (2006) |
| *Notholepista subzonalis* | GB 0087013 | – | KJ417208 | KJ417167 | KJ424385 | – | Sweden | Sánchez-García et al. (2014) |
| *Ossicaulis lignatilis* | DUKE 483/D604 | – | AF261397 | AF334923 | DQ825410 | EF421072 | USA | Moncalvo et al. (2002); Hibbett and Donoghoue (2001); Hofstetter et al. (2002) |
| *Pogonoloma macrocephalum* | TENN 037026/MOS69/85 | – | KJ417209 | KJ417168 | – | – | Austria | Sánchez-García et al. (2014) |
| *Pogonoloma spinulosum* | K(M) 107286 | – | KJ417238 | KU058571 | KJ424401 | – | United Kingdom | Sánchez-García et al. (2014) |
| *Porpoloma portentosum* | MICH 00011834 strain Singer M149 | KJ417300 | – | – | – | – | Argentina | Sánchez-García et al. (2014) |
| *Porpoloma portentosum* | FL/MES531 | KJ417298 | KJ417210 | KU058572 | KJ424386 | – | Chile | Sánchez-García et al. (2014) |
| *Porpoloma portentosum* | NYBG REH5788 | KJ417299 | KJ417211 | – | KJ424387 | – | Argentina | Sánchez-García et al. (2014) |
| *Porpoloma sejunctum* | MICH 01183 | KJ417302 | – | – | – | – | Argentina | Sánchez-García et al. (2014) |
| *Porpoloma sejunctum* | CONC F0416 | KJ417301 | KJ417212 | KU058573 | KJ424388 | – | Chile | Sánchez-García et al. (2014) |
| *Porpoloma sejunctum*/Uncultured fungus clone | Root sample c6b7.10Trich.ARG_N01 | JX316261 | – | – | – | – | Argentina | Nouhra E and Tedersoo L, Community of ectomycorrhizal fungi of *Nothofagus* spp. in Argentina, unpublished |
| *Porpoloma* sp. 1 | TENN 065473 | KJ417304 | KJ417214 | KJ417170 | KJ424390 | – | Chile | Sánchez-García et al. (2014) |
| *Porpoloma* sp. 2 | TENN 065358 | KJ417303 | KJ417213 | KJ417169 | KJ424389 | – | Australia | Sánchez-García et al. (2014) |
| *Porpoloma terreum* | MICH 011746 | KJ417307 | – | – | – | – | Argentina | Sánchez-García et al. (2014) |
| *Porpoloma terreum* | CONC F0030 | KJ417306 | KJ417216 | – | – | – | Chile | Sánchez-García et al. (2014) |
| *Porpoloma terreum* | NYBG REH5830 | KJ417305 | KJ417215 | – | KJ424391 | – | Chile | Sánchez-García et al. (2014) |
| *Porpoloma terreum* | Root sample C8M7.1Trich.ARG_N02 | JX316283 | – | – | – | – | Argentina | Nouhra E and Tedersoo L, direct submission |
| *Porpoloma terreum* | Root sample p6m4.21Trich.ARG_N02 | JX316318 | – | – | – | – | Argentina | Nouhra E and Tedersoo L, direct submission |
| *Porpoloma terreum* | Root sample p7b4.21Trich.ARG_N02 | JX316344 | – | – | – | – | Argentina | Nouhra E and Tedersoo L, direct submission |
| *Pseudoarmillariella ectypoides* | AFTOL-ID 1557/WTU PBM1588 | – | DQ154111 | DQ465341 | DQ474127 | GU187733 | USA | Matheny et al. (2006); Binder et al. (2010); Matheny PB, Curtis JM and Hibbett DS; Matheny PB, Curtis JM and Ammirati JF; Curtis JM, Matheny PB, Ammirati JF and Hibbett DS, AFTOL, direct submission |
| *Pseudobaeospora* aff. *celluloderma* | TENN 067659/ECV5550 | KU058501 | KU058538 | KU058574 | KU139001 | – | USA | Sánchez-García and Matheny (2017) |
| *Pseudobaeospora aphana* | MushroomObserver.org/268227 | MH298912 | – | – | – | – | USA | GenBank, A Rockefeller, direct submission |
| *Pseudobaeospora brunnea* | AH 49303 | OP375152 | – | – | – | – | Spain (Canary Islands) | Bañares Baudet and Moreno (2022) |
| *Pseudobaeospora calcarea* | O-F:22037 | UDB036661 | – | – | – | – | Norway | UNITE, T Læssøe & A Molia 263f-2013 |
| ***Pseudobaeospora calcarea*** | **PAM06090111/FR2013078** | **PQ062274** | **–** | **–** | **–** | **–** | **Switzerland** | **This study** |
| *Pseudobaeospora calcarea* | O-F:21860 | UDB036674 | – | – | – | – | Norway | UNITE, A Molia & T Læssøe 242w-2013 |
| ***Pseudobaeospora celluloderma*** | SAV-F:3516 | **ON791268** | **–** | **ON791259** | – | – | **Slovakia** | **This study** |
| ***Pseudobaeospora cyanea*** | **GDOR M3986/GC122** | **OR903207** | **OR903221** | **OR889667** | **OR901918** | – | **Italy** | **This study** |
| *Pseudobaeospora cyanea* | GDOR M3986 | MT271829 | MT889638 | – | – | – | Italy | Gisotti et al. (2021) |
| ***Pseudobaeospora deceptiva*** | **MCVE 4905** | **ON791274** | **ON791280** | **ON791264** | **–** | **–** | **Italy** | **This study** |
| ***Pseudobaeospora deceptiva*** | **MCVE 15315 Holotype** | **ON791275** | **ON791281** | **ON791265** | **–** | **–** | **Italy** | **This study** |
| *Pseudobaeospora deckeri* | UCSC 7451 | JF898319 | – | – | – | – | USA | Schwarz (2012) |
| *Pseudobaeospora deckeri* | Mushroom Observer # 270813 | MF144428 | – | – | – | – | USA | GenBank, A Rockefeller, direct submission |
| ***Pseudobaeospora jamonii*** | **AQUI 10322 Neotype** | **OR903208** | **OR903222** | **–** | **–** | **–** | **Italy** | **This study** |
| ***Pseudobaeospora laguncularis* var. *denudata*** | **PAM99101004/FR2013079** | **PQ062275** | **–** | **–** | **–** | **–** | **France** | **This study** |
| *Pseudobaeospora lilacina* | SYAU-FUNGI 009 Holotype | KU528840 | KU528836 | – | – | – | USA | WU et al. (2017) |
| *Pseudobaeospora lilacina* | SYAU-FUNGI 010 | KU528842 | KU528838 | – | – | – | China | WU et al. (2017) |
| *Pseudobaeospora lilacina* | SYAU-FUNGI 011 | KU528841 | KU528837 | – | – | – | China | WU et al. (2017) |
| *Pseudobaeospora lilacina* | HMIGD 5609 | KX266951 | – | – | – | – | China | WU et al. (2017) |
| ***Pseudobaeospora mutabilis*** | **SAV-F:20580** | **ON791270** | **ON791277** | **ON791261** | **ON831383** | **PP928946** | **Slovakia** | **This study** |
| ***Pseudobaeospora mutabilis*** | **SAV-F:20589** | **ON791271** | **ON791278** | **ON791262** | **ON831384** | **–** | **Slovakia** | **This study** |
| ***Pseudobaeospora mutabilis*** | **SAV-F:3518 Holotype** | **OR903209** | **OR903223** | **–** | **–** | **–** | **Slovakia** | **This study** |
| ***Pseudobaeospora mutabilis*** | **SAV-F:3524** | **OR903210** | **OR903224** | **–** | **–** | **–** | **Slovakia** | **This study** |
| ***Pseudobaeospora pillodii*** | **LE 254346** | **–** | **OR903227** | **OR889669** | **–** | **OR901924** | **Russia** | **This study** |
| ***Pseudobaeospora pillodii*** | **O-F:258872** | **OR903213** | **OR903228** | **OR889670** | **OR901920** | **OR901925** | **Norway** | **This study** |
| *Pseudobaeospora pillodii* | O-F:258872 | NOBAS8420-21 | – | – | – | – | Norway | BOLD SYSTEMS |
| ***Pseudobaeospora pillodii*** | **KRAM-F:53298a** | **OR903211** | **–** | **OR889668** | **OR901919** | **OR901923** | **Poland** | **This study** |
| ***Pseudobaeospora pillodii*** | **KRAM-F:53314a** | **OR903212** | **OR903226** | **–** | **–** | **–** | **Poland** | **This study** |
| ***Pseudobaeospora pillodii*** | **LIP PAM06082703/FR2013113 Epitype** | **PQ062276** | – | – | – | – | **France** | **This study** |
| ***Pseudobaeospora pillodii* (“*S*. *celluloderma”*)** | **UPS-F 623041** | **ON791269** | **–** | **ON791260** | **–** | **–** | **Sweden** | **This study** |
| ***Pseudobaeospora pillodii*** | **LE 254346** | **ON791276** | **–** | **–** | **–** | **–** | **Russia** | **This study** |
| ***Pseudobaeospora pillodii*** | **KRAM-F:53298 b** | **ON791272** | **ON791279** | **ON791263** | **ON831385** | **–** | **Poland** | **This study** |
| ***Pseudobaeospora pillodii*** | **KRAM-F:53314 b** | **ON791273** | **–** | **–** | **–** | **–** | **Poland** | **This study** |
| *Pseudobaeospora pyrifera* | TUF 111505 | UDB034593 | – | – | – | – | Estonia | UNITE, T Ploompuu |
| *Pseudobaeospora pyrifera* | TUF 132017 | UDB0799048 | – | – | – | – | Estonia | UNITE T Ploompuu, |
| ***Pseudobaeospora pyrifera*** | **AMB 18729** | **OR903214** | **OR903229** | **OR889671** | **–** | **–** | **Italy** | **This study** |
| ***Pseudobaeospora pyrifera*** | **AMB 18730** | **OR903215** | **OR903230** | **OR889672** | **OR901921** | **OR901926** | **Italy** | **This study** |
| *Pseudobaeospora pyrifera* | O-F:245759 | UDB037347 | – | – | – | – | Norway | UNITE, T. Læssøe |
| *Pseudobaeospora pyrifera* | REG Isotype | AF391034 | AY176457 | – | – | – | Germany | Vellinga (2001) |
| ***Pseudobaeospora pyrifera* (*“Psudobaeospora pillodii"*)** | **GB 0160633** | **OR903216** | **OR903231** | **OR889673** | **OR901922** | **–** | **Sweden** | **This study** |
| ***Pseudobaeospora* sp.** | **LIP PAM14082601** | **PQ062277** | **–** | **–** | **–** | **–** | **France** | **This study** |
| *Pseudobaeospora* sp*.* | 10021818 | MH020190 | – | – | – | – | USA | Thies J, Tighe D, Dolejsi MK and Gottlieb L, direct submission |
| *Pseudobaeospora* sp*.* | TENN 061545 | MK268233 | – | – | – | – | USA | Ovrebo et al. (2019) |
| *Pseudobaeospora* sp*.* | TENN 064496/ECV4061 | MG663297 | MF797690 | – | – | – | USA | Matheny PB, Swenie RA and Hobbs AM, direct submission |
| *Pseudobaeospora* sp*.* | TENN 067672/ECV5553 | KU058502 | KU058539 | – | – | – | USA | Sánchez-García and Matheny (2017) |
| *Pseudobaeospora* sp. | TENN 070699/CCB143666 | KU058500 | KU058537 | – | KU139000 | – | USA | Sánchez-García and Matheny (2017) |
| *Pseudobaeospora* sp*.* | TUF 110801 | UDB013451 | – | – | – | – | Australia | UNITE, GM Gates |
| *Pseudobaeospora* sp*.* | TUF 123953 | UDB039729 | – | – | – | – | Seychelles | UNITE, U Kõljalg |
| *Pseudobaeospora* sp*.* | REH 1979 | UDB023460 | – | – | – | – | USA | UNITE, RE Halling |
| *Pseudobaeospora* sp*.* | O-F:22005 | UDB036654 | – | – | – | – | Norway | UNITE, A Molia |
| *Pseudobaeospora* sp*.* | MEL 2300736 | OQ535386 | – | – | – | – | Australia | Craig et al. (2023) |
| *Pseudobaeospora* sp*.* | MEL 2363203 | OQ457540 | – | – | – | – | Australia | Craig et al. (2023) |
| *Pseudobaeospora* sp*.* | MEL 2363205 | OQ535391 | – | – | – | – | Australia | Craig et al. (2023) |
| *Pseudobaeospora* sp*.* | MEL 2524974 | OQ535388 | – | – | – | – | Australia | Craig et al. (2023) |
| *Pseudobaeospora* sp*.* | MEL 2525018 | OQ535389 | – | – | – | – | Australia | Craig et al. (2023) |
| *Pseudobaeospora* sp*.* | MEL 2525020 | OQ535390 | – | – | – | – | Australia | Craig et al. (2023) |
| *Pseudobaeospora* sp*.* | FLAS-F-68471 | OM672808 | – | – | – | – | USA | Healy R and Lemmond B, direct submission |
| *Pseudobaeospora* sp*.* | bio-material iNAT:30847449 | MZ269236 | – | – | – | – | USA | GenBank, Taylor GM, direct submission |
| *Pseudobaeospora* sp*.* | G4422 (Soil Sample) | UDB0618517 | – | – | – | – | Estonia | UNITE, L Tedersoo et al. |
| *Pseudobaeospora* sp*.* | G4779 (Soil Sample) | UDB0519248 | – | – | – | – | Estonia | UNITE, L Tedersoo et al. |
| *Pseudobaeospora* sp*.* | MushroomObserver.org/265707 | MH304401 | – | – | – | – | USA | GenBank, Rockefeller A, direct submission |
| *Pseudobaeospora* sp*.* | MushroomObserver.org/335838 | OM655256 | – | – | – | – | Mexico | GenBank, Rockefeller A, direct submission |
| *Pseudobaeospora* sp*.* | TUE000265 (Soil Sample) | UDB05320705 | – | – | – | – | Estonia | UNITE, L Tedersoo et al. |
| *Pseudobaeospora* sp*.* | TUE000265 (Soil Sample) | UDB05320706 | – | – | – | – | Estonia | UNITE, L Tedersoo et al. |
| *Pseudobaeospora* sp*.* | TUE000405 (Soil Sample) | UDB05320708 | – | – | – | – | Portugal | UNITE, L Tedersoo et al. |
| *Pseudobaeospora* sp*.* | TUE000405 (Soil Sample) | UDB05320713 | – | – | – | – | Portugal | UNITE, L Tedersoo et al. |
| *Pseudobaeospora* sp*.* | TUE000405 (Soil Sample) | UDB05320714 | – | – | – | – | Portugal | UNITE, L Tedersoo et al. |
| *Pseudobaeospora* sp*.* | TUE000405 (Soil Sample) | UDB05320715 | – | – | – | – | Portugal | UNITE, L Tedersoo et al. |
| *Pseudobaeospora* sp*.* | TUE000894 (Soil Sample) | UDB05320716 | – | – | – | – | USA | UNITE, L Tedersoo et al. |
| *Pseudobaeospora* sp*.* | TUE000894 (Soil Sample) | UDB05320717 | – | – | – | – | USA | UNITE, L Tedersoo et al. |
| *Pseudobaeospora* sp*.* | TUE002388 (Soil Sample) | UDB05320721 | – | – | – | – | Georgia | UNITE, L Tedersoo et al. |
| *Pseudobaeospora* sp*.* | TUE002425 (Soil Sample) | UDB05320724 | – | – | – | – | Georgia | UNITE, L Tedersoo et al. |
| *Pseudobaeospora* sp*.* | TUE002425 (Soil Sample) | UDB05320725 | – | – | – | – | Georgia | UNITE, L Tedersoo et al. |
| *Pseudobaeospora* sp*.* | TUE002760 (Soil Sample) | UDB05320730 | – | – | – | – | Papua New Guinea | UNITE, L Tedersoo et al. |
| *Pseudobaeospora* sp*.* | TUE003102 (Soil Sample) | UDB05320718 | – | – | – | – | USA | UNITE, L Tedersoo et al. |
| *Pseudobaeospora* sp*.* | TUE003102 (Soil Sample) | UDB05320737 | – | – | – | – | USA | UNITE, L Tedersoo et al. |
| *Pseudobaeospora* sp*.* | TUE003102 (Soil Sample) | UDB05320752 | – | – | – | – | USA | UNITE, L Tedersoo et al. |
| *Pseudobaeospora stevensii* | CA FUNDIS iNat148539508 | OR853473 | – | – | – | – | USA | GenBank, G D'Elia et al., CA FUNDIS |
| *Pseudobaeospora stevensii* | CA FUNDIS iNat148576664 | OR881209 | – | – | – | – | USA | GenBank, G D'Elia et al., CA FUNDIS |
| *Pseudobaeospora stevensii* | CA FUNDIS iNaturalist_155104955 | OR750603 | – | – | – | – | USA | GenBank, G D'Elia et al., CA FUNDIS |
| *Pseudobaeospora stevensii* | S.D. Russell ONT WCMB23 iNaturalist # 147497163 | OR168830 | – | – | – | – | USA | GenBank, G D'Elia et al., CA FUNDIS |
| *Pseudobaeospora taluna* | MEL 2363200 | OQ457539 | – | – | – | – | Australia | Craig et al. (2023) |
| *Pseudobaeospora taluna* | MEL 2367169 | OQ535387 | – | – | – | – | Australia | Craig et al. (2023) |
| *Pseudobaeospora taluna* | MEL 2367169 | OQ552854 | – | – | – | – | Australia | Craig et al. (2023) |
| *Pseudobaeospora taluna* | MEL 2446695 | OQ457537 | – | – | – | – | Australia | Craig et al. (2023) |
| *Pseudobaeospora taluna* | MEL 2525019 | OQ457538 | – | – | – | – | Australia | Craig et al. (2023) |
| ***Pseudobaeospora terrayi*** | **SAV-F:20813** | **ON791267** | **–** | **–** | **–** | **–** | **Slovakia** | **This study** |
| ***Pseudobaeospora terrayi*** | **SAV-F:3317 Holotype** | **ON791266** | **–** | **–** | **–** | **–** | **Slovakia** | **This study** |
| *Pseudobaeospora wipapatiae* | HAW-F-00369/bio-material iNAT:34223688 | MW018883 | – | – | – | – | USA (Hawaii) | Stallman J et al., Mycoflora of Hawaii-2019 |
| *Pseudobaeospora wipapatiae* | SFSU-F DED 8605 Holotype | NR_158893 | NG_060108 | – | – | – | USA (Hawaii) | Desjardin et al. (2014) |
| *Pseudoclitocybe cyathiformis* | AFTOL-ID 1998/WTU JFA 12811 | – | EF551313 | GU187659 | GU187815 | GU187742 | USA | Binder et al. (2010) |
| *Pseudoclitocybe obbata* | AMB 18231 | – | MG321403 | – | MG334551 | MG334545 | Italy | Alvarado et al. (2015) |
| *Pseudoclitopilus rhodoleucus* | KUN-HKAS 105563 | – | MZ714594 | – | – | – | China | Sánchez-García et al. (2014) |
| *Pseudoclitopilus rhodoleucus* | GB 0110967-TK03/203 | KP453696 | KJ417218 | KU058577 | KJ424393 | – | Sweden | Sánchez-García et al. (2014); Sánchez-García and Matheny (2017) |
| *Pseudolaccaria fellea "Clitocybe fellea"* | WTU 006240 | MN017549 | MN017487 | MN017591 | – | MN026911 | USA | Vizzini et al. (2020a) |
| *Pseudolaccaria fellea "Clitocybe fellea"* | PBM1439 | – | EF561629 | – | – | – | USA | Matheny PB, Hibbett DS and Ammirati JF, AFTOL, direct submission |
| *Pseudolaccaria fellea "Clitocybe fellea"* | SA478 | – | EF561630 | – | – | – | USA | Matheny PB, Hibbett DS and Ammirati JF, AFTOL, direct submission |
| *Pseudolaccaria pachyphylla* | GB-0066637 | KU058504 | KU058542 | KU058579 | KU139006 | – | Sweden | Sánchez-García and Matheny (2017) |
| *Pseudolaccaria pachyphylla* | LIP LYK14011703 | – | MN017488 | – | MN018854 | MN026912 | France | Vizzini et al. (2020a) |
| *Pseudoomphalina kalchbrenneri* | GB 0066625 | – | KU058541 | KU058578 | KU139005 | – | Sweden | Sánchez-García and Matheny (2017) |
| *Pseudoporpoloma pes-caprae* | TO AV20915 | KU255195 | KU255196 | – | – | – | Italy | Vizzini et al. (2016) |
| *Pseudoporpoloma pes-caprae* | TENN 037121 strain MOS67/265 | KJ417297 | – | – | – | – | Poland | Sánchez-García et al. (2014) |
| ***Pseudoporpoloma pes-caprae*** | **CF PORP27080802** | KU255192 | KU255193 | **OR889674** | KU255194 | **OR901927** | France | Vizzini et al. (2016); **this study** |
| *Pseudotricholoma metapodium* | GB 0066422 | KJ417309 | KJ417220 | – | KJ424395 | – | Sweden | Sánchez-García et al. (2014) |
| *Pseudotricholoma metapodium* | GB EL155/09 | KJ417310 | – | – | KJ424396 | – | Norway | Sánchez-García et al. (2014) |
| *Pseudotricholoma metapodium* | K/AH22102006 | KJ417308 | KJ417219 | KJ417171 | KJ424394 | – | United Kingdom | Sánchez-García et al. (2014) |
| *Pseudotricholoma umbrosum* | NYBG 00505218 | KJ417312 | KJ417222 | KU058580 | KJ424397 | – | USA | Sánchez-García et al. (2014) |
| *Pseudotricholoma umbrosum* | MICH 012331 | KJ417314 | – | – | – | – | Canada | Sánchez-García et al. (2014) |
| *Pseudotricholoma umbrosum* | TENN 052643 | KJ417311 | KJ417221 | – | – | – | USA | Sánchez-García et al. (2014) |
| *Pseudotricholoma umbrosum* | TENN 064489 | KJ417315 | KJ417224 | – | KJ424398 | – | USA | Sánchez-García et al. (2014) |
| *Pseudotricholoma umbrosum* | CORT TJB7179 | KJ417313 | KJ417223 | – | – | – | USA | Sánchez-García et al. (2014) |
| *Rhizocybe alba* | HMJAU 36000 Holotype | – | KY285030 | – | KY287804 | KY287803 | China | Ding et al. (2017) |
| *Rhizocybe pruinosa* | AH 44073 | – | KJ681038 | KJ681071 | KJ681062 | KJ681084 | Spain | Alvarado et al. (2015) |
| *Rhizocybe vermicularis* | AH 44078 | – | KJ681039 | KJ681072 | KJ681063 | KJ681085 | Italy | Alvarado et al. (2015) |
| *Rhodocybe aureicystidiata* | WTU PBM 1902 | – | AY380407 | – | AY337412 | – | USA | Matheny (2005) |
| *Rhodocybe griseoaurantia* | CAL 1324 | – | KX083574 | – | KX083568 | – | India | Hyde et al. (2016) |
| *Rhodocybe indica* | CAL 1323 | – | KX083572 | – | KX083566 | – | India | Hyde et al. (2016) |
| *Rhodocybe lateritia "Clitopilus lateritius"* | G. Gates E2036-Co-David 418/CORT E1589 | – | HM164410 | – | KC816942 | KC816852 | Australia | Co-David et al. (2009); Kluting et al. (2014) |
| *Rhodocybe luteobrunnea* | CAL 1322 | – | KX083573 | – | KX083567 | – | India | Hyde et al. (2016) |
| *Rhodocybe pallidogrisea "Clitopilus pallidogriseus"* | ME Noordeloos 2004032-isolate 118/CORT E652 | – | GQ289216 | – | GQ289283 | KC816875 | Australia | Co-David et al. (2009); Kluting et al. (2014) |
| *Rhodocybe paurii* | CORT JM99/233 | – | AY286004 | – | KC816969 | KC816876 | India | Moncalvo et al. (2004); Kluting et al. (2014) |
| *Rhodocybe truncata* | CBS 482.50 | – | AF223167 | – | EF421019 | – | not indicated | Moncalvo et al. (2002); Hofstetter et al. (2002) |
| *Rhodophana nitellina "Clitopilus nitellinus"* | ME Noordeloos 200435 | – | GQ289215 | – | GQ289282 | – | Austria | Co-David et al. (2009) |
| *Rhodophana stangliana "Clitopilus stanglianus"* | N. Dam 05094 | – | GQ289218 | – | GQ289285 | – | Switzerland | Co-David et al. (2009) |
| *Sagaranella tylicolor "Tephrocybe tylicolor"* | BSI 92.245 | – | AF223195 | – | EF421006 | EF421074 | not indicated | Moncalvo et al. (2002); Hofstetter et al. (2002) |
| *Singerocybe adirondackensis "Clitocybe adirondackensis"* | TENN 64652/64660/60310 | – | JX514103 | HQ728531 | JX514140 | KF208440 | USA | Qin et al. (2014); Matheny et al. (2014) |
| *Singerocybe alboinfundibuliformis* | HKAS 74716 | – | JX514106 | – | JX514138 | KF208433 | China | Qin et al. (2014) |
| *Singerocybe clitocyboides* | HKAS 75453 | – | JX514113 | – | JX514149 | KF208444 | Australia | Qin et al. (2014) |
| *Singerocybe phaeophthalma* | TO AV071112a | – | KJ681041 | KJ681074 | KJ681064 | KJ681087 | Italy | Alvarado et al. (2015) |
| *Singerocybe umbilicata* | HKAS 77290 | – | KF208457 | – | KF208460 | KF208438 | China | Qin et al. (2014) |
| *Sphagnurus paluster "Tephrocybe palustris" "Lyophyllum palustris"* | CBS 717.87 | – | AF223200 | – | EF421007 | EF421075 | Switzerland | Moncalvo et al. (2002); Hofstetter et al. (2002) |
| *Suillus pictus* | AFTOL-ID 717/MB 03-002 | AY854069 | AY684154 | AY662659 | AY786066 | AY883429 | USA | Binder M, Matheny PB and Hibbett DS, AFTOL, direct submission |
| *Tephrocybe rancida* | CBS 204.47 |  | AF223203 |  | EF421008 | EF421076 | Switzerland | Moncalvo et al. (2002); Hofstetter et al. (2002) |
| *Tephroderma fuscopallens* | LUG 18989 |  | KJ701333 | KJ701331 |  | KJ701329 | Switzerland | Musumeci and Contu (2014) |
| *Tephroderma fuscopallens* | EM4789-12 |  | KJ701332 | KJ701330 |  | KJ701328 | Switzerland | Musumeci and Contu (2014) |
| *Tricholoma elegans* | TENN 063711 | KJ417316 | KJ417226 |  |  |  | New Zealand | Sánchez-García et al. (2014) |
| *Tricholoma equestre* | EqFr3 | HM590872 |  |  |  |  | not indicated | Moukha et al. (2013) |
| *Tricholoma flavovirens* | CORT 11CA038 | – | KJ021704 | – | KC816997 | – | USA | Largent et al. (2014); Kluting et al. (2014) |
| *Tricholoma flavovirens* | AP40 | EU186310 | – | – | – | – | Portugal | Portugal A, Bidartondo M and Rodriguez-Echeverria S, direct submission |
| *Tricholoma inamoenum* | KMS 249 | AF377246 | – | – | – | – | USA | Bidartondo and Bruns (2002) |
| *Tricholoma inamoenum* | REG MB 96-071 | – | AY293215 | AY293161 | – | – | not indicated | Binder et al. (2005) |
| *Tricholoma matsutake* (= *T. nauseosum*) | Tn9 | AB188557 | U62964 | U62538 | – | – | Sweden/not indicated | Matsushita et al. (2005); Hwang and Kim (1995) |
| *Tricholoma myomyces* | KMS 589 | DQ825428 | U76459 | DQ367422 | DQ367436 | DQ367429 | not indicated | Hofstetter et al. (2002); Shanks K and Vilgalys R, direct submission |
| *Tricholoma palustre* | AFTOL-ID 497/CUW PBM 2494 | DQ494699 | AY700197 | AY757267 | DQ484055 | – | USA | Hofstetter et al. (2002); Matheny et al. (2006) |
| *Tricholoma portentosum* | KMS 591 | AF357015 | U76464 | – | EF421014 | EF421084 | not indicated | Hofstetter et al. (2002); Shanks K and Vilgalys R, direct submission; Hofstetter V et al. direct submission |
| *Tricholoma saponaceum* | AFTOL-ID 672 | DQ494700 | AY647209 | AY654883 | – | – | not indicated | Matheny et al. (2006); Matheny PB and Hibbett DS, AFTOL, direct submission |
| *Tricholoma* sp. | AB122/S09 | KR819126 | – | – | – | – | Cameroon | Michaella Ebenye HCM et al. - Fungal diversity of ectomycorrhizae and sporocarps of *Gilbertiodendron dewevrei* in a Dja forest; direct submission |
| *Tricholoma* sp. | SAR1/2/90 | – | AF042592 | AF287839 | – | – | not indicated | Moncalvo et al. (2000); Hibbett et al. (2000) |
| *Tricholoma* sp. 1 | TENN 063710 | KJ417317 | KJ417227 | KJ417172 | – | – | New Zealand | Sánchez-García et al. (2014) |
| *Tricholoma* sp. 2 | TENN 063664 | KJ417318 | KJ417228 | KJ417173 | – | – | New Zealand | Sánchez-García et al. (2014) |
| *Tricholoma* sp. 3 | TENN 061065 | – | KJ417229 | KJ417174 | – | – | New Zealand | Sánchez-García et al. (2014) |
| *Tricholoma subaureum* | KMS 590 | AF357016 | U76466 | – | EF421015 | EF421085 | not indicated | Hofstetter et al. (2002); Shanks K and Vilgalys R, direct submission; Hofstetter V et al. direct submission |
| *Tricholoma subresplendens* | TENN 065679 | KJ417319 | KJ417230 | KJ417175 | – | – | USA | Sánchez-García et al. (2014) |
| *Tricholoma viridiolivaceum* | TENN 063670/PBM3093/ PDD97890 | JF706316 | JF706317 | JF706318 | JF706319 | – | USA | Baroni and Matheny (2011) |
| *Tricholomella constricta* | HC 84.75 | – | AF223188 | DQ825434 | DQ825412 | EF421079 | not indicated | Moncalvo et al. (2002); Hofstetter et al. (2002) |
| *Xerophorus dominicanus* | JBSD 127428 | MN017550 | MN017489 | – | MN018855 | MN026913 | Dominican Republic | Vizzini et al. (2020a) |
| *Xerophorus donadinii* | AMB 18222 | MN017551 | MN017490 | MN017592 | – | MN026914 | Italy | Vizzini et al. (2020a) |
| *Xerophorus donadinii* | AMB 18223 | MN017552 | MN017491 | – | – | – | Italy | Vizzini et al. (2020a) |
| *Xerophorus donadinii* | FP2014-09-11 | – | MK277665 | – | – | – | Hungary | Varga et al. (2019) |
| *Xerophorus olivascens* | AMB 18226 | MN017558 | MN017496 | – | MN018856 | MN026916 | Italy | Vizzini et al. (2020a) |
| *Xerophorus olivascens* | AMB 18227 | MN017559 | MN017497 | – | MN018857 | – | Italy | Vizzini et al. (2020a) |
| *Xerophorus olivascens* | AMB 18229 | MN017560 | MN017498 | MN017593 | MN018858 | – | Italy | Vizzini et al. (2020a) |
