# Supplementary figures and images for: ﻿Pseudobaeosporoideae, a new subfamily within the Tricholomataceae for the genus Pseudobaeospora (Agaricales, Tricholomatineae) based on morphological and molecular inference
Source: IMA Fungus. 2025 Mar 13;16:e144994. doi: 10.3897/imafungus.16.144994 (PMC11926610; doi:10.3897/imafungus.16.144994)

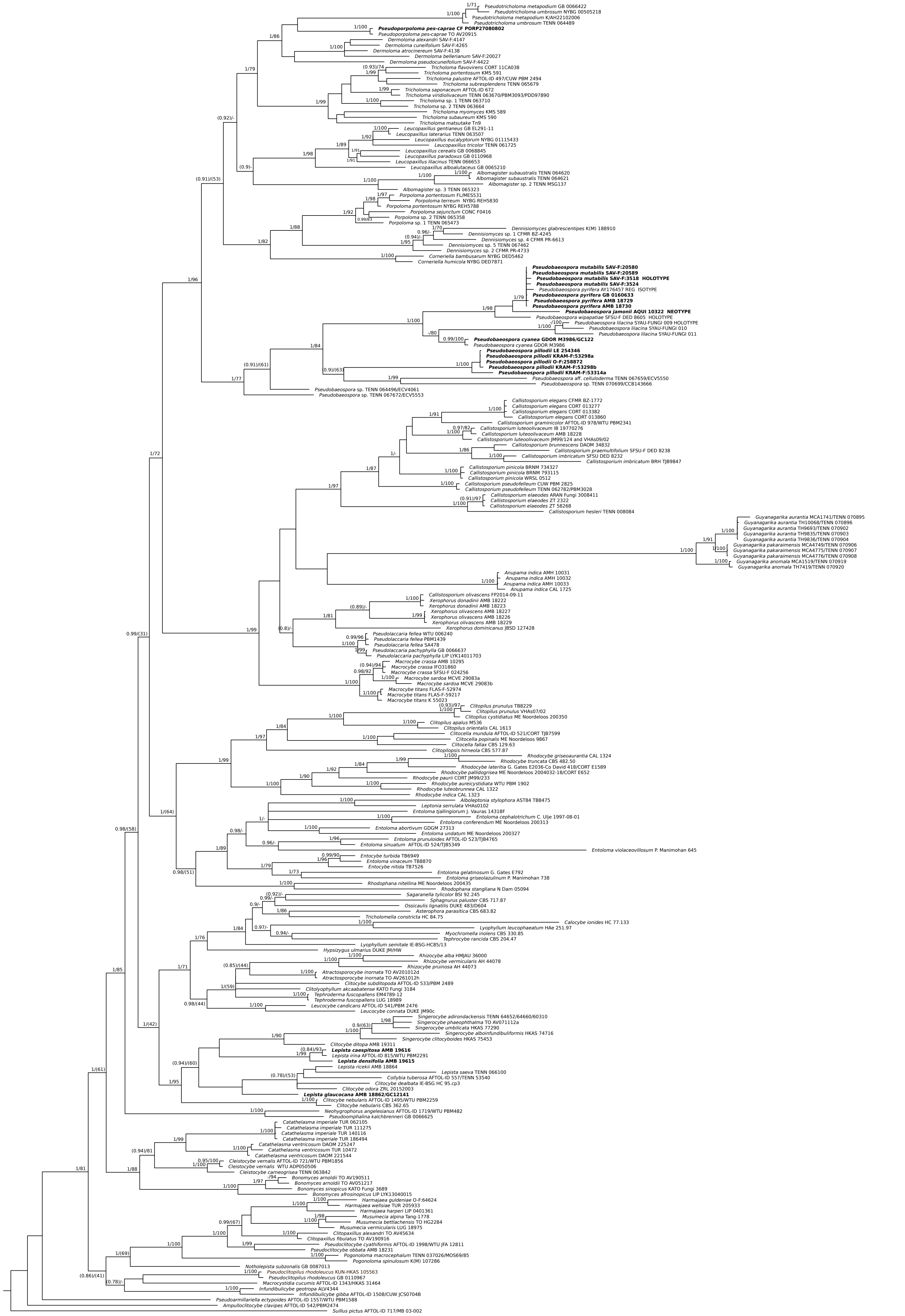

Supplement: Supplementary material 2 — Bayesian inference phylogram [file imafungus-16-e144994-s002.pdf]
